# Robust and Integrable Time-Varying Metamaterials: A Systematic Survey and Coherent Mapping

**DOI:** 10.3390/nano16030195

**Published:** 2026-01-31

**Authors:** Ioannis Koutzoglou, Stamatios Amanatiadis, Nikolaos V. Kantartzis

**Affiliations:** School of Electrical and Computer Engineering, Aristotle University of Thessaloniki, 54124 Thessaloniki, Greece; ikoutzo@ece.auth.gr (I.K.); samanati@auth.gr (S.A.)

**Keywords:** temporal metamaterials, time-varying metasurfaces, spatiotemporal modulation, photonic time crystals, nonreciprocal wave propagation, parametric amplification, frequency conversion, temporal cloaking, artificial intelligence, ultra-fast photonics

## Abstract

Time-varying or temporal metamaterials and metasurfaces, in which electromagnetic parameters are deliberately modulated in time, have emerged as a powerful route to engineer wave–matter interaction beyond what is possible in static media. By enabling the controlled exchange of energy and momentum with the fields, they underpin magnet-free nonreciprocity, low-loss frequency conversion, temporal impedance matching beyond Bode-Fano limit, and unconventional parametric gain and noise control. This survey provides a coherent framework that unifies the main theoretical and experimental developments in the area, from early analyses of velocity-modulated dielectrics to recent demonstrations of temporal photonic crystals, non-Foster temporal boundaries, and spatiotemporally driven metasurfaces relevant to nanophotonic platforms. We systematically compare time-varying permittivity, joint ε-μ modulation, time-varying conductivity, plasmas, and circuit-equivalent implementations, including stochastic and rapidly sign-switching regimes, and relate them to acoustic and quantum analogs using common figures of merit, such as conversion efficiency, isolation versus insertion loss, modulation depth and speed, dynamic range, and stability. Our work concludes by outlining key challenges, loss and pump efficiency, high-speed modulation at the nanoscale, dispersion engineering for broadband operation, and fair benchmarking, which must be addressed for robust, integrable temporal metasurfaces.

## 1. Introduction

The ability to modulate electromagnetic media in time, rather than solely in space, has opened a distinct and rapidly expanding domain within wave engineering. Time-varying (temporal) metamaterials, structures whose constitutive parameters are intentionally varied as functions of time, enable forms of wave–matter interaction that are fundamentally inaccessible in static platforms [1]. By allowing for the controlled exchange of energy and momentum between fields and the modulation, these systems support magnet-free nonreciprocity [2], linear and low-loss frequency conversion [3], temporal impedance matching [4], and unconventional regimes of parametric gain and noise shaping [5]. Recent works at optical [6], microwave [7], and acoustic [8] frequencies highlight both the conceptual breadth and the technological potential of time-varying media, particularly as advances in ultrafast materials [9,10], integrated modulators [11,12], and epsilon near-zero films bring high-speed temporal control within reach of nanophotonic systems [13,14,15,16].

The foundations of time-varying electromagnetics trace back to early analyses of velocity-modulated dielectrics [17], where temporal changes in material parameters were shown to induce shifts in frequency and wave velocity. These early treatments established the fundamental insight that a medium whose properties evolve in time does not conserve optical frequency, in contrast to spatially varying media that preserve it through translational symmetry. Building on this perspective, subsequent work examined temporal boundaries [18], abrupt temporal discontinuities [19], and modulated slabs [20], showing that waves encountering time-varying interfaces undergo reflection and refraction in the frequency (spectral) domain. These studies were essential in clarifying how temporal transitions mediate controlled energy exchange with the electromagnetic field.

As interest in these ideas escalated, more comprehensive theoretical frameworks emerged. To achieve this goal, the Floquet theory was adapted to handle periodic temporal modulation [21], revealing the formation of frequency sidebands, quasi-energy band structures, and momentum gaps that define the behavior of photonic time crystals [22,23,24]. Moreover, temporal-scattering formalisms provided complementary tools for treating broadband excitations and finite-duration modulation. In parallel, circuit representations [25] of time-varying capacitors and inductors offered intuitive analogs for parametric mixing, gain, and stability, establishing a bridge between distributed electromagnetic structures and lumped-element implementations. Such models led to a set of analytical tools capable of describing a wide range of temporally engineered media.

Recent developments have further expanded the scope of the field beyond deterministic modulation [26]. Hence, stochastic and disordered time variations have revealed new forms of localization and robustness, while rapid sign changes in permittivity have produced temporal analogs of non-Foster behavior that challenge conventional limits on bandwidth and energy storage [27]. Furthermore, spatiotemporal permittivity profiles [28,29,30,31], where time variation is combined with spatial modulation, provide another degree of control, enabling synthetic motion, magnet-free nonreciprocity, and advanced signal-routing schemes. These diverse mechanisms illustrate the conceptual richness of temporal metamaterials and their relevance across electromagnetic, acoustic, and even quantum platforms.

**Figure 1:**
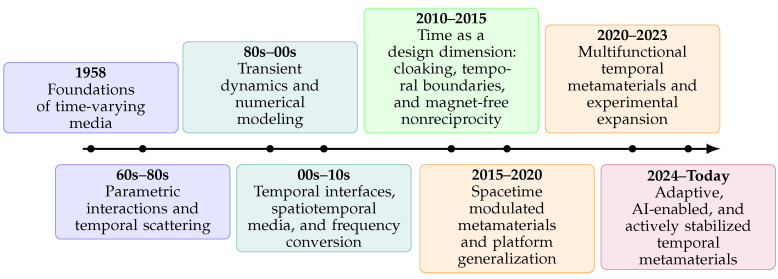
Progress timeline highlighting key milestones in the evolution of time-varying and space–time-modulated metamaterials, from early theoretical foundations to recent integrated, adaptive, and AI-enabled platforms.

Despite the growing activity, the literature remains fragmented, as many contributions address only a single form of modulation or focus on one class of physical systems, which makes it difficult to view temporal metamaterials as a unified category. This survey aims to provide such a unified perspective by assembling the main theoretical models, material platforms, and demonstrated applications into a coherent methodology. Specifically, we compare the modulation of permittivity, permeability, and conductivity, spatiotemporal media, temporal plasmas, and temporally varying circuit analogs, with particular emphasis on how these platforms mediate the interplay between gain, stability, and noise. We further relate electromagnetic treatments to developments in acoustics and quantum time-dependent media, highlighting both shared formalisms and domain-specific constraints. Throughout the survey, we emphasize cross-comparison using common figures of merit, including conversion efficiency, isolation versus insertion loss, modulation depth and speed, dynamic range, and stability thresholds, to provide a consistent basis for evaluating diverse approaches. While important reviews and roadmaps have addressed specific aspects of time-varying and space–time-modulated systems, we are not aware of prior works that pursue a comparable breadth across materials, platforms, and degrees of experimental realization within a single framework. Therefore, by presenting these topics within a single structure, this survey seeks to serve both as a conceptual map of time-varying metamaterials and as a practical guide for their robust and integrable implementation in emerging nanophotonic systems. In this context, Figure 1 provides a visual roadmap of the historical evolution and recent acceleration of time-varying metamaterials, highlighting the progression from early theoretical foundations to modern integrated and adaptive platforms.

## 2. Foundational Review Literature

A number of thorough reviews have recently examined specific aspects of wave systems with temporal or spatiotemporal modulation. Each provides valuable insight into an important subset of the field, yet their scopes remain necessarily focused on specific themes, like nonreciprocity, space–time constitutive relations, active metasurfaces, or photonic time crystals. As these works address different mechanisms, platforms, and theoretical viewpoints, the broader landscape of temporally engineered media remains fragmented. This section summarizes the main perspectives offered by these reviews and highlights how they collectively motivate the need for the unified framework of the present survey.

Sounas and Alù, in [32], reviewed how temporal modulation can break reciprocity in photonics without relying on magnetic biasing, and they did so in a mechanism-oriented and conceptually structured manner. Their discussion begins with Lorentz reciprocity and the symmetry properties of scattering matrices in linear, time-invariant, passive systems, emphasizing why magneto-optical approaches remain bulky and incompatible with integrated photonics. Time modulation is then introduced as a route to impart an effective bias and synthesize gauge fields using standard materials together with CMOS-compatible drive circuitry. Within this analysis, the authors classify nonreciprocal schemes into several families. One major class relies on traveling-wave spatiotemporal modulation, which imparts synthetic linear momentum and enables indirect photonic transitions that redistribute energy among modes in a way that depends on the propagation direction. Coupled-waveguide implementations with electrical modulators exemplify this approach and reveal trade-offs between modulation depth, device length, and isolation bandwidth. A second class uses synthetic angular momentum, in which rotating modulation patterns in resonators or loops of coupled cavities split counter-propagating eigenfrequencies, enabling circulator behavior in compact, magnet-free geometries. A third mechanism is based on direct photonic transitions produced through engineered phase accumulation in modulated waveguides or superconducting Josephson circuits, achieving isolation through asymmetric mode conversion rather than traditional phase matching. The review also covers optomechanical systems, where acoustic or mechanical degrees of freedom induce direction-dependent coupling, and connects time-modulated platforms to photonic topological insulators through synthetic magnetic fields and protected edge channels. Throughout, Ref. [32] compares these strategies in terms of device complexity, robustness, loss, and scalability, concluding that temporal modulation offers one of the most promising platforms for broadband, integrated, magnet-free nonreciprocal photonics.

In [33], Caloz and Deck-Léger characterized space–time metamaterials as the “fourth generation” of metamaterials and provided a unifying conceptual language for media whose properties vary jointly in space and time. Starting from the historical progression of metamaterials, Ref. [33] broadens the conventional notion of spatially structured, static media to include systems whose response is intrinsically governed by time modulation. They generalize bianisotropic constitutive relations to susceptibilities that may depend simultaneously on direct space and time (r,t) and on spatial and temporal frequencies (k,ω). This formulation is then organized into a space–time variance and dispersion “phase diagram”, which clarifies the relationships among different classes of medium variation and highlights when homogenization is possible or fundamentally constrained by Fourier uncertainty. The core emphasis of this review is conceptual. It identifies frequency transitions, including Doppler and generalized space–time photonic transitions, nonreciprocity induced by motion or modulation, Fizeau drag, and bianisotropy transformation as canonical phenomena in space–time systems. A notable contribution is the adoption of a relativistic space–time formulation, according to which which Minkowski diagrams and Lorentz transformations are adapted into practical tools to analyze space–time discontinuities and crystals, including subluminal, superluminal, and pure time limits. Additionally, Ref. [33] catalogues canonical space–time building blocks, such as space, time, and space–time steps, crystals, mirrors, cavities, and checkerboards, stressing the importance of truncation, synchronization, and finite-duration modulation when moving from idealized constructs to physical implementations. While the review does not focus on specific devices, it provides a rigorous vocabulary and geometric intuition that serves as a foundation for the broader, cross-platform treatment undertaken in this survey.

In the second part of their tutorial [34], Caloz and Deck-Léger shift from conceptual foundations to concrete theory and applications, presenting a unified treatment of electromagnetic scattering at boundaries and crystals that vary jointly in space and time. The exposition begins by contrasting pure space and pure time boundaries and uses these limiting cases to derive consistent electromagnetic boundary conditions for subluminal and superluminal space–time interfaces. In the subluminal regime, the interface behaves like a moving “tilted” boundary that mixes frequency and wavenumber while preserving causality. In the superluminal regime, by contrast, the interface acts as a temporal discontinuity that slices worldlines, causing familiar analogies based on moving media to break down and enabling new scattering pathways. On this foundation, the authors derive scattering and frequency-transition coefficients in a systematic hierarchy that proceeds from spatial Fresnel formulas to temporal Fresnel formulas and ultimately to their full space–time generalizations. These results show how incident monochromatic waves generate reflected and transmitted components with shifted frequencies and altered propagation directions, depending on both interface velocity and material contrast. The article then introduces space–time mirrors and cavities as new canonical structures capable of focusing, diffracting, compressing, or expanding waves in coupled space–time domains. Space–time “inverse prisms”, which convert spatial spectra into temporal ones and vice versa, provide a conceptual bridge to many of the more specialized devices seen in the recent literature. The review concludes with a broad survey of applications, including frequency multiplication, mixing via controlled space–time transitions, matching and filtering enabled by temporal impedance control, nonreciprocal behavior from traveling modulations, space–time cloaking that conceals events, and radiation engineering in space–time crystals. Overall, Ref. [34] consolidates many previously disparate formalisms into a single analytical language and maps out design tools that underpin numerous temporally modulated devices.

The evolution from static metasurfaces to actively and spatiotemporally reconfigurable metasurfaces has been reviewed by Shaltout et al. in [35], with an emphasis on both enabling technologies and the new physics that emerges once time becomes a genuine design axis. They begin by recalling that passive metasurfaces manipulate amplitude, phase, and polarization through fixed subwavelength patterning, but their functionality is frozen after fabrication. Active metasurfaces, by contrast, allow the local optical response to be dynamically reconfigured in space and time, enabling engineered phase profiles that coexist with time-dependent dispersion, refractive index, and effective motion. A substantial portion of the analysis covers materials and mechanisms that support strong, fast, and reversible modulation in deeply subwavelength cells. These include electrical gating and photocarrier excitation in transparent conducting oxides and materials that are two-dimensional (including epsilon-near-zero (ENZ) operation and graphene-based platforms), materials that undergo phase change, such as GST and VO_2_, metasurfaces actuated by MEMS and mechanically reconfigurable, magneto-optic platforms, architectures loaded with liquid crystals, electrochemical metallization, and optical nonlinearities enhanced by resonance in metals, oxides, and high-index dielectrics. For each of these classes, Ref. [35] discusses the achievable tuning strengths and modulation speeds, and demonstrate devices such as mirrors that are tunable by voltage, varifocal metalenses, stretchable holograms, and dynamically reconfigurable surfaces for beam steering and polarization control. The second half of the review focuses on genuinely spatiotemporal metasurfaces, where modulation rates approach or exceed the signal bandwidth, activating Maxwell’s space–time duality. The authors explain how rapid temporal modulation can enable frequency translation via time refraction, Doppler shifts without mechanical motion, and the generation of phase-conjugated or negatively refracted beams from ultrathin sheets. They connect these effects to the breaking of Lorentz reciprocity, nonreciprocal Snell’s laws, synthetic gauge fields, and photonic topological phases. In summary, the review identifies both opportunities and bottlenecks, including the challenge of achieving large modulation in nanoscale elements, scaling to large pixel counts with manageable power and wiring, as well as co-optimizing material and nanoresonator design.

Galiffi et al., in [36], presented a broad roadmap for time-varying media from a photonics perspective, emphasizing temporal modulation as a genuine and versatile degree of freedom for wave control. The review begins with temporal interfaces and “time switching” in simple dielectric media, highlighting the duality achieved with spatial scattering. Temporal boundary conditions conserve momentum while enabling frequency conversion and amplitude changes, forming the basis for temporal slabs and layered time structures. The authors describe temporal Fabry–Pérot analogs, antireflection temporal coatings, and temporally engineered gain or loss profiles, then discuss extensions to anisotropic and dispersive media, where temporal switching can steer beams, realize “inverse prism” behavior, or enable temporal Brewster phenomena. A major part of the review focuses on photonic time crystals, defined as systems with parameters that are modulated periodically or quasi-periodically in time. Floquet theory reveals momentum gaps (*k*-gaps) and parametric amplification, which [36] relates to the temporal topological phases realized in the combined frequency and time domain. Moreover, they explore synthetic frequency dimensions formed from cascaded temporal mode coupling, as well as non-Hermitian and disordered time-varying systems whose temporal modulation of gain and loss connects to parity–time (PT) symmetry, exceptional points, and the time-domain analogs of Anderson localization. The review then expands to fully spatiotemporal metamaterials, where traveling-wave modulations generate tilted space–time band diagrams, synthetic motion, and Fizeau-drag effects, leading naturally to nonreciprocal scattering and asymmetric band gaps. Moreover, Ref. [36] covers homogenization theories for traveling-wave modulations, spatiotemporal crystals, and modulated metasurfaces, and they close with a survey of experimental platforms ranging from ENZ films and semiconductors to graphene, metasurfaces, and acoustic analogs. Overall, the review synthesizes temporal switching, photonic time crystals, and spatiotemporal metamaterials into a single conceptual framework while pointing to open directions in topological, non-Hermitian, and ultrafast implementations.

Collectively, these reviews offer very instructive and complementary perspectives of temporally and spatiotemporally modulated media, each illuminating a specific subset of the underlying physics or a particular class of implementations. What they jointly reveal, however, is that the field remains conceptually diverse and methodologically fragmented, with different works emphasizing different mechanisms, modeling approaches, and frequency regimes. As a result, it is difficult to obtain a coherent view of temporal metamaterials as a unified category of wave-engineering systems. The present survey builds on the foundations laid by these reviews and brings their perspectives together within a single method, aiming to provide an integrated, cross-platform map of time-varying media and their emerging applications.

## 3. Theoretical Foundations of Time-Varying Metamaterials

The study of time-varying and spatiotemporally modulated media rests on a set of theoretical principles that differ in important ways from those governing static or purely spatially structured systems. Temporal modulation alters the fundamental symmetries of Maxwell’s equations and enables the controlled exchange of energy and momentum between fields and the medium, giving rise to phenomena such as frequency conversion, parametric amplification, and magnet-free nonreciprocity. In recent decades, these ideas have been developed through a diverse collection of models, ranging from early analyses of time-varying dielectric slabs to modern treatments of temporal crystals, traveling-wave modulations, stochastic variations, and non-Hermitian temporal dynamics. Since these results emerged across different communities and with distinct formalisms, a coherent synthesis is essential before surveying the broader landscape of temporal metamaterials and applications. This section consolidates the principal theoretical techniques, organizing them from purely temporal to fully spatiotemporal media and from deterministic to stochastic and non-Hermitian regimes, to provide a unified basis for the developments discussed in the remainder of this work.

### 3.1. Time-Varying Media

Temporal media are systems whose constitutive parameters vary exclusively in time, without spatial modulation, so that the medium remains homogeneous at every instant while its electromagnetic response evolves dynamically. This class of structures provides the most straightforward method through which to understand how time-dependence modifies wave propagation, since translational symmetry in space is preserved and momentum remains conserved even as frequency undergoes controlled transitions. Many of the central physical effects associated with temporal metamaterials, such as time refraction, frequency conversion, energy exchange with the modulation, parametric amplification, and the formation of temporal band structures, first emerge in this impaired context. Theoretical treatments spanning from early analyses of sinusoidally modulated dielectrics to modern formulations of temporal interfaces, periodically driven media, and rapid sign changes in permittivity collectively establish the foundations on which more complex spatiotemporal and non-Hermitian systems are built. This subsection reviews these foundational models, progressing from time-varying permittivity to the co-modulation of permittivity and permeability, time-dependent conductivity, and temporally evolving plasmas, emphasizing the shared principles that underlie their behavior.

#### 3.1.1. Time-Varying Permittivity

Modulation of the permittivity [37,38,39] in time is the most fundamental and widely studied form of temporal media, and it provides the clearest setting in which frequency conversion, parametric amplification, and temporal band formation emerge. Because the medium remains spatially uniform at each instant, the wavenumber is conserved and the temporal modulation acts entirely through frequency and amplitude evolution. The works reviewed in this subsection establish the main mathematical tools, such as Mathieu theory, WKB analysis, temporal boundary conditions, and Floquet theory, that underpin much of the later literature on temporal and spatiotemporal metamaterials.

Holberg and Kunz, in [40], developed an ab initio theoretical description for plane waves in a dielectric whose permittivity is modulated purely in time, modeled as

(1) $$\varepsilon \left(t\right)=\frac{\varepsilon }{1-2p\cos(2\Omega t)}\;,$$

and show that the temporal dynamics map exactly onto Mathieu functions. Physically, this modulation acts as a parametric pump that exchanges energy with the field while preserving spatial momentum, so the resulting dynamics are governed by stability (bounded “galloping” response) versus instability (exponential temporal growth) rather than by conventional spatial Bloch modes. In an unbounded medium, the electric displacement field separates as $D\left(t,x\right)=T\left(t;k\right)\;e^{ikx}$, where T(t;k) is a Mathieu solution. The resulting “stability chart” exhibits pass regions with bounded, periodically “galloping” waves and unstable tongues where fields form standing waves that grow or decay exponentially in time at a rate μ. Growth is strongest near the center of the first instability tongue and persists no matter how small the modulation index 2p, provided the wave number *k* places the operating point inside the tongue. This establishes that an infinite time-modulated dielectric is generically unstable for those *k*-bands with growth governed entirely by Mathieu theory.

They consider a finite slab of the modulated medium embedded in a constant host and derive exact continuity conditions to determine whether exponentially growing modes can be sustained despite radiation leakage through the interfaces. Working in the small modulation limit and focusing on the first tongue, they obtain compact stability criteria in terms of the electrical slab half-thickness Kl and the index *p*. For a host matched to the unmodulated slab, no self-sustained growth exists unless

(2) $$Klp\geq \frac{\pi }{2}\;,$$

which sets a critical modulation proportional to inverse electrical thickness. More generally, with impedance mismatch, the stability bounds shift to

(3) $$Klp\geq 2\tan^{-1}\;\left(v^{-1}\right)\;\mathrm{or}\;Klp\geq 2\tan^{-1}\;\left(v\right),$$

depending on symmetry, showing that a mismatch lowers the threshold by reducing boundary radiation loss. The analysis also identifies “most favorable” slab lengths (near integral or half-integral multiples of π) where the growth exponent is extremized.

Finally, for stable slabs below the threshold, the paper derives closed-form reflection and transmission near half the pump frequency, ω≈Ω/2. With normal incidence and small *p*, the slab converts an input at ω into a reflected and transmitted pair at ω and its idler Ω−ω. In the matched case, the coefficients reduce to

(4) $$R=-\;i\;\tan\;\left(Klp\right),\;T=\frac{\pm 1}{\cos\;\left(Klp\right)},$$

revealing parametric gain without net energy input at the signal frequency. Away from exact half-pump or with a mismatch, Ref. [40] provides general expressions for the two spectral components, recovering the sonic and quasi-sonic behaviors anticipated by earlier traveling-wave theories. The results provide a unified picture that combines bulk growth governed by Mathieu functions, radiation-limited thresholds in finite slabs, and explicit small-signal conversion formulas that enable the design of oscillators, amplifiers, filters, and frequency shifters.

The general boundary value problem of a plane wave incident on a time-varying half-space was treated comprehensively in [41], where both abrupt-switching and slowly varying (WKB) permittivity profiles, are analyzed. For a slowly varying, nondispersive dielectric with phase velocity $v\left(t\right)=c/\sqrt{\epsilon_{r}\left(t\right)}$, the field inside x>0 admits the WKB form

(5) $$E\left(r,t\right)\approx A\left(t\right)\exp\;\;\left(i\omega \tau \right)u\;\left[\int_{0}^{t}\;\;v\left(t^{\prime }\right)dt^{\prime }-\hat{k_{1}}\cdot r\right]\;\;,$$

where τ is the entrance time determined by the eikonal condition and A(t) accounts for the slow amplitude scaling. The local (instantaneous) frequency follows from the phase, and at normal incidence, one obtains

(6) $$\omega_{i}=\omega \left[\frac{v(t)}{v\left(\tau \right)+r\cdot \left(d\hat{k}/d\tau \right)}\right],$$

showing the conservation of wavenumber across a temporal boundary and a deterministic chirp governed by v(t). The dispersive case is formulated from the Klein-Gordon type form

(7) $$\frac{\partial^{2}E}{\partial x^{2}}-\frac{1}{c^{2}}\frac{\partial^{2}E}{\partial t^{2}}+K^{2}\left(t\right)E=0,$$

leading to causal integral representations over temporal and spatial spectra and, in the slowly varying limit, to a Green function with time-dependent transmission factor and group velocity rays obtained by saddle-point evaluation. Altogether, the analysis provides exact constructions for step switching with temporal Fresnel-type transmission and reflection inside the medium, WKB formulas for continuous modulation that offer explicit instantaneous frequency and amplitude scaling, and an asymptotic framework for dispersive half-spaces that recovers the static limit while yielding space–time ray evolution for general ramps.

The temporal analogs of spatial refraction and reflection, arising during sudden jumps in material parameters, were formalized in [42], which introduced the concepts of time refraction and time reflection. The central kinematic rule inverts the familiar spatial picture and states that the wave vector is conserved across a temporal jump, whereas the frequency adjusts to satisfy the dispersion relation of the medium after the jump. Because the medium changes in time, energy in the optical field is not conserved, so amplitude scaling accompanies the frequency conversion. The authors emphasize that these processes are generic, independent of geometry, and arise whenever the constitutive parameters switch sufficiently fast compared to an optical cycle. Using this concept, Ref. [42] derives temporal Fresnel coefficients from macroscopic boundary conditions appropriate to time discontinuities and discusses how they differ from spatial Fresnel laws. In particular, the absence of spatial impedance mismatch does not imply unit transmission of field amplitude, since the temporal jump modifies both carrier frequency and cycle duration. The theory clarifies causality and shows how temporal interfaces compress or expand optical periods, generate backward-in-time echoes, and enable interband energy transfer without phase matching. The analysis closes by situating time refraction and time reflection within broader nonstationary electromagnetics, connecting to plasmas and photonic media undergoing rapid index changes, and it highlights prospective applications, including frequency translation without spatial phase matching, temporal cloaking windows, and parametric gain in time crystalline modulations.

Furthermore, Zurita-Sánchez et al., in [21], presented a general theory for plane-wave scattering using a slab whose permittivity is periodic in time, ε(t)=ε(t+T), and for wave propagation in the corresponding dynamic bulk. Starting from Maxwell’s equations for a nonmagnetic, lossless medium, the electric field in the bulk satisfies

(8) $$\frac{d^{2}}{dt^{2}}\;\left(\varepsilon (t)\;E(t)\right)+k^{2}c^{2}\;E\left(t\right)=0,$$

which is solved by a Bloch–Floquet ansatz $E\left(t\right)=\bar{E}\left(\omega ,t\right)\;e^{-i\omega t}$ with $\bar{E}\left(\omega ,t\right)$ periodic in *t*. Fourier expansion of ε(t) and $\bar{E}$ yields an infinite linear system whose nontrivial solution defines a dispersion relation in the form of a band structure that is periodic in ω with period Ω=2π/T. A key outcome is the appearance of forbidden wave-number gaps (*k*-gaps) at mirror planes ω=rΩ/2, in contrast with conventional spatial photonic crystals that exhibit frequency gaps. In the long-wavelength limit, the first band becomes linear,

(9) $$k_{1}\left(\omega \right)=\frac{\omega }{c\sqrt{\tilde{\varepsilon }}},\;\;\frac{1}{\tilde{\varepsilon }}=\frac{1}{T}\int_{0}^{T}\frac{dt}{\varepsilon (t)},$$

so that the effective permittivity is the harmonic-time average $1/\tilde{\varepsilon }$.

For a finite slab of thickness *L* embedded in time-invariant media, a monochromatic incidence at $\omega_{0}$ excites temporal harmonics $\omega_{0}-n\Omega$ inside and outside the slab. The fields are expanded over bulk Bloch modes $k_{p}\left(\omega_{0}\right)$ and their harmonic content, and amplitudes follow from boundary matching at y=0 and y=L. The formulation derives both instantaneous and time-averaged Poynting vectors for the transmitted and reflected fields, identifying nonoscillatory contributions to the energy flux and possible beat terms when $\omega_{0}=\frac{N}{2}\Omega$. These expressions quantify how modulation pumps energy into selected harmonics, permitting reflected and transmitted coefficients with magnitudes exceeding unity without violating energy conservation, since the modulation source supplies or extracts power.

Based on the above aspects, numerical examples for sinusoidal modulation,

(10) $$\varepsilon \left(t\right)=\varepsilon_{0}+\Delta \varepsilon \;\sin\left(\Omega t\right),$$

span weak, moderate, and strong regimes. In the weak limit, the fundamental response reproduces Fabry–Pérot behavior with $\varepsilon_{0}$, while higher harmonics are small. As Δε increases, harmonic strengths grow and can surpass the fundamental in selected bands. Specific harmonics vanish at $\omega_{0}/\Omega$ equal to positive integers up to the harmonic order, and their phases exhibit discontinuities at those points. The calculations consistently reflect the underlying bulk band picture with momentum gaps and the periodicity ω↦ω+Ω, and they clarify under which conditions the time averaged flux reduces to an incoherent sum over harmonics and when additional beat terms contribute. Overall, the work provides a self-contained bulk modal analysis combined with slab mode matching to predict polychromatic reflection and transmission from temporally periodic media, relates these spectra to a momentum gap band structure, and identifies effective medium and energy flow limits that are essential for device design.

**Figure 2:**
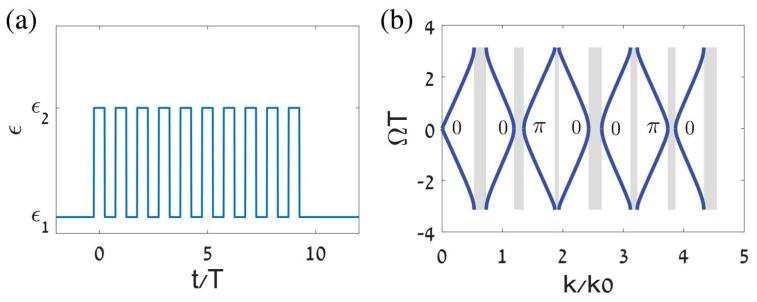
(**a**) Two-level temporal step changes of permittivity and (**b**) dispersion bands with momentum gaps [43].

Topological photonic time crystals were introduced in [43], demonstrating that periodic index jumps generate Floquet bands with momentum gaps and quantized Zak phases. Each abrupt jump produces time refraction and time reflection, and their interference over a period *T* forms Floquet–Bloch states whose quasifrequency follows a closed-form dispersion. For a binary time crystal with segments $\epsilon_{1},\epsilon_{2}$ of durations $t_{1},t_{2}$, continuity of the displacement and magnetic fields across the temporal boundaries yields a 2×2 period transfer matrix, and the Floquet condition leads to the following:

(11) $$\Omega \left(k\right)=\frac{1}{T}\cos^{-1}\;\left(W+X\right),$$

where *W* and *X* are matrix elements determined by $\epsilon_{1},\epsilon_{2},t_{1},t_{2}$ and the conserved wavenumber *k*. Real Ω identifies pass bands, while complex Ω identifies momentum gaps. Figure 2 shows the temporal lattice and the associated band structure in momentum and quasifrequency space [43], with each band labeled by its Zak phase 0 or π, clearly revealing the momentum gaps produced by temporal switching.

The band topology is quantified by a Zak phase for each band

(12) $$\theta_{\mathrm{Zak}}^{\left(m\right)}=\int_{-\pi /T}^{\pi /T}\;\;d\Omega \;i\int_{0}^{T}\;dt\;\epsilon \left(t\right)\;D_{m,\Omega }^{*}\left(t\right)\;\partial_{\Omega }D_{m,\Omega }\left(t\right),$$

which takes the values 0 or π and fixes measurable phase relations between the time-refracted and time-reflected components. For waves with *k* in a gap, the two outgoing components after the crystal have equal magnitude and a relative phase whose sign is set by the pattern of Zak phases across the lower bands. The same analysis predicts temporal topological edge states, since concatenating two time-crystals with different band topologies yields a pulse that peaks at the temporal interface and exponentially decays away from it on both sides in time. Replacing step jumps with smooth periodic ϵ(t) preserves the band topology since the resulting wave equation maintains Floquet symmetry and yields Zak phases that remain integer-valued, which is verified numerically. This establishes that time periodic media act as temporal analogs of photonic crystals with robust topological phase control and interface states in time, and points to experimental routes such as epsilon near-zero platforms for observing momentum gaps, controllable phase between conjugate components, and temporal edge localization.

**Figure 3:**
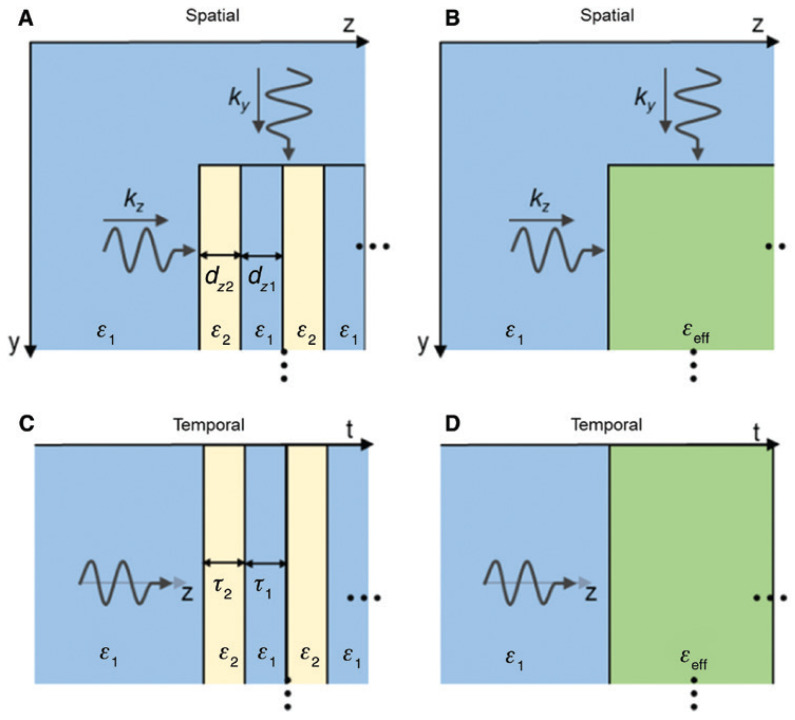
(**A**) Spatial multilayered representation with successive layers of media with $\varepsilon_{1}$, $\varepsilon_{2}$, (**B**) effective medium resulting from spatial multilayered representation, (**C**) temporal multistepped representation with successive time-layers of media with $\varepsilon_{1}$, $\varepsilon_{2}$, and (**D**) effective medium resulting from temporal multilayered representation [44].

Pacheco-Peña and Engheta, in [44], formalized a temporal counterpart of spatial multilayers by rapidly alternating the permittivity of a spatially uniform medium between two values, thus yielding a multistepped temporal metamaterial with a clearly defined effective response. The key kinematic constraint is the conservation of the wave vector at a temporal boundary, so the carrier frequency shifts according to

(13) $$\omega_{2}=\omega_{1}\sqrt{\frac{\varepsilon_{1}}{\varepsilon_{2}}},$$

while the wavelength is unchanged. Small jumps also generate a weak time-reflected (backward) component. Building on a transfer-matrix construction for successive time steps, the work derives a closed-form temporal effective-medium law,

(14) $$\varepsilon_{eff}=\frac{\varepsilon_{1}\varepsilon_{2}}{\varepsilon_{1}\Delta t_{2}+\varepsilon_{2}\Delta t_{1}},$$

with the harmonic mean of the step values weighted by their duty cycles $\Delta t_{1,2}$. This expression represents the time dual of the normal field effective permittivity in spatial multilayers and accurately predicts the evolution of the fundamental harmonic of the forward wave when the modulation period is much shorter than the optical period. Also, Ref. [44] quantifies amplitude “ripples” and higher harmonics arising from repeated temporal interfaces and show how duty-cycle control tunes $\varepsilon_{eff}$ across the interval between $\varepsilon_{1}$ and $\varepsilon_{2}$. The side-by-side comparison of spatial multilayers and temporal multisteps, as illustrated in Figure 3 [44], together with their effective media, clarifies that spatial stacking maps to time sequencing and that continuity of the normal component of electric displacement, D, in space translates to continuity across temporal steps.

**Figure 4:**
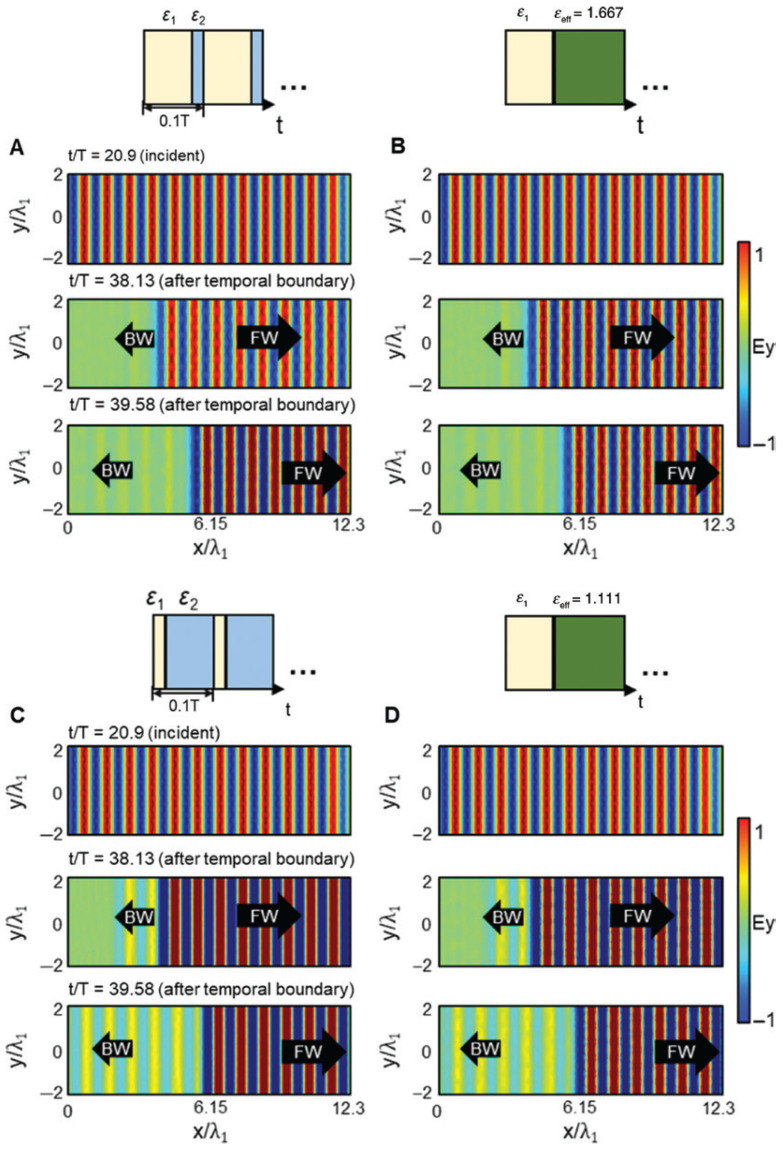
(**A**) Temporally periodic permittivity (from $\varepsilon_{1}=2$ to $\varepsilon_{2}=1$) with a periodicity of (1/10)T and duty cycle 0.2, (**B**) single-step function from $\varepsilon_{1}=2$ to $\varepsilon_{eff}=1.667$, (**C**) temporally periodic permittivity (from $\varepsilon_{1}=2$ to $\varepsilon_{2}=1$) with a periodicity of (1/10)T and duty cycle 0.8, and (**D**) single-step function from $\varepsilon_{1}=2$ to $\varepsilon_{eff}=1.111$ [44].

Time-domain simulations in COMSOL validate this theory across weak and strong contrast, including cases with large $\varepsilon_{2}$ where higher-order sidebands remain well below the fundamental, and they visualize both forward and backward waves after the switch, confirming the conserved wavelength and frequency conversion predicted by the boundary analysis. Snapshots of the field for two duty cycles show the forward wave at the new frequency and the backward replica with the same spatial period, with stronger back-conversion for smaller $\varepsilon_{eff}$. Figure 4 shows these space–time field maps and highlights how duty cycle controls the backward-wave amplitude [44]. These results establish a practical “temporal EMT” that enables programmable, dispersion-independent frequency translation and effective parameter design through time sequencing rather than spatial layering.

**Figure 5:**
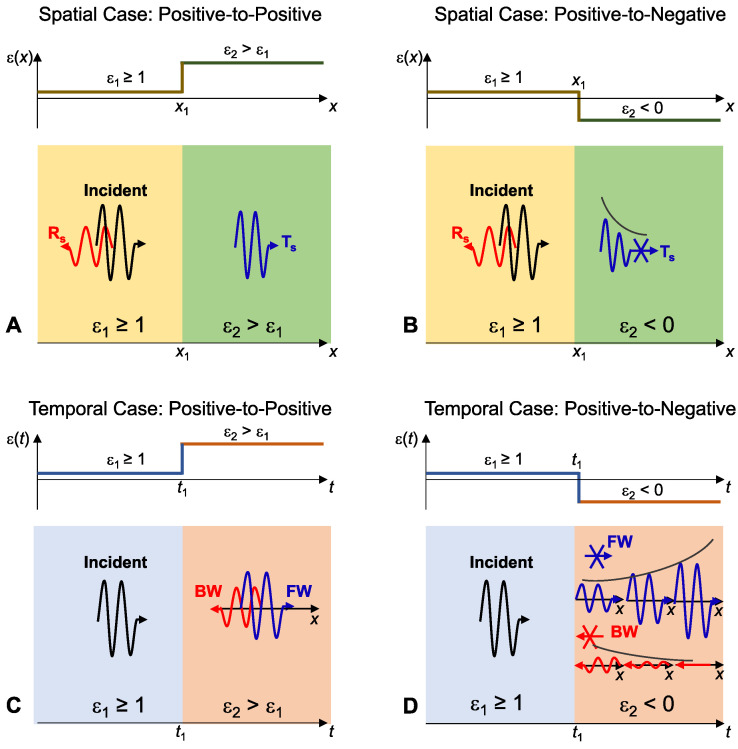
(**A**) Spatial boundary between two media with $\varepsilon_{2}>\varepsilon_{1}\geq 1$, (**B**) spatial boundary between two media with $\varepsilon_{1}\geq 1>0>\varepsilon_{2}$, (**C**) temporal boundary between two media with $\varepsilon_{2}>\varepsilon_{1}\geq 1$, and (**D**) temporal boundary between two media with $\varepsilon_{1}\geq 1>0>\varepsilon_{2}$ [27].

A distinct temporal mechanism capable of freezing and amplifying electromagnetic waves was proposed in [27], based on rapid transitions between positive and dispersionless negative permittivity. The key physics was analyzed for plane waves and Gaussian pulses in an unbounded, isotropic medium with ε(t), showing that a single positive-to-negative temporal transition “freezes” the spatial phase while the total field amplitude grows exponentially in time. Returning ε to a positive value “thaws” the wave and, if the final ε differs from the initial one, enacts frequency conversion. The analysis relates the feasibility of a dispersionless negative response to non-Foster realizations and describes energy and momentum conservation through the space averaged Poynting vector and the D×B momentum density. A compact expression captures the frozen-wave growth after a positive to negative jump at time $t_{1}$. The forward and backward components acquire purely exponential time factors with rate $\left(\sqrt{\varepsilon_{1}/\left|\varepsilon_{2}\right|}\right)\omega_{1}$ while the wavenumber remains $k_{1}$ and the post-jump frequency is imaginary, for example

(15) $$E_{2,t_{1}}=\frac{1}{2}\left(1+\frac{\varepsilon_{1}}{|\varepsilon_{2}|}\right)E_{1}\;e^{\;\omega_{1}\sqrt{\varepsilon_{1}/\left|\varepsilon_{2}\right|}\;\left(t-t_{1}\right)}\;+\;\frac{1}{2}\left(1-\frac{\varepsilon_{1}}{|\varepsilon_{2}|}\right)E_{1}\;e^{-\omega_{1}\sqrt{\varepsilon_{1}/\left|\varepsilon_{2}\right|}\;\left(t-t_{1}\right)},$$

with one branch growing and the other decaying in time. This behavior is derived by enforcing temporal boundary conditions that conserve D and B at the jump, yielding closed-form forward and backward fields. Figure 5 illustrates the result [27] within the broader space–time analogy by contrasting spatial and temporal interfaces, including the positive-to-negative temporal boundary that freezes the phase and drives exponential growth, thereby motivating the non-Foster implementation.

**Figure 6:**
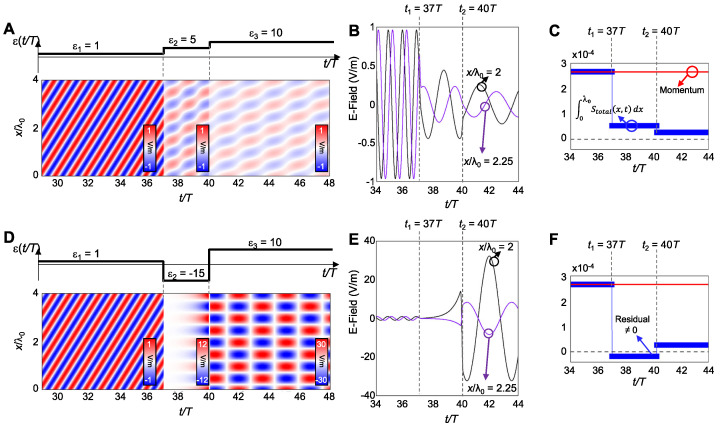
(**A**) Electric field distribution when $\varepsilon_{1}=1\to \varepsilon_{2}=5\to \varepsilon_{3}=10$, (**B**) electric field distribution at $x_{1}=2\;\lambda_{0}$ and $x_{2}=2.25\;\lambda_{0}$, (**C**) average of instantaneous Poynting vector over $\lambda_{0}$ and momentum as a time function, (**D**) electric field distribution when $\varepsilon_{1}=1\to \varepsilon_{2}=\;$-$15\to \varepsilon_{3}=10$, (**E**) electric field distribution at $x_{1}=2\;\lambda_{0}$ and $x_{2}=2.25\;\lambda_{0}$, and (**F**) average of instantaneous Poynting vector over $\lambda_{0}$ and momentum as a time function [27].

The study then examines a temporal slab in which ε undergoes two rapid steps, namely, positive-to-negative at $t_{1}$, then negative-to-positive at $t_{2}$. During $t_{1}<t<t_{2}$, the wave is spatially frozen and its total field grows exponentially. At $t>t_{2}$, the wave resumes propagation with frequency $\omega_{3}=\sqrt{\varepsilon_{1}/\varepsilon_{3}}\omega_{1}$, and the superposition of the thawed forward and backward components forms a near-standing pattern with a small net Poynting flux that preserves momentum. The analysis provides a closed-form expression for the field after the slab, which makes the dependence on the dwell time $t_{2}-t_{1}$ fully explicit, showing how the exponentially growing and decaying contributions inside the slab determine the amplitudes of the thawed forward and backward waves after $t_{2}$. This mapping enables controlled amplification and frequency conversion when one selects $\varepsilon_{2}<0$, $\varepsilon_{3}>0$, and an appropriate slab duration. Figure 6 integrates these ideas by showing the space–time evolution for a {positive→negative→positive} sequence [27], where the incident wave freezes and grows between the two temporal boundaries and then thaws to a higher amplitude and a new frequency once ε is restored to a positive value.

To ground the concept, Ref. [27] simulates a parallel-plate waveguide loaded with switchable, time-dependent layers that emulate non-Foster negative capacitors. Upon activation, the guided wave is held and amplified within the loaded section in agreement with the unbounded medium theory, and related dipole radiation scenarios exhibit the same freeze and grow dynamics. The overall narrative establishes a temporally local, non-Foster route to stopping and boosting waves, complementary to photonic time crystals, while preserving momentum and offering deterministic frequency translation when the wave is released.

The works summarized in this subsection establish the core physical principles and theoretical frameworks governing time-varying permittivity. These foundations include the conservation of wavenumber at temporal boundaries, Mathieu stability and instability tongues in periodically modulated media, WKB evolution in slowly varying permittivity profiles, temporal Fresnel laws, Floquet band formation and momentum gaps in photonic time crystals, temporal effective-medium limits, and non-Foster transitions that enable wave freezing and amplification. These principles provide the foundation for the more general cases of ε-μ co-modulation, time-varying conductivity, and temporally evolving plasmas discussed in the following subsections.

#### 3.1.2. Time-Varying Co-Modulation of Permittivity and Permeability

When both permittivity and permeability vary in time [45,46,47,48], new regimes of wave control emerge that cannot be accessed through permittivity modulation alone. Joint variation in ε(t) and μ(t) alters the wave speed, impedance, stored energy, and coupling between temporal harmonics, and in certain coordinated scenarios, the medium becomes effectively reflectionless despite rapid modulation. Co-modulation also links temporal media to synthetic motion analogies, enabling effects that are traditionally associated with moving dielectrics, such as Fresnel drag- and direction-dependent dispersion, without physically transporting matter. The works presented here establish the foundational rules, radiation behavior, Floquet band structure, and effective-medium picture governing media with simultaneously time-varying electric and magnetic responses.

A central kinematic scheme arises from the classic analysis of velocity-modulated dielectrics. Morgenthaler, in [17], showed that when ε(t) and μ(t) vary in time while keeping their ratio μ(t)/ε(t) constant, the medium maintains constant impedance and therefore supports reflectionless propagation even under abrupt parameter changes. For a TEM plane wave, the phase velocity

(16) $$v\left(t\right)=\frac{1}{\sqrt{\mu (t)\varepsilon (t)}},$$

fully determines the instantaneous frequency through ω(t)=βv(t), while the wavenumber β remains fixed. This conservation law follows from the temporal boundary conditions enforcing continuity of the dielectric current density, D, and magnetic field flux, B, across a velocity step, which ensures that the wavelength is unchanged even though the carrier frequency shifts. The analysis further highlights that energy density in the field generally changes when the medium jumps between $\left(\varepsilon_{1},\mu_{1}\right)$ and $\left(\varepsilon_{2},\mu_{2}\right)$, whereas the electromagnetic momentum density

(17) $$g=\frac{S}{v^{2}}$$

remains invariant, which is evidence that the modulation source performs work on the field without altering its momentum.

The second half of [17] develops a transit time picture for a finite slab of length *L* whose velocity v(t) is modulated while a monochromatic wave traverses it. The accumulated phase at the exit,

(18) $$\phi \left(t\right)=\omega_{c}\left[t-\tau \left(t\right)\right],$$

is governed by the time-varying transit time τ(t), so the output frequency becomes

(19) $$\omega_{out}\left(t\right)=\omega_{c}\left(1-\frac{d\tau }{dt}\right).$$

For thin slabs with sinusoidal modulation, $\varepsilon \left(t\right)=\varepsilon_{0}\left[1+b\sin\left(\Omega t\right)\right]$, the theory predicts nearly linear phase modulation and small fractional frequency deviations proportional to $bL\Omega /v_{0}$. Electrically long slabs require a more general expression for the transit time, from which design guidelines for maximizing modulation-driven frequency shifts are obtained. Estimates using practical ferroelectric materials indicate that velocity modulation offers frequency control over microwave carriers without relying on resonant structures. Overall, the work establishes a canonical description of reflectionless co-modulation with constant impedance and provides quantitative tools for engineering temporal phase modulators based on velocity variation.

A complementary perspective arises from the radiation theory developed in [49], which examines arbitrary electromagnetic sources embedded in a homogeneous but time-varying medium. The study contrasts two regimes, namely synchronous co-modulation, in which both parameters scale as

(20) $$\varepsilon \left(t\right)=\varepsilon_{0}\alpha \left(t\right),\;\;\mu \left(t\right)=\mu_{0}\alpha \left(t\right),$$

and impedance-varying modulation, in which ε(t) and μ(t) evolve independently. In the constant-impedance regime, Maxwell’s equations can be mapped to their vacuum form by redefining the time coordinate, so the radiation fields follow conventional retarded integrals modified by an effective temporal scaling. Depending on the choice of α(t), linear, exponential, or engineered profiles, the radiated spectrum experiences systematic redshifts or blueshifts, and temporal features stretch or compress in a manner reminiscent of cosmological expansion. Remarkably, it is possible to suppress radiation from a given source or force a nominally dc source to radiate continuously, purely through the imposed time dependence.

In the impedance-mismatched regime, Ref. [49] offers exact solutions for a tractable class of permittivity profiles, revealing additional behaviors that are unavailable in co-modulation. Time variation in ε(t) alone generates spectral modulation accompanied by causal “echoes” in the detected field and produces longitudinal components that propagate into the far zone, effectively enlarging the apparent source region in space as the interaction unfolds. The results show how synchronized versus independent temporal drives determine whether the medium acts as a passive frequency shifter, a parametric amplifier or suppressor of radiation, or a generator of temporally structured field components that persist well beyond the source.

On the other hand, periodic temporal co-modulation introduces further structure, enabling temporal photonic crystals with joint electric and magnetic drives. Martínez-Romero et al., in [50], constructed a systematic Floquet algorithm for media whose permittivity and permeability oscillate as

(21) $$\varepsilon \left(t\right)=\varepsilon_{0}\left[1+m_{\varepsilon }\cos\left(\Omega t\right)\right],\;\;\mu \left(t\right)=\mu_{0}\left[1+m_{\mu }\cos\left(\Omega t+\theta \right)\right],$$

with amplitudes $m_{\varepsilon },m_{\mu }$ and relative phase θ. A temporal Floquet expansion converts Maxwell’s equations into an infinite coupled system for the harmonic coefficients. Truncation in the weak-modulation limit yields approximate expressions for the band structure, including the opening of momentum gaps near ω≈Ω/2. A key finding is that equal, in-phase co-modulation ($m_{\varepsilon }=m_{\mu }$, θ=0) preserves the wave impedance and thus closes the *k*-gap, so the medium behaves as a velocity-modulated system with constant impedance and no Bragg scattering between Floquet components. Conversely, unequal modulation amplitudes or a non-zero phase difference open finite momentum gaps, producing strong parametric coupling between frequency channels separated by Ω.

Numerical solutions of the full Floquet matrix reveal an infinite ladder of temporal bands with periodicity Ω in frequency and a rich dependence of gap widths on the electric and magnetic modulation balance. Lossy extensions show that complex ε and μ dampen but do not suppress the momentum gaps, while attenuation competes directly with amplification generated by the modulation. Transmission calculations for finite temporal–crystal slabs show that weak modulation yields Fabry–Pérot-like responses with small sidebands, while stronger modulation leads to parametric resonances and transmitted amplitudes exceeding unity, reflecting net energy transfer from the modulation to the wave. In fact, Ref. [50] generalizes temporal crystals to the full electromagnetic parameter space and clarifies how cooperative or antagonistic electric and magnetic drives shape momentum gaps and the resulting time-domain scattering spectra.

A spatiotemporal extension of co-modulation links time-varying media to synthetic motion. Hence, Huidobro et al., in [51], developed a homogenized description of traveling-wave modulations of both permittivity and permeability,

(22) $$\varepsilon \left(x,t\right)=\varepsilon_{m}\left[1+2\alpha_{e}\cos\left(gx-\Omega t\right)\right],\;\mu \left(x,t\right)=\mu_{m}\left[1+2\alpha_{m}\cos\left(gx-\Omega t\right)\right],$$

and showed that, in the long-wavelength and small-modulation limit, such a medium behaves as an effectively moving dielectric. The homogenized structure is equivalent to a uniaxial bianisotropic material whose magnetoelectric coupling ξ induces an effective drift velocity

(23) $$\frac{v_{D}}{c_{m}}=\frac{2\xi }{\varepsilon +\mu },\;c_{m}=\frac{1}{\sqrt{\varepsilon_{m}\mu_{m}}},$$

so that properly phased co-modulation produces Fresnel drag and nonreciprocal dispersion without any physical motion.

Within this effective moving medium approach, the dispersion relation acquires a Doppler tilt in the (ω,k)-plane, and the band gaps become asymmetric, so that forward and backward waves exhibit different phase and group velocities. By tuning the relative phase between ε(t) and μ(t), one can control both the sign and magnitude of $v_{D}$, including regimes where the drag reverses direction even though the modulation travels in a fixed direction. The model predicts stability limits, as $|v_{D}|$ approaches the luminal boundary $c_{m}$, the effective medium exhibits gain and band-merging, whereas subluminal operation yields passive, stable Fresnel drag. The authors also connect the homogenized model to concrete realizations using modulated metamaterial unit cells or transmission line platforms.

These works delineated the fundamental mechanisms that govern media in which ε(t) and μ(t) evolve in time. When co-modulation preserves constant impedance, propagation remains reflectionless and frequency shaping becomes possible through controlled velocity variation. When the two parameters vary independently, the medium acquires richer behavior that includes parametric radiation effects, temporal echoes, and longitudinal field components that reshape the apparent source region. Periodic electric and magnetic drives create temporal photonic crystals with momentum gaps and parametric resonances whose strength depends on the balance between the two modulations. Spatiotemporal co-modulation extends these ideas further by generating synthetic motion and Fresnel drag, offering a flexible route to broadband nonreciprocity without relying on mechanical movement or magneto-optic bias.

#### 3.1.3. Time-Varying Conductivity

The temporal modulation of electrical conductivity [52,53] represents a distinct avenue for manipulating electromagnetic waves, with relevance to both natural environments and engineered platforms. Variations in volumetric conductivity arise in scenarios such as ionized media, atmospheric disturbances, or transients driven by nuclear processes, while controlled modulation of surface conductivity occurs in tunable plasmonic materials including graphene. Changes in σ(t) introduce dissipation, modify boundary conditions, and couple frequency components through temporal mixing. The works reviewed here develop the analytical foundations for wave interaction with time-varying conductive media and illustrate how both bulk and surface modulation enable frequency conversion, sideband synthesis, and ultrafast reconfigurability.

An analytical framework for bulk time-varying conductivity was established by Harfoush and Taflove [54], who examined the scattering of a normally incident plane wave from a half-space whose conductivity varies periodically in time. Modeling the normalized electric field as

(24) $${\left(\frac{E_{z}}{E_{0}}\right)}_{xx}={\left(\frac{E_{z}}{E_{0}}\right)}_{tt}+\frac{\sigma_{0}}{\epsilon_{0}\omega_{i}}{\left(f\;\frac{E_{z}}{E_{0}}\right)}_{t},$$

with dimensionless coordinates $x=kx^{\prime }$ and $t=\omega_{i}t^{\prime }$, the analysis explores both weak and strong modulation regimes. In the weak-perturbation case $\sigma \left(t\right)=\sigma_{0}\left[1+\epsilon \sin\left(vt+\psi \right)\right]$ with ϵ≪1, an asymptotic solution yields closed-form expressions for the scattered fields and the associated spectral sidebands. At the opposite limit, where $\sigma_{0}\to \infty$ and the modulation is represented as $\sigma \left(t\right)=\sigma_{0}/\left[1+\epsilon \sin\left(vt+\psi \right)\right]$, the study distinguishes a boundary layer region confined to a few skin depths from a deeper penetration regime. Within the boundary layer, tractable formulas describe the amplitudes and phases of scattered sidebands, while deeper inside the medium, a ray-based treatment captures the short time evolution governed by a parabolic equation.

Across all regimes, the analysis shows that periodic conductivity produces Floquet mixing, generating sidebands at $\omega_{i}+m\omega_{\sigma }$ whose amplitudes follow predictable trends with modulation depth and rate. A pronounced enhancement occurs when $\omega_{\sigma }$ approaches $2\omega_{i}$, aligning with classical conditions for maximal parametric interaction. Space–time ray trajectories within the half-space reveal modulated propagation paths that do not intersect, indicating the absence of temporal focusing. The analytical solutions exhibit close quantitative agreement with finite-difference time-domain (FDTD) simulations for both spectral content and field magnitudes, thereby establishing a reliable theoretical foundation for temporal scattering induced by conductivity modulation.

A complementary line of work concerns engineered surfaces with time-dependent sheet conductivity. Specifically, Salary et al. [55] investigated graphene-wrapped dielectric microwires whose graphene layer is electrically modulated to convert an incident frequency $\omega_{0}$ into sidebands $\omega_{0}\pm p\omega_{m}$ in the terahertz regime. The study develops a semianalytical transition-matrix method that treats a time-varying boundary as a temporal convolution, producing block matrices that couple cylindrical multipoles across temporal harmonics for both TE and TM polarization. The formulation naturally extends to clustered scatterers, periodic arrays, and substrate supported configurations through translation operators, lattice sum techniques, and Weyl type representations. Graphene is modeled using a Drude type intraband conductivity with electrically controlled Fermi energy, enabling gigahertz rate modulation.

Validation against FDTD simulations for isolated and periodic microwires (with radii around 40–50 μm and dielectric constants near 3.9) demonstrates excellent agreement, with average errors of only a few percent across relevant harmonics. The semi-analytical solver is computationally much lighter than full-wave time-domain methods, making it suitable for large space–time metasurface designs. The results highlight several functionalities enabled by conductivity modulation, including enhanced frequency conversion in single microwires, independent control of amplitude and phase at selected sidebands through the modulation parameters, holographic synthesis of higher-order temporal harmonics, and subwavelength effective-medium implementations in which the time-dependent Fermi energy maps directly onto an engineered refractive-index profile. These capabilities culminate in a proof-of-concept time-modulated metalens that performs ultrafast beam steering through dynamic index gradients.

The prior studies described the principal physical wave mechanisms associated with time-varying conductivity. The periodic modulation of bulk conductivity induces Floquet mixing, boundary layer behavior, and predictable sideband structures validated by numerical simulation. The temporal modulation of surface conductivity, particularly in graphene-based platforms, enables compact and efficient frequency conversion, programmable sideband control, and ultrafast beam manipulation. The combination of analytical rigor and practical device demonstrations positions time-varying conductivity as a versatile tool within the broader landscape of temporal metamaterials.

#### 3.1.4. Time-Varying Plasma

Temporal changes in plasma density provide another important mechanism for shaping electromagnetic wave propagation [56,57]. Unlike permittivity or conductivity modulation in ordinary dielectrics, variations in the plasma frequency introduce strong dispersion and can dramatically reshape both the spectral and temporal signatures of fields that are already present when the change occurs. These effects arise in natural environments, such as ionospheric disturbances or rapid ionization events, and in engineered scenarios involving ultrafast plasma generation. The works reviewed here establish the fundamental behavior of waves interacting with plasmas whose constitutive parameters switch abruptly in time and clarify how temporal and spatial discontinuities combine to produce characteristic scattering features.

A foundational analysis of plasma creation in the presence of pre-existing fields was developed in [58], which examines the instantaneous formation of a uniform, cold, lossless plasma at time t=0 within a region supporting arbitrary confined sources. Using Laplace transforms and retarded and auxiliary Green dyadics, the study derives closed-form expressions for the resulting time-domain fields and then specializes these formulas to plane-wave propagation and dipole radiation. For a monochromatic incident wave at frequency $\omega_{0}$, the temporal jump produces two counter-propagating components with a common frequency

(25) $$\omega =\sqrt{\omega_{0}^{2}+\omega_{p}^{2}},\;\;\alpha \equiv \frac{\omega_{p}}{\omega_{0}},$$

and analytical expressions show how amplitude, energy, and power partition between these components. The net forward power flux decreases to

(26) $$S_{\mathrm{net}}=\frac{S_{0}}{1+\alpha^{2}},$$

and the group velocity of each branch diminishes as the plasma frequency increases, approaching zero in the overdense limit. These features reflect the work performed on the field by the sudden increase in free electron density and produce direct pulse-level effects, including temporal splitting and stretching or compression, governed by the spectral content of the incident waveform.

The radiation of a dipole operating before the creation event exhibits an equally rich structure. Once the plasma forms, the radiated field separates into spherical waves propagating inward and outward, each oscillating at the shifted frequency ω. Jiang provides closed-form expressions in terms of Lommel functions, and asymptotic analysis reveals several robust behaviors. The instantaneous radiated power displays a transient dip due to energy drawn into establishing the plasma response. The subsequent buildup phase features beating at the plasma frequency and a spatially varying magnetic term that would be suppressed by even modest loss. When the plasma is overdense, spatial attenuation becomes exponential, leading to rapid decay of the dipole field. These results provide a complete temporal-interface description of plasma onset in the presence of sources and connect spectral shifts, energy balance, and transient dynamics in a transparent manner.

A complementary perspective is offered in [59], which investigates the reflection of a normally incident plane wave when a semi-infinite cold plasma is suddenly created in the region z>0. This configuration combines a temporal change with a spatial boundary, and the medium is inherently dispersive. A transmission line analogy clarifies that two distinct reflected components arise. Scattering at the spatial boundary conserves the angular frequency and produces an “*A*-wave” at the original frequency with reflection coefficient $R_{s}=\left(\eta_{p}-\eta_{0}\right)/\left(\eta_{p}+\eta_{0}\right)$. Scattering at the temporal boundary conserves the wavenumber and generates a “*B*-wave” inside the plasma at a new frequency $\omega_{1}={\left(\omega^{2}+\omega_{p}^{2}\right)}^{1/2}$, which then propagates toward the interface and is partially transmitted into free space. The amplitude of this *B* component is strongly influenced by collisions. For a plasma with collision frequency $\nu \ll \omega_{p}$, its attenuation constant is approximately

(27) $$\alpha \approx \frac{\nu \;\omega_{p}^{2}}{2\;\omega \;\omega_{1}\;c^{2}},$$

so that the observable *B*-wave contribution decays as $R_{B}∼R_{t}t_{p1}e^{-\alpha z_{1}}$, where $z_{1}$ is the distance between the creation point and the interface. Even moderate loss therefore suppresses the temporal refraction component in the steady state, so the spatially reflected *A* wave becomes dominant. Parametric studies show that $R_{B}$ peaks near $Q=\omega /\omega_{p}\approx 0.5$ and declines rapidly thereafter, whereas $R_{s}$ decreases for Q>1. Using Laplace transforms, the study obtains closed-form expressions that reduceover long periods of time to the expected *A* and *B* contributions and confirm the transmission line interpretation. The results provide a complete transient solution that reconciles the effects of both spatial and temporal scattering and identifies the conditions under which the temporal refraction signature is appreciable.

The above works determined the essential physics governing time-varying plasmas. Abrupt creation of a plasma induces frequency upshifts, temporal splitting, and redistribution of energy between counter-propagating components. Radiation sources experience transient suppression and oscillatory buildup, accompanied by new spectral features tied to the plasma frequency. When temporal and spatial boundaries coincide, the reflected field separates into contributions associated with each mechanism, with collisions determining whether the temporal component is observable. These analytical results form a foundation for understanding ultrafast ionization events, plasma-based switching, and temporally programmed dispersive media.

### 3.2. Space–Time-Varying Media

Simon, in [60], developed one of the earliest systematic treatments of electromagnetic mixing in media whose permittivity varies jointly in space and time through a progressive modulation of the form $\varepsilon \left(z,t\right)=\varepsilon_{0}+\Delta \varepsilon \cos\left(\omega_{1}t-k_{1}z\right)$. Starting from Maxwell’s equations and expanding the guided fields in Fourier–Floquet harmonics, the analysis reduces to an infinite but structured linear system for the coefficients $\alpha_{n}$. Retaining the dominant terms n∈{−1,0,+1} isolates the essential Bragg triad responsible for strong coupling. This perturbative closure exposes the conditions under which a guided mode at frequency ω exchanges power with its temporal sidebands at $\omega \pm \omega_{1}$.

When the Bragg phase velocity is matched, the up-converted reflected wave at $\omega +\omega_{1}$ satisfies the power relation

(28) $$\frac{P_{\omega +\omega_{1}}}{P_{\omega }}=\frac{\omega +\omega_{1}}{\omega },$$

which expresses a Manley–Rowe-type photon flux conservation and clarifies that the additional power is supplied by the traveling modulation. Physically, this relation reflects energy exchange with the traveling pump, as the modulation injects or extracts quanta at $\omega_{1}$, causing the converted sideband power to scale with the frequency shift. At the same time, the coupling selectivity remains governed by the spacetime phase $\omega_{1}t-k_{1}z$, which enforces simultaneous frequency and momentum matching. Down-conversion follows the same logic and predicts either reduced output or parametric amplification, depending on the sign of $\omega_{1}-\omega$. The perturbative model identifies threshold conditions under which the three-wave system supports exponentially growing solutions, linking the behavior of traveling-wave parametric structures to the mechanism familiar from backward-wave oscillators and cavity parametric amplifiers. Simon’s formulation therefore provides a foundational propagation equation together with physically transparent power balances that serve as a reference for spatiotemporal mixing in guided media.

**Figure 7:**
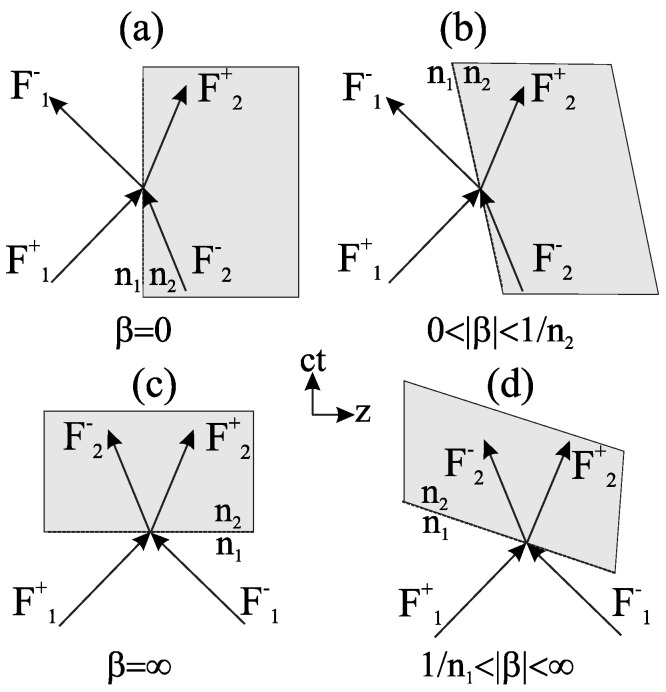
(**a**) Space-like interface with β=0, θ=0 (static), (**b**) interface with β<0, $0<|\beta |<1/n_{2}$, (**c**) time-like interface with β±∞, θ±π/2, and (**d**) interface with β<0, $1/n_{1}<\left|\beta \right|<\infty$ [61].

A rigorous alternative viewpoint was introduced in [61], which derives a forward and backward Maxwell formalism for arbitrary one dimensional index profiles n(z,t) without invoking envelope approximations. By casting the wave equation in rotated (p,q) coordinates, the analysis identifies which quantity is conserved at an interface of a given tilt in the (z,ct) plane and obtains closed-form transfer matrices for nonstationary boundaries. This geometric structure is summarized in Figure 7 [61], which depicts the four canonical interface types and their preserved quantities and provides an immediate link between interface tilt and scattering behavior.

Three representative applications highlight the utility of the technique. First, for tilted and purely temporal boundaries, analytic expressions for reflection and transmission follow directly from the interface matrices and are corroborated by numerical simulations. Second, concatenating interface and propagation matrices yields generalized spatiotemporal Bragg reflectors whose band structure evolves smoothly from spatial stop bands to temporal momentum gaps as the interface tilt varies. Third, spatiotemporal lensing emerges naturally when a rectangular n(z,t) region rescales both temporal and spatial coordinates, producing pulse compression, broadening, and simultaneous shifts in frequency and wavenumber. The work demonstrates that a time-only slowly varying envelope approximation (SVEA) incurs diffraction errors near sharp modulations, underscoring the necessity of the full forward and backward formulation for accurate modeling.

Further extending the mechanistic picture, Huidobro et al., in [62], constructed a homogenization theory for media modulated by traveling waves ε(x−vt) and μ(x−ut). Transforming to the co-moving frame renders the modulation static, enabling direct averaging and revealing that, whenever both permittivity and permeability vary, the long-wavelength medium acquires bianisotropy and strong nonreciprocity, even at zero frequency. Only modulating a single parameter leaves the effective response reciprocal. The resulting magnetoelectric coupling in the laboratory frame,

(29) $$\xi_{\mathrm{eff}}=-\frac{v\;\varepsilon_{⊥}^{\prime }\mu_{⊥}^{\prime }+\left(1-v\;\xi^{\prime }\right)\;\xi^{\prime }}{{\left(1-v\;\xi^{\prime }\right)}^{2}-v^{2}\varepsilon_{⊥}^{\prime }\mu_{⊥}^{\prime }},$$

quantifies how the modulation speed and the relative phase of the ε/μ variations tune nonreciprocity. This effective coupling term provides a compact physical interpretation of space–time modulation as a form of synthetic motion. In this picture, the traveling modulation biases the medium so that waves experience an effective magnetoelectric (bianisotropic) response, which constitutes the fundamental ingredient enabling asymmetric scattering and nonreciprocal behavior in otherwise unbiased structures. Closed-form expressions for the effective dispersion of s and p polarizations identify the associated group velocities and provide immediate design rules for directional wave transport. The model is worked out for traveling bilayers and sinusoidal gratings, revealing that out-of-phase electric and magnetic modulations produce giant magnetoelectric coupling and that the entire structure maps exactly to an equivalent moving medium, except in the luminal regime, where amplification and breakdown of homogenization occur. This casts traveling-wave metamaterials as a compact and analytically controlled platform for biasing nonreciprocity, bandwidth, and stability.

Spatiotemporal modulation reaches its most intricate form in [63], which analyzes photonic structures that are periodic simultaneously in space and time. For separable profiles of the form $\varepsilon \left(z,t\right)=\varepsilon_{0}\varepsilon_{z}\left(z\right)\varepsilon_{t}\left(t\right)$, the field separates into spatial Bloch modes and temporal Floquet modes. The resulting band structure is indexed by paired eigenvalues $\left(K_{B},\Omega_{F}\right)$, each potentially complex, giving rise to three distinct types of spectral gaps. Spatial periodicity produces energy gaps associated with complex $K_{B}$, temporal periodicity produces momentum gaps associated with complex $\Omega_{F}$, and the overlap of the two opens mixed gaps in energy and momentum. A key discovery is that, in mixed gaps, the exponential growth driven by temporal modulation and the exponential decay due to spatial periodicity can exactly counterbalance. Under this condition, the gap collapses to an exceptional point where the modes grow linearly rather than exponentially, a behavior traced to non-Hermitian degeneracies in the joint spectrum.

Time-domain simulations support these predictions. Pulses tuned to energy gaps localize, pulses tuned to momentum gaps exhibit temporal amplification with minimal translation, and pulses tuned to mixed gaps show hybrid behavior governed by the balance between $ℑ\{\Omega_{F}\}$ and $ℑ\{K_{B}\}$. At the exceptional point, the field broadens linearly in both space and time while radiating backward and accumulating linearly increasing energy and signed momentum. The analysis provides a clear recipe for constructing and interpreting spatiotemporal band structures and for choosing operating points to obtain amplification, localization, or controlled radiation.

These studies formulated a unified view of spatiotemporal media spanning guided wave geometries, phenomena driven by interfaces, homogenized traveling wave structures, and fully periodic crystals. They reveal how joint variation in space and time mediates power exchange, opens and closes spectral gaps, induces nonreciprocity, and enables behaviors that neither spatial nor temporal modulation alone can achieve.

### 3.3. Time-Varying Circuits and Meta-Atoms

Time-varying circuit platforms [64] and temporally modulated meta-atoms [65,66] form an essential bridge between the theoretical constructs discussed earlier and the realizable building blocks of practical nonstationary metamaterials. Transmission lines whose reactive elements evolve in time provide circuit-level analogs of spatiotemporal media, while deeply subwavelength inclusions with prescribed temporal modulations act as unit cells capable of steering energy, generating sidebands, or storing and releasing electromagnetic power in ways unavailable to static structures. The works summarized here establish the foundations of this emerging hardware landscape and clarify how temporal control maps into frequency conversion, nonreciprocal transport, parametric radiation, and programmable scattering at both distributed and local scales.

**Figure 8:**
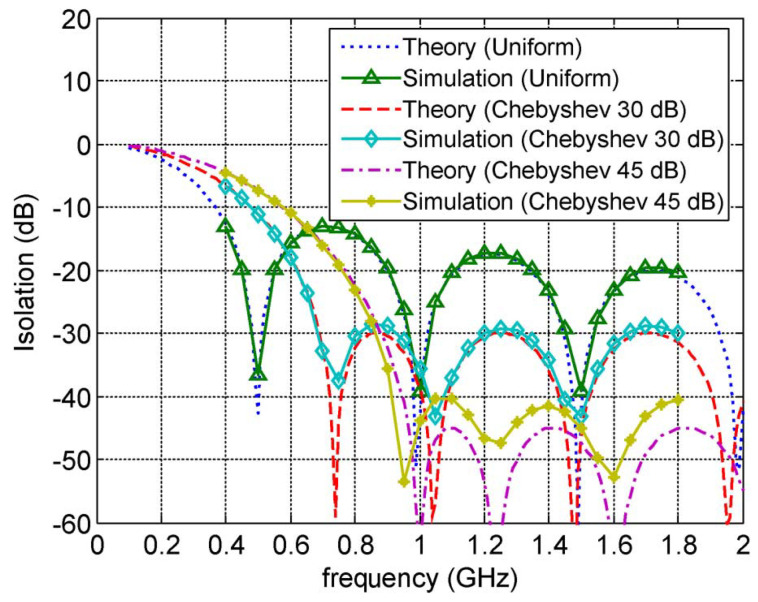
TX and RX isolation using uniform and nonuniform capacitance modulations in DMC [67].

A representative starting point is provided in [67], which introduces distributedly modulated capacitors (DMCs) as a practical means to achieve broadband, magnet-free nonreciprocity in transmission lines. A traveling pump modulates the per unit length capacitance as $C\left(z,t\right)=C_{0}+\Delta C\cos\left(\Omega t-k_{m}z\right)$, so that waves propagating in the same direction as the pump experience parametric frequency conversion into upper and lower sidebands, while waves propagating in the opposite direction remain largely unaffected. The resulting separation of transmit and receive signals at a single physical port rests on two complementary viewpoints. The first follows modified Telegrapher equations and provides closed-form expressions for the carrier and first-order sidebands. The second treats each unit cell loaded with varactors as a small capacitive mixer whose contributions add coherently in one direction because the modulation supplies momentum bias. Energy flow follows the Manley–Rowe relations, and a key design insight is that the isolation spectrum is the Fourier transform of the longitudinal modulation profile. For an array of *N* uniformly weighted varactors, the isolation versus frequency offset follows a discrete aperture sinc envelope, while tapered weighting suppresses sidelobes at the cost of broadening the main lobe. Figure 8 illustrates this trade-off for uniform and Dolph–Chebyshev tapering [67]. Measurements from a double-balanced microstrip prototype confirm isolation above 30 dB near 500 MHz, low insertion loss, conversion gain in line with the theory, and a noise figure near 1 dB, collectively demonstrating that DMCs enable IC-compatible, broadband circulators in which nonreciprocity arises entirely through time modulation.

A complementary route to temporal electromagnetic crystals was established in [68]. The proposed dynamic transmission line (DTL), implemented as a ladder network with junction capacitances modulated periodically in time, satisfies a nodal voltage equation that is directly equivalent to the wave equation of a dispersionless dielectric with time-periodic permittivity. In the long-wavelength limit, the correspondence $\tilde{C}\left[V_{X}\left(t\right)\right]/a=\varepsilon \left(t\right),\;L/a=\mu ,\;\left(\beta a\right)/2\ll 1$ connects the DTL to a temporal photonic crystal. A Bloch–Floquet expansion then yields a secular equation for ω(β) that predicts a true *k*-gap once the modulation depth surpasses a threshold set by losses. The authors fabricate an eight-cell microstrip prototype and retrieve the band structure experimentally, observing a clear β-gap in quantitative agreement with full models and effective-medium predictions. They track how the gap widens with increasing modulation depth, identify a threshold set by dissipation, and show that Δβ grows approximately as $\sqrt{M^{2}-M_{R}^{2}}$ once the threshold is exceeded. The study establishes that a DTL behaves as a temporal photonic crystal with a momentum gap, enabling circuit-level demonstrations of wave vector-selective transport and temporal crystal phenomena.

Moving from distributed circuits to temporal meta-atoms, Ptitcyn et al., in [69], developed a time-domain model for scatterers whose reactive elements vary deliberately in time. Their formulation begins from the instantaneous power balance of a dipolar radiator driven by an external field and through the prescribed modulation of a load reactance. They demonstrate that frequency-domain treatments overlook essential temporal power terms, whereas an exact time-domain formulation yields closed expressions for radiated, reactive, supplied, and dissipated power. A central analytical result is the instantaneous radiated power,

(30) $$P_{\mathrm{rad}}\left(t\right)=-\;\frac{\mu_{0}}{6\pi c}\;\dot{p}\left(t\right)\cdot \dddot{p}\left(t\right),$$

which ensures energy conservation at every instant through $P_{in}+P_{reac}+P_{rad}+P_{diss}=0$. Full-wave simulations confirm agreement between the model and electromagnetic fields in both transmitting and receiving configurations. Building on these foundations, Reference [69] demonstrates two meta-atom functions that rely on temporal control. In the first, a time-varying inductance programs the induced current to grow linearly, storing energy in the near field without radiation and releasing it later as a short, high-amplitude pulse once the modulation ceases. In the second, modulating a capacitive load yields controlled frequency translation of the scattered field, enabling programmable spectral shifts at the unit cell level. These examples reveal that temporal modulation at deeply subwavelength scales enables energy accumulation, tailored pulse release, and frequency conversion beyond what is achievable with time-invariant inclusions.

**Figure 9:**
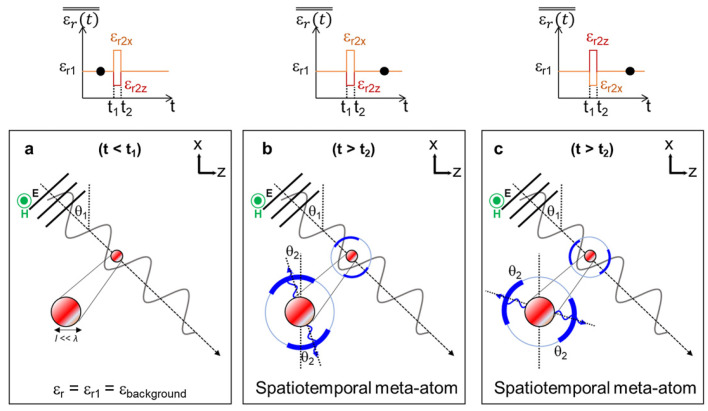
(**a**) For $t<t_{1}$, $\varepsilon_{r}=\varepsilon_{r1}$ and there is no scattering, (**b**) for $t>t_{2}$ and $\varepsilon_{1}\to \bar{\bar{\varepsilon_{r2}}}=\left[\varepsilon_{r2x},\varepsilon_{r2z}\right]\to \varepsilon_{1}$, radiated wave in the $\theta_{2}$ direction, and (**c**) for $t>t_{2}$ and $\varepsilon_{1}\to \bar{\bar{\varepsilon_{r2}}}=\left[\varepsilon_{r2z},\varepsilon_{r2x}\right]\to \varepsilon_{1}$, radiated wave in the $\theta_{2}$ direction [70].

Probing this temporal paradigm further, Pacheco-Peña and Engheta, in [70], introduced meta-atoms whose permittivity switches rapidly from an isotropic value to an anisotropic tensor, creating a temporal boundary confined to a subwavelength region. When an obliquely incident *p*-polarized wave illuminates such a particle, the sudden change in the permittivity tensor acts as a source of temporal scattering, producing a cylindrical wave emitted into the background at a controllable direction. The steering law

(31) $$\theta_{2}=\tan^{-1}\;\left(\tan\theta_{1}\;\frac{\varepsilon_{r2z}}{\varepsilon_{r2x}}\right)$$

expresses the fact that the wavenumber remains unchanged across the temporal jump while the Poynting vector rotates by an amount set by the anisotropy ratio. Figure 9 schematically depicts this local temporal switch and the resulting steered emission [70]. Time-domain simulations confirm the steering relation for different particle shapes and incidence angles, and the deep subwavelength nature of the inclusion justifies a dipolar description. Beyond steering, the authors uncover intrinsically asymmetric, nonreciprocal scattering. When the illumination is reversed so that the new incidence matches the previously emitted angle, the response does not retrace its original path, reflecting the combined action of anisotropy and temporal discontinuity. The duration of the temporal window determines whether the response is purely temporal or mixed spatiotemporal. When the switch is brief, spatial scattering is negligible, whereas longer windows allow both spatial and temporal features. The study identifies a four-dimensional design framework in which temporal anisotropy at the meta-atom level enables real-time beam steering and direction selective scattering, together with compact design rules expressed through an angle law, offering a path toward programmable, nonreciprocal metasurfaces without uniform global modulation.

These works provide a comprehensive overview of temporal electromagnetic circuits and meta-atoms. Distributed modulation in transmission lines provides broadband, low-loss, and magnet-free nonreciprocity and faithfully reproduces temporal crystal physics in practical RF hardware. Local temporal modulation at the meta-atom level reconfigures scattering, enables energy accumulation and release, and drives deterministic frequency shifts. The combination of these approaches forms a hardware toolbox for spatiotemporal metamaterials in which temporal control is applied either collectively across many unit cells or locally within a single inclusion, expanding the range of achievable functionalities while remaining compatible with integrated and reconfigurable platforms.

### 3.4. Stochastic Variations in Time-Varying ParametersDD

Temporal modulations in realistic platforms rarely remain perfectly periodic, and even small fluctuations can reshape wave transport in ways that differ fundamentally from spatial disorder. The works in this subsection establish the statistical foundations of wave propagation in media whose constitutive parameters vary randomly in time, revealing regimes of exponential energy growth, velocity slowdown, and universal scaling laws.

**Figure 10:**
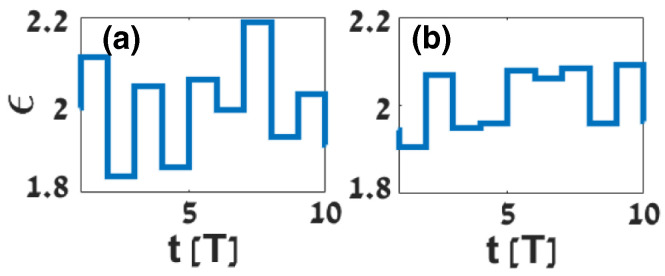
(**a**) Temporal permittivity with underlying periodicity, and (**b**) temporal permittivity without underlying periodicity [26].

Sharabi et al., in [26], examined distorted photonic time crystals (PTCs) in which a binary temporal lattice is perturbed by random fluctuations from segment to segment. The system consists of permittivity intervals of equal duration, with nominal values $\epsilon_{1,2}=2\mp 0.1$, and a uniform random perturbation Δϵ=AU[−1,1] added to each segment. A representative realization is shown in Figure 10 [26], where disorder visibly disrupts the otherwise periodic temporal sequence. Pulse propagation is computed either through an FDTD algorithm adapted to temporal interfaces or through spectral decomposition with ensemble averaging over $10^{5}$ realizations. At each temporal boundary, momentum remains conserved while the frequency adjusts to satisfy $\omega \sqrt{\epsilon \mu }=k$. Temporal reflection and refraction follow from the impedance discontinuity, governed by $Z_{i}=\sqrt{\epsilon_{i}/\mu_{i}}$, which provides an intuitive picture of the energy exchange at each stochastic step.

In essence, two central findings emerge. The introduction of temporal disorder causes the mean group velocity to decay exponentially toward zero, while the mean pulse energy grows exponentially, a sharp contrast with spatial Anderson localization, in which intensity typically decays. Increasing disorder steepens both trends, and these behaviors persist even when the underlying periodic lattice is removed. The susceptibility of individual modes depends on the Floquet band structure of the clean lattice, with states near band edges experiencing the strongest slowdown and most rapid energy growth. Statistical diagnostics reveal single-parameter scaling, reflected in the linear growth in Var[lnI] with propagation time and in the constant ratio Var[lnI]/〈lnI〉≈0.44 across disorder strengths. These results demonstrate that stochastic temporal media behave as temporal analogs of Anderson systems, yet with dynamics dominated by time-reflection physics associated with Maxwell’s second time derivative.

Moreover, Carminati et al., in [71], developed a general statistical theory for homogeneous media whose dielectric function fluctuates randomly in time, establishing universal laws that apply across wave types. By working in *k*-space, the dynamics reduce to a second-order ordinary differential equation with time-dependent frequency,

(32) $$\frac{\partial^{2}D}{\partial t^{2}}\left(k,t\right)+\Omega^{2}\left(t\right)D\left(k,t\right)=0,$$

where $\Omega^{2}\left(t\right)=c^{2}k^{2}/\epsilon \left(t\right)$. In physical terms, temporal disorder appears here as a randomly varying instantaneous oscillation frequency Ω(t), so the dynamics are dominated by phase randomization and intermittent “kicks” that redistribute energy across temporal scattering events. This explains why stochastic modulation can simultaneously slow down wave packets while driving the exponential growth of average energy, in sharp contrast to conventional spatial-disorder intuition. Each kick is represented by a 2×2 transfer matrix with unit determinant, allowing the forward and backward components of the field to be tracked and enabling a definition of the accumulated energy $U_{N}$ after *N* kicks. For long sequences with weak disorder, a random phase assumption applies and enables a Lyapunov exponent characterization of the energy growth.

Bearing in mind the above aspects, two statistical regimes arise. In the short and intermediate regime, where $1\ll N\ll N_{c}$, energy increments add incoherently and $\langle U_{N}\rangle$ grows linearly with *N*, with the full distribution following a generalized exponential form. Beyond a crossover $N_{c}∼1/\langle \beta \rangle$, the process enters a long-time regime in which $\ln U_{N}$ becomes Gaussian. Consequently, $U_{N}$ is log-normally distributed with $\langle \ln U_{N}\rangle =N\langle \beta \rangle$ and $\mathrm{Var}\left(\ln U_{N}\right)=2\langle \ln U_{N}\rangle$. This predicts the exponential growth of average energy and establishes a single parameter scaling law for the entire distribution. Extensive transfer matrix simulations of the δ-kick model confirm both regimes, supporting the universality of these results and indicating experimental relevance in systems where weak temporal disorder can be engineered.

The preceding studies constructed a coherent statistical scheme for stochastic time-varying media. Random temporal perturbations can freeze group velocities while amplifying energy, and they generate universal log-normal statistics deeply connected to Lyapunov growth. The resulting behavior departs markedly from spatial disorder models and underscores how randomness in time yields a qualitatively distinct form of wave transport with implications spanning from RF to optical platforms.

### 3.5. Temporal Interfaces

Temporal interfaces represent the most localized form of time variation, created when a medium’s constitutive parameters change abruptly at a single instant. Unlike periodic or extended temporal modulation, these boundaries convert a pre-existing field into new spectral components through continuity constraints imposed directly in time. The works summarized here establish the correct boundary conditions, extend the formulation to dispersive and moving temporal boundaries, and demonstrate ultrafast optical implementations at the single-cycle scale.

A clear formulation of electromagnetic scattering at an instantaneous change in refractive index was presented by Xiao et al. in [72], who analyzed the problem directly from Maxwell’s equations. By enforcing causality in the curl relations, the authors show that the physically meaningful conditions are the continuities of *D* and *B* at the switching instant $t_{0}$. These conditions uniquely determine the generated forward and backward waves and reveal two distinct scattering mechanisms, one linked to impedance mismatch and the other to temporal scaling of the field. Continuity of the electric and magnetic fields produces coefficients differing by a factor $\eta_{1}/\eta_{2}$ and therefore does not yield the correct physical amplitudes. Even when $\eta_{1}=\eta_{2}$ eliminates the impedance driven component, the transmitted field still undergoes amplitude rescaling and frequency conversion because time dilation changes the local optical period.

A compact expression encapsulates these effects for a nondispersive medium. If the refractive index jumps from $\eta_{1}$ to $\eta_{2}$, the transmitted field becomes a time-warped and rescaled version of the input,

(33) $$E_{t}\left(t\right)=s\;E_{\mathrm{in}}\left(s\;t-s\;T_{d}\right),\;\;s=\frac{\eta_{1}}{\eta_{2}},$$

with $T_{d}$ a constant delay. Finite difference simulations confirm the theory in both mismatched and matched cases. With mismatch, a reflected pulse appears and the transmitted amplitude agrees with the D,B based coefficients. With matching reflection vanishes but the transmitted amplitude still scales by $s=\eta_{1}/\eta_{2}$, exactly as the time transformation predicts. The study establishes a physically grounded prescription for temporal interfaces and makes clear that any abrupt temporal change in refractive index necessarily modifies frequency, amplitude, and temporal width.

Additional insight into temporal boundaries can be obtained from [73], which analyzes how dispersive waveguides respond when a pulse encounters a moving temporal interface. By transforming to the frame co-moving with the interface, the authors derive a modified dispersion relation and a pulse evolution equation containing the jump $\beta_{B}$ induced by the refractive index change. In this frame, the conserved quantity is the propagation constant $\beta_{0}$, so the pulse spectrum must relocate between the dispersion branches before and after the boundary to keep $\beta_{0}$ fixed. This conservation rule generates temporal reflection and temporal refraction, with the reflected and transmitted frequencies determined entirely by the pre- and post-boundary dispersion.

Closed-form expressions follow from solving the quadratic constraint for constant $\beta_{0}$. The reflected line occurs at

(34) $$\omega_{r}=\omega_{0}-\frac{2\;\Delta \beta_{1}}{\beta_{2}},$$

while the transmitted line is

(35) $$\omega_{t}=\omega_{0}+\frac{\Delta \beta_{1}}{\beta_{2}}\left[-1+\sqrt{1-\frac{2\;\beta_{B}\;\beta_{2}}{{\left(\Delta \beta_{1}\right)}^{2}}}\right].$$

When $\sqrt{2\beta_{B}\beta_{2}}>\Delta \beta_{1}$, no real transmitted solution exists, and the pulse undergoes temporal total internal reflection, a complete reflection in time enabled by dispersion. Simulations confirm large spectral displacements in the reflected component, bending of the transmitted pulse, and narrowing or broadening linked to the curvature of $\beta_{0}\left(\omega \right)$. The work clarifies how dispersion sets the direction and magnitude of temporal frequency shifts and delineates conditions for observing reflection, refraction, and total internal reflection (TIR) in guided wave platforms.

Single-cycle temporal boundary effects were investigated by Lustig et al. in [74], providing an experimental view of time refraction and reflection at ultrafast index transitions. Using pump-probe experiments on transparent conducting oxides near their epsilon near zero-point, Ref. [74] demonstrates time refraction and time reflection generated by femtosecond scale index switching. A pump induces rapid changes in the permittivity of an indium tin oxide (ITO) film while a probe at 1225 nm traverses it. When the index rises during the probe’s presence, the transmitted spectrum red shifts, and when the index falls, it blue shifts. Because the probe and the modulation overlap strongly in time, distinct portions of the same pulse experience opposite slopes of the index ramp, producing simultaneous red and blue components in spectrograms. Measured intensity variations in transmission and Fresnel reflection corroborate the spectral behavior, and a transfer matrix estimate reveals index changes up to ∼0.5, with ∼0.12 achieved within a single optical cycle.

A minimal dynamical model links the pump-induced change Δε(t) to the observed frequency translation. The perturbation evolves according to a driven relaxation dynamic, $d\Delta \varepsilon /dt\approx I_{\mathrm{mod}}\left(t\right)-\Delta \varepsilon /T_{\mathrm{decay}}$, and the instantaneous index is taken as $n\left(t\right)=ℜ\left\{\sqrt{\varepsilon_{\mathrm{ambient}}+\Delta \varepsilon \left(t\right)}\right\}$. Steeper and faster ramps correspond to larger spectral shifts, matching experiments. To this end, FDTD simulations further reproduce the overlap of red and blue when the probe is long, and show that blue shift disappears if the relaxation time is hundreds of femtoseconds. This allows the experiment to infer a remarkably fast relaxation of approximately 20–30 fs in ITO, much shorter than commonly assumed phonon-limited values. Companion measurements on aluminum zinc oxide (AZO) yield weaker blue shifts and longer relaxation, demonstrating material-dependent tunability. The study establishes that optical temporal interfaces with large Δn and sub-cycle switching are experimentally feasible and opens pathways to photonic time crystals and ultrafast temporal photonic devices.

Thus, a coherent foundation for temporal boundaries is provided by these papers. Correct boundary conditions based on the continuity of *D* and *B* ensure physically meaningful amplitudes and frequency shifts. Dispersive and moving interfaces introduce new conservation rules that govern temporal reflection, temporal refraction, and temporal total internal reflection. Ultrafast experiments demonstrate that few-femtosecond temporal boundaries with large index excursions are experimentally attainable, placing temporal interfaces at the core of emerging ultrafast and nonstationary photonic technologies.

### 3.6. Kramers–Kronig Generalization

A rigorous entry point into dispersion under temporal modulation is provided in [75], which formulates a linear time-variant (LTV) framework for polarization in time-varying, dispersive media and extends the classical Kramers–Kronig (KK) relations to this setting. Drawing on tools from LTV systems theory, the authors describe a dynamic material through its impulse response h(t,τ), and show that it is often more transparent to work with the input delay-spread function $c\left(t,\hat{\tau }\right)=h\left(t,t-\hat{\tau }\right)$ or the output delay-spread function $h_{c}\left(\hat{\tau },\tau \right)=h\left(\tau +\hat{\tau },\tau \right)$.

For a Lorentz medium with a time-dependent density of oscillators, the macroscopic polarization satisfies

(36) $$\ddot{P}+\gamma \dot{P}+\omega_{0}^{2}P=\epsilon_{0}\omega_{p}^{2}\left(t\right)\;E,$$

which, in the frequency domain, becomes

(37) $$P\left(\omega \right)=\epsilon_{0}\frac{\frac{1}{2\pi }A\left(\omega \right)\underset{\omega }{*}E\left(\omega \right)}{\left(\omega_{0}^{2}-\omega^{2}+i\gamma \omega \right)}$$

where $A\left(t\right)=\omega_{p}^{2}\left(t\right)$. This structure leads directly to a separable representation

(38) $$h_{c}\left(\hat{\tau },\tau \right)=A\left(\tau \right)\;\chi \left(\hat{\tau }\right),$$

with χ the standard linear time-invariant (LTI) Lorentz kernel, making explicit how temporal selectivity (through *A*) and frequency dispersion (through χ) factorize. A parallel RLC analogy further clarifies how time variations enter impedances and admittances, revealing why LTI identities do not carry directly into the LTV regime. In the Lorentz case, however, the total impedance factorizes in a way that yields closed-form responses to impulsive excitations and highlights the dual dependence on excitation time τ and observation time *t*. Reformulating the problem in mixed Fourier domains produces time-varying transfer functions that recover familiar limits, including the standard wave equation for a time-varying plasma.

The generalized KK relations arise from causality along the delay axis. Since a causal LTV medium satisfies h(t,τ)=0 for t<τ, both $c(t,\hat{\tau })$ and $h_{c}\left(\hat{\tau },\tau \right)$ vanish for $\hat{\tau }<0$. This one-sided nature guarantees analyticity in the delay frequency variable $\hat{v}$ and enables Hilbert transform relations between the real and imaginary parts of the transformed response. Additionally, Ref. [75] derives KK-type identities for the mixed-domain quantities $C_{\omega ,\hat{v}}\left(\omega ,\hat{v}\right)$ and $H_{C,\hat{v},v}\left(\hat{v},v\right)$, arguing that $h_{c}$ provides the physically clearest vehicle for such relations. Numerical examples for media with time-varying plasma frequency and time-varying resonance frequency illustrate how dispersion inherits an explicit dependence on τ. Overall, this work provides a rigorous functional methodology for polarization and dispersion in linear time-varying media and establishes generalized Kramers–Kronig relations for temporally driven systems.

### 3.7. Non-Hermitian Temporal Design

A complementary direction in temporal metamaterials is provided in [76], which introduces a non-Hermitian strategy based on enforcing spectral causality. The central idea is that if the temporal modulation of a medium’s complex susceptibility is engineered so that its real and imaginary components form an anti-Kramers–Kronig (anti-KK) pair,

(39) χre(t)=−1πP∫−∞∞χim(T)t−TdT,χim(t)=−1πP∫−∞∞χre(T)t−TdT,

then its temporal Fourier transform satisfies $\tilde{\chi }\left(\omega <0\right)=0$. In physical terms, the anti-KK constraint ties gain and loss to the programmed index trajectory so that the modulation has a one-sided temporal spectrum, enforcing a directional “frequency flow” that suppresses down-conversion. This ensures that temporal scattering never produces frequencies below the input carrier, providing a direct means to suppress down-conversion in any order of perturbation. The paper derives these anti-KK relations from first principles and clarifies the precise conditions under which they guarantee spectral causality.

To explain the mechanism, Ref. [76] begins with a dispersionless approximation, which isolates the role of temporal convolution in suppressing negative-frequency generation. They subsequently show that physical realizability requires dispersion. At every time *t*, the frequency response $\chi (t,\omega^{\prime })$ must obey the conventional KK relations in $\omega^{\prime }$, while along the time axis, the modulation envelope should satisfy the anti-KK conditions over the programmed interval. The requirements define a broad design space for complex temporal modulations in which gain and loss are not arbitrary but are tied to the desired refractive index trajectory. The formulation is cast as a temporal scattering series for the displacement field, making explicit how the convolution with $\tilde{\chi }\left(\omega \right)$ redistributes spectral components and why enforcing $\tilde{\chi }\left(\omega <0\right)=0$ forbids down-conversion at all perturbative orders.

Three illustrative applications highlight the reach of this formulation. First, by coordinating the time variation in resonance frequency and loss in a Lorentz medium so that the combined susceptibility satisfies the KK/anti-KK duality, one obtains broadband absorption without reflection, even when the static medium is strongly impedance-mismatched. Second, pairing a refractive-index change with its anti-KK gain and loss counterpart enables a form of temporal cloaking in which both forward- and backward-scattered waves at the carrier are canceled and the incident waveform is restored after the modulation window. Furthermore, the stability constraints and power requirements associated with temporally localized gain are analyzed. Third, spectrally causal modulation produces unidirectional mode coupling in a ring resonator, enabling nonreciprocal frequency translation without traveling-wave modulation or cascaded phase modulators. Because only higher frequencies are generated, the device operates as a one-way conveyor in synthetic frequency space. These results establish non-Hermitian temporal design as a flexible framework for shaping wave evolution in time, where anti-KK engineering enables perfect absorption, temporal invisibility, and one-way frequency conversion with feasible implementations in circuits and photonic platforms.

The works reviewed in this section establish the theoretical basis of temporal and spatiotemporal metamaterials. Time variation in permittivity, permeability, and conductivity reveals how frequency conversion, parametric gain, synthetic motion, and momentum gaps emerge from fundamental constraints such as wavenumber conservation and temporal boundary conditions. Stochastic modulation introduces distinct regimes of energy growth and universal scaling, while temporal interfaces show how abrupt changes reshape frequency, amplitude, and pulse structure in both nondispersive and dispersive settings. The extension of Kramers–Kronig relations to time-varying systems clarifies the role of dispersion in linear time-variant media, and non-Hermitian temporal design demonstrates that the coordinated control of index and gain and loss profiles enables spectrally causal responses and one-way frequency translation. The combined theoretical insights presented here underpin the device-level concepts and applications examined in the following sections.

## 4. Functional Applications of Time-Varying and Space–Time– Varying Metamaterials

The theoretical principles reviewed in the previous section translate into a broad and rapidly expanding set of practical functionalities enabled by temporal and spatiotemporal modulation. By deliberately engineering the exchange of energy and momentum between electromagnetic waves and time-varying media, temporal metamaterials provide routes to nonreciprocal transport, tunable absorption, broadband frequency translation, dynamic impedance matching, and parametric amplification beyond the limits imposed by linear time-invariant systems. At the system level, these effects enable new classes of devices that operate without magnetic bias, exhibit ultrafast reconfigurability, and can be programmed directly in the temporal domain. This section surveys the main application areas that have emerged to date, with an emphasis on experimentally demonstrated platforms and physically grounded design principles. The discussion is organized around nonreciprocity, absorption, frequency conversion, impedance matching, amplification, temporal cloaking, beam control, and diffusive spatiotemporal transport, highlighting both common mechanisms and performance metrics tailored to each application. It is important to note that while spacetime modulation provides a powerful and unified framework for achieving many of the functionalities discussed in this section, comparable effects can also be realized using alternative approaches, including programmable metasurfaces, nonlinear geometric-phase control, disorder-enabled designs, and passive asymmetric architectures, each subject to different physical constraints and trade-offs.

### 4.1. Nonreciprocity

**Figure 11:**
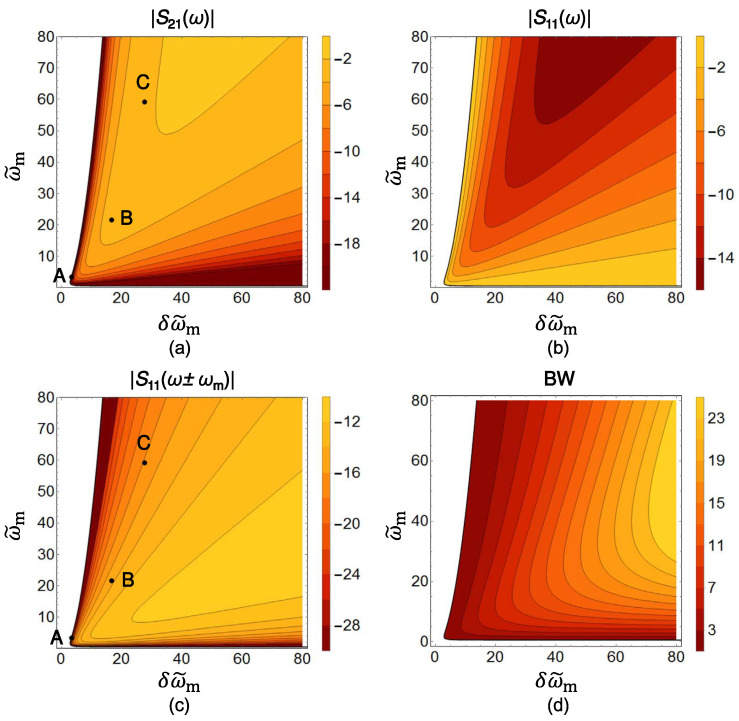
(**a**) Insertion loss, (**b**) return loss, (**c**) intermodulation product, and (**d**) bandwidth for ${\tilde{\omega }}_{m}=\omega_{m}/\gamma_{+,loss}$ and $\delta {\tilde{\omega }}_{m}=\delta \omega_{m}/\gamma_{+,loss}$ [77].

A foundational route toward magnet-free nonreciprocal devices based on spatiotemporal modulation is presented in [77], which develops a rigorous circuit-level theory and experimental validation of microwave circulators constructed from rings and wye loops of resonators whose eigenfrequencies are modulated with a traveling $120^{\circ }$ phase pattern. The spatiotemporal drive imparts synthetic angular momentum to the system, lifts the degeneracy of counter-rotating modes, and thereby breaks Lorentz reciprocity without magnetic bias. Using coupled-mode theory, the authors derive closed-form scattering relations for a three-resonator loop and identify the exact operating point that nulls one port through destructive interference between quasi-rotating eigenstates. The analysis quantifies intermodulation products and bandwidth trade-offs as functions of modulation frequency and depth, while design charts map insertion loss, return loss, intermodulation level, and bandwidth over the normalized modulation space. Figure 11 presents the prototype and corresponding *S*-parameters [77], highlighting the null response at the isolated port and the high isolation achieved at the circulation operating point, in close agreement with the theory.

Two hardware realizations follow from this model. A lumped-element wye resonator operating near 200 MHz employs varactor-based frequency modulation and filtering to confine the low-frequency drive and achieves isolation exceeding 50 dB with approximately 3–4 dB insertion loss. A distributed wye implementation near 2.2 GHz replaces inductors with transmission line sections and attains similar isolation with less than 2.5 dB simulated insertion loss. The theory accurately predicts the relative strengths of intermodulation sidebands and the bandwidth and efficiency trade-off. The analysis further clarifies the power handling and linearity limits imposed by varactor nonlinearity and outlines methods for scaling the bias to reduce third-order distortion. Collectively, these results demonstrate that synthetic angular-momentum bias via spatiotemporal modulation enables compact circulators that are compatible with CMOS technology and exhibit low noise with modulation frequencies far below the RF carrier.

**Figure 12:**
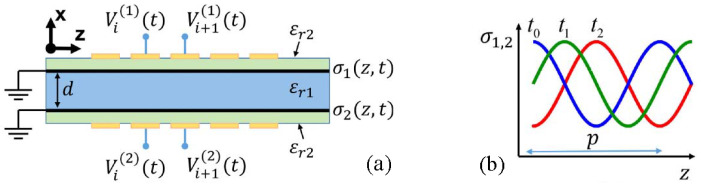
(**a**) Schematic representation of spatiotemporally modulated graphene parallel-plate waveguide, and (**b**) conductivity profile with spatial period p at $t_{0}$, $t_{1}=t_{0}+1/\left(4f_{m}\right)$, $t_{2}=t_{0}+1/\left(2f_{m}\right)$ [78].

A complementary plasmonic platform for magnet-free nonreciprocity in the terahertz regime is developed by Correas-Serrano et al. in [78], who merge space–time modulation with graphene plasmonics. The proposed structure in Figure 12 consists of a graphene parallel-plate waveguide with two independent gates, in which the chemical potential, and hence the sheet conductivity, is driven by a traveling modulation. In the Drude regime, the modulation is expressed as

(40) $$\mu \left(z,t\right)=\mu_{c0}\left[\;1+M\;\cos\;\left(\omega_{m}t-\beta_{m}z\right)\right],$$

which translates directly into a conductivity modulation with the same spatiotemporal periodicity. This drive supplies linear momentum to the guided plasmons and breaks time-reversal symmetry by selectively phase matching different waveguide modes in one direction only. Mode-coupling theory and full-wave simulations demonstrate strong directional mode conversion at a single operating frequency, establishing nonreciprocal transport on a planar platform compatible with standard silicon photonics.

Specifically, two applications are elaborated. A waveguide isolator is realized by modulating only the top graphene sheet. Backward incidence satisfies the momentum and frequency matching conditions for strong even-to-odd mode conversion, while forward incidence does not. Selecting a device length that is equal to the coherence length yields an almost complete backward conversion and strong isolation, with forward insertion loss mainly limited by the graphene relaxation time. A nonreciprocal leaky-wave antenna is further demonstrated by combining spatial conductivity corrugation with temporal modulation to asymmetrically tilt the dispersion. In transmission, guided plasmons at $\omega_{0}$ radiate predominantly at $\omega_{0}-\Omega$ in one direction, whereas in reception, an incoming plane wave is down-converted into a guided mode at a different frequency and angle. The study further quantifies the practical limits imposed by RC gating bandwidth, attainable chemical-potential swings, and graphene quality.

A related angular-momentum bias strategy implemented at the circuit level is presented by Kord et al. in [79], who developed and experimentally validated a magnet-free microwave circulator based on a loop of three resonant junctions arranged in a delta topology. Each junction consists of a parallel LC tank whose capacitance is modulated with a traveling $120^{\circ }$ phase sequence. The unmodulated network behaves as a bandstop structure, while time modulation lifts the degeneracy of the loop eigenmodes and opens a unidirectional transmission window. A small-signal linear model yields closed-form harmonic S—parameters and design charts relating insertion loss, return loss, isolation, and bandwidth to the modulation settings. Each resonator is described by

(41) $$f_{n}\;=\;\frac{1}{2\pi \;\sqrt{L\;C_{n}}},\;n=1,2,3,$$

with varactor modulation

(42) $$C_{n}\left(t\right)=C_{0}\;+\;\Delta C\;\cos\;\left(\omega_{m}t+\phi_{n}\right),\;\phi_{n}=\left(n-1\right)\cdot 120^{\circ }.$$

The $120^{\circ }$ phase progression implements an effective traveling-wave (rotating) modulation, which lifts the degeneracy of clockwise and counter-clockwise modes and thereby breaks Lorentz reciprocity without magnetic bias. In the modal basis of clockwise and counter-clockwise loop modes, the RF voltages satisfy

(43) $$V_{\pm }\left(\omega \right)=\sum_{k=-1}^{+1}H_{k}^{\pm }\left(\omega \right)\;V_{s1}\;\left(\omega +k\;\omega_{m}\right),$$

making explicit the parametric coupling among the carrier and first-order sidebands.

Hardware prototypes across both resonator loop-based implementations confirm this theory. Realizations based on lumped elements near 200 MHz achieve isolation above 50 dB with 3–4 dB insertion loss, while distributed implementations near 2.2 GHz yield comparable isolation with approximately 2.5 dB simulated insertion loss. The measured noise figure near 4.5 dB and $P_{1\mathrm{dB}}\approx 29$ dBm indicates competitive linearity for full-duplex front ends. The study further analyzes how isolation saturates with modulation depth due to residual modal coupling and discusses the suppression of intermodulation and modulation leakage.

**Figure 13:**
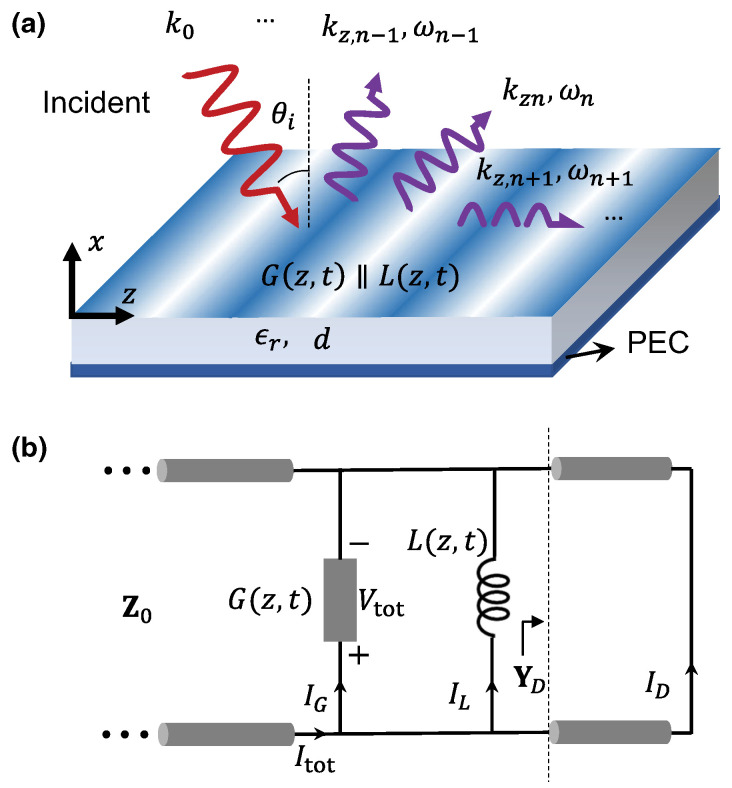
(**a**) Space–time-modulated surface and (**b**) equivalent circuit model [80].

In addition, Ref. [80] develops a unified circuit approach for engineering nonreciprocal wave transformations using a single, spatiotemporally modulated impedance sheet. As illustrated in Figure 13, the surface conductance and inverse inductance are expanded as traveling-wave Fourier series,

(44) $$\Psi \left(z,t\right)=\sum_{m}\psi_{m}e^{-jm(\beta_{M}z-\omega_{M}t)},\;\Psi \in \left\{G,B\right\}.$$

From this expansion, Toeplitz admittance matrices are assembled to capture harmonic coupling, and a compact reflection matrix is derived, linking the incident field to all scattered sidebands. The formulation reveals how modulation frequency, wavenumber, amplitude, and waveform shape the inter-harmonic pathways and produce nonreciprocal, directionally selective mode conversion without magnets.

Building on this model, several magnet-free nonreciprocal metasurfaces are synthesized through appropriate selection of the modulation and loss distribution. A lossy gradient impedance yields an isolator in which one incidence excites strongly dissipative evanescent fields while the opposite incidence propagates with low loss. A reactive profile produces a nonreciprocal phase shifter with different phase advances in the two directions. The same framework further enables quasi-isolators based on frequency conversion and retroreflection, as well as three-port metasurface circulators that route energy unidirectionally with deterministic frequency shifts. Across all examples, the synthesis procedure follows clear steps of phase matching, harmonic selection, and loss engineering, and practical implementations are demonstrated from varactor-based microwave surfaces to graphene-based terahertz sheets.

A representative photonic implementation of spatiotemporally induced isolation is provided by Yu and Fan in [81]. Their analysis establishes a linear route suitable for CMOS implementation that achieves complete optical isolation by harnessing spatiotemporal refractive-index modulation to drive indirect interband photonic transitions that shift both frequency and wave vector. In a silicon slab waveguide supporting even and odd bands, a modulation of the form Δε(x,z,t)=δ(x)cos(Ωt−qz) couples a forward mode $\left(\omega_{1},k_{1}\right)$ in band 1 to $\left(\omega_{2},k_{2}\right)$ in band 2 with $\Omega =\omega_{2}-\omega_{1}$ and phase matching $\Delta k\equiv k_{2}-k_{1}-2q\approx 0$. Under a slowly varying envelope approximation, the forward amplitudes obey a two-mode coupled-mode system,

(45) $$\frac{d}{dz}\left(\begin{array}{c} a_{1}\\ a_{2} \end{array}\right)=\left(\begin{array}{cc} 0 & i\;C\;e^{-i\Delta k\;z}\\ i\;C\;e^{+i\Delta k\;z} & 0 \end{array}\right)\left(\begin{array}{c} a_{1}\\ a_{2} \end{array}\right),$$

with the coupling coefficient of

(46) $$C\;=\;\frac{\varepsilon_{0}}{8}\int_{-\infty }^{\infty }\delta \left(x\right)\;E_{1}\left(x\right)\;E_{2}\left(x\right)\;dx,$$

which yields full conversion at the coherence length $l_{c}=\pi /\left(2|C|\right)$ when Δk=0. Importantly, the same modulation does not phase-match the time-reversed backward state $\left(\omega_{1},-k_{1}\right)$, so backward transmission remains in its original band and frequency. Combining the nonreciprocal transition with a passive filter for the converted band realizes ideal isolation within a linear time-varying but passive platform.

Full-wave FDTD simulations confirm a sinusoidal energy exchange along a long modulated section and unidirectional frequency conversion across a section of length $L=l_{c}$. Pulses incident from the left at $\omega_{1}$ are fully converted to $\omega_{2}$, whereas identical pulses incident from the right pass unperturbed. The concept is then ported to a micro-ring, where double resonance at $\omega_{1}$ and $\omega_{2}$ enables an ultra-compact isolator that preserves linearity and broadband tolerance to the input format. With realistic silicon modulation strengths and Ω in the tens of GHz, forward and backward isolation exceeding 40 dB are predicted over approximately 1.2 THz around 1.55μm, demonstrating the practicality of spatiotemporal isolation in integrated photonics. Overall, the device breaks reciprocity by co-designing the band structure and space–time modulation so that frequency conversion occurs only in one direction.

At the circuit level, [82] demonstrates a compact, magnet-free circulator that achieves giant isolation through the parametric modulation of three identical, strongly coupled resonators arranged in a loop. The core idea is to impart an effective electronic angular momentum through $120^{\circ }$ phase-shifted resonance-frequency modulations, which lift the degeneracy of the clockwise and counter-clockwise eigenstates and route power unidirectionally among the three ports. In the absence of modulation, the loop supports a common mode $|c\rangle ={\left[1,1,1\right]}^{T}$ at $\omega_{c}=\omega_{0}+2\kappa$ and two degenerate rotating modes $|\pm \rangle ={\left[1,e^{\pm i2\pi /3},e^{\pm i4\pi /3}\right]}^{T}$ at $\omega_{\pm }=\omega_{0}-\kappa$, with κ being the coupling coefficient between adjacent resonators. Turning on a traveling-phase modulation

(47) $$\begin{array}{cc} \omega_{1}\left(t\right) & =\omega_{0}+\delta \omega_{m}\;\cos\;\left(\omega_{m}t\right),\\ \omega_{2}\left(t\right) & =\omega_{0}+\delta \omega_{m}\;\cos\;\left(\omega_{m}t+\frac{2\pi }{3}\right),\\ \omega_{3}\left(t\right) & =\omega_{0}+\delta \omega_{m}\;\cos\;\left(\omega_{m}t+\frac{4\pi }{3}\right), \end{array}$$

hybridizes |+〉 and |−〉 into split states |R〉 and |L〉 with a frequency separation set by $\delta \omega_{m}$ and $\omega_{m}$, producing a synthetic spin that biases propagation without magnets. A coupled-mode formulation makes this explicit and leads to a circulation condition expressed as a phase relation between the two split states at the ports. Selecting $\delta \omega_{m}$ so that the relative phase is π/6 nulls one port and directs power to the other, thereby realizing ideal three-port circulation in the linear regime.

Moreover, Ref. [82] implements the concept at radio frequency using three LC tanks whose capacitances are tuned by varactors on microstrip, coupled into a three-port network, and demonstrate deeply subwavelength operation. Measurements report isolation above 40 dB over a tunable 150–210 MHz band and up to six orders of magnitude in forward- and backward-transmission contrast at the design point, with excellent agreement between experiment, full-wave simulation, and circuit theory. A key advantage over uniformly modulated rings is unitary modulation efficiency, whereby the applied modulation entirely contributes to splitting the rotating modes, which in turn lowers the required modulation depth to well below that of ring discretizations. The platform is linear, passive, and bias-free, and its topology and operating band are reconfigurable in real-time via the modulation and dc bias, making it a scalable solution for integrated RF isolation and full-duplex front ends.

Collectively, prior studies demonstrate that spatiotemporal modulation provides a unifying and physically transparent route to nonreciprocity and isolation across vastly different platforms, from integrated photonics to lumped and distributed RF circuits. In all cases, nonreciprocity emerges from the synthetic momentum or angular-momentum bias supplied by the modulation, rather than from magnetic materials or nonlinear thresholds. Optical implementations exploit interband transitions and frequency conversion, with distinct responses for different propagation directions, while circuit- and resonator-based realizations harness rotating-mode splitting and parametric interference to achieve deep isolation and circulation with high linearity and reconfigurability. Isolation is established as a mature and technologically viable application of temporal metamaterials, with clear pathways toward scalable, low-loss, and magnet-free nonreciprocal components.

### 4.2. Absorption Beyond LTI Bounds (Temporal Switching)

A representative theoretical foundation for exceeding classical absorption bounds through temporal control is provided in [83], which introduces a matching paradigm that bypasses the Bode–Fano limitation by dynamically switching the parameters of a transmission line while a pulse propagates through it. The central idea is to remove time invariance, which is the key assumption underlying classical matching bounds. In their analysis, a pulsed source excites a load through a line whose characteristic impedance and phase velocity switch between two prescribed states during the signal transit. At the switching instant, the continuity of charge and magnetic flux splits the incident waveform into forward and backward components, while the temporal change in velocity compresses or dilates the pulse without introducing the strong waveform distortion typical of narrowband, time-invariant matching networks.

Power delivery is formulated through a rigorous energy balance that accounts for both the energy drawn from the source and any energy exchanged with the switching mechanism. This leads to a constrained optimization over two impedance states and the velocity ratio. Three distinct operating regimes emerge from this balance. One corresponds to purely passive operation with no net energy exchange through switching. A second regime enables net absorption enhancement. A third regime admits net energy injection into the signal, which allows for efficiencies that exceed the classical time-invariant bound. In the passive regime, the optimal switching protocol significantly increases transmission efficiency for large source-to-load impedance contrasts and for dispersive loads. In the active regime, the time-switched line functions as a sampler followed by an amplifier, where the waveform is first sensed under one impedance state and energy is later injected under the second. Circuit models and time-domain simulations validate these predictions and indicate practical implementations using switched inductors, varactors, and reconfigurable metamaterial transmission lines.

Beyond fundamental limitations, this approach enables the efficient, low-distortion transmission of short pulses in scenarios where conventional matching fails, including electrically small antennas, ultra-wideband communications, dispersive acoustic interfaces, and broadband cloaking structures. The inherent ability to control propagation velocity also enables a tunable physical time delay as an additional degree of freedom.

**Figure 14:**
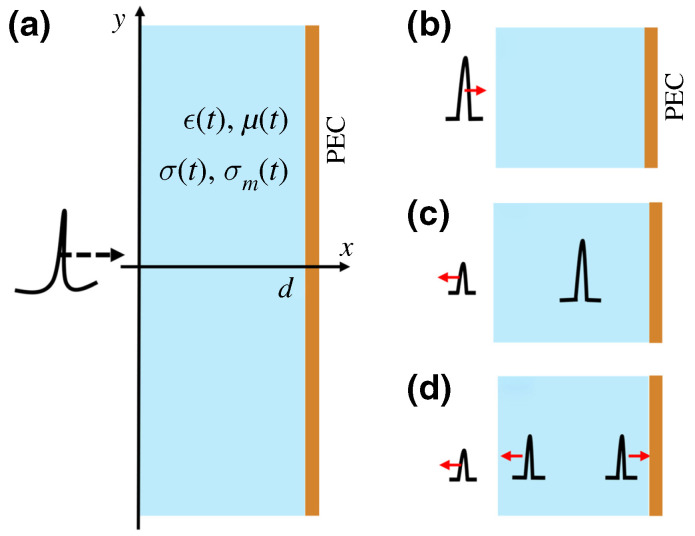
(**a**) Problem illustration, (**b**) the incident short pulse impinges the boundary ($t=t_{0}$), (**c**) the wave is within the layer ($t=t_{s}$), and (**d**) The electric/magnetic properties can lead to dispersive waves ($t>t_{s}$) [84].

A complementary and experimentally oriented investigation of temporal switching for enhanced absorption is introduced in [84], which analyzes short-pulse interaction with a lossy slab backed by a perfect conductor while the slab permittivity and conductivity are switched in time. The system is reformulated as an equivalent transmission line governed by time-domain telegrapher equations,

(48) $$\begin{array}{cc} \frac{\partial V(x,t)}{\partial x}+L\frac{\partial I(x,t)}{\partial t}+RI\left(x,t\right) & =0,\\ \frac{\partial I(x,t)}{\partial x}+C\frac{\partial V(x,t)}{\partial t}+GV\left(x,t\right) & =0, \end{array}$$

from which a time-domain Green function is derived in the Laplace domain and used to reconstruct voltages and currents after the switching event. Figure 14 illustrates the configuration and marks the instant at which the material parameters switch while the pulse resides inside the layer, which is the key mechanism enabling enhanced absorption.

Three operating scenarios are compared, namely a time-invariant slab constrained by the classical Rozanov thickness and bandwidth bound, an abruptly switched slab, and a gradually switched slab in which the parameters evolve through a staircase sequence of small temporal steps. The analysis shows that properly timed switching enables total absorbed energy that exceeds the time-invariant bound when performance is evaluated over the full signal spectrum. This enhancement persists for ultra-wideband pulses, quasi-monochromatic waveforms, and even narrowband signals. Gradual switching retains most of the performance benefit while easing realizability and reducing temporal boundary reflections.

Furthermore, Ref. [84] launches a practical realization based on a three-dimensional array of loaded resistive dipoles whose effective permittivity and conductivity are reconfigured by tuning only the terminal resistance. This architecture enables a transition from a nearly lossless dielectric state to a strongly absorbing state with reduced effective permittivity and finite conductivity. Full-wave parameter extraction confirms the targeted material properties before and after switching. Additional analysis shows that performance gains persist even when the switching time exceeds the pulse width by several factors, owing to the improved spectral shaping of the reflected field. Contour maps of absorbed energy relative to the Rozanov bound identify the conductivity and bandwidth regions in which the time-varying absorber outperforms any time-invariant counterpart, thereby providing concrete design guidelines. Overall, the study establishes a rigorous time-domain technique, demonstrates that temporal switching can exceed classical absorption limits for finite-duration signals, and delivers a viable metamaterial implementation that connects circuit-level control to macroscopic absorber performance.

A collective assessment of these works reveals that temporal switching significantly extends the attainable space of absorption and impedance matching beyond the constraints imposed by linear time-variant systems. By appropriately timing changes in material or circuit parameters while a signal resides inside a structure, it becomes possible to redistribute energy between the wave and the medium in a controlled manner, enabling both enhanced passive absorption and active energy injection. These results establish temporal switching as a powerful and general strategy for broadband pulse matching and ultrawideband absorption in realistic electromagnetic platforms.

### 4.3. Frequency Translation

Time variation provides a fundamentally linear route to frequency translation that bypasses phase matching, nonlinear susceptibilities, and long interaction lengths, enabling compact and broadband wavelength control across photonic and electromagnetic platforms. Notomi and Mitsugi [85] showed that a single high-*Q* optical cavity with a time-varying refractive index can convert the wavelength of trapped light adiabatically, without invoking nonlinear mixing. Using two- and three-dimensional FDTD simulations of photonic-crystal microcavities, the authors excited a resonant mode and then smoothly tuned the refractive index within the cavity region. The intracavity field followed the shifting eigenfrequency and, after the tuning event, the spectrum collapsed entirely to the new cavity resonance, with essentially complete conversion efficiency and no residual spectral component at the original wavelength. The magnitude of the wavelength shift scaled linearly with the index change and remained independent of the tuning speed over femtosecond and picosecond ramps as long as adiabatic continuity was preserved.

The underlying physical principle is the conservation of action rather than energy. As the resonance red shifts under an index increase, the stored energy, *U*, decreases so that the adiabatic invariant, *J*, remains constant,

(49) $$\frac{\Delta \lambda }{\lambda }=-\;\frac{\Delta U}{U},\;\;J=\frac{U}{\omega }\;=\;\mathrm{constant}.$$

The authors established quantitative design criteria for adiabatic operation, including sufficient mode spacing, spatially uniform tuning, and a cavity photon lifetime exceeding the tuning duration. Practical tuning mechanisms were surveyed, including carrier plasma, Kerr, thermo-optic, and electro-optic effects, with index swings and speeds compatible with state-of-the-art photonic-crystal cavities. The study identified adiabatic temporal tuning as a robust linear method for translating photon frequency in long-lifetime cavities, with clear and experimentally realistic design rules.

**Figure 15:**
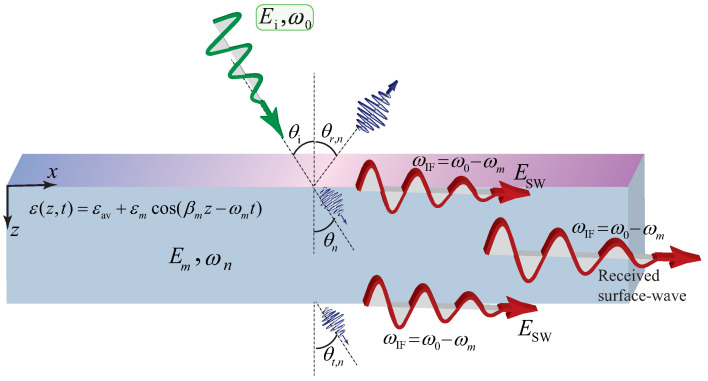
Antenna-mixer amplifier (spatiotemporal medium): transition of the incident wave to spacetime surface wave [86].

A complementary spacetime approach to frequency translation is developed in [86], where a traveling-wave modulation of permittivity enables simultaneous reception, transmission, and frequency up- and down-conversion within a single ultrathin slab. The slab permittivity was driven as a traveling wave,

(50) $$\varepsilon \left(z,t\right)=\varepsilon_{\mathrm{av}}+\varepsilon_{m}\;\cos\;\left(\beta_{m}\;z-\omega_{m}\;t\right),$$

which enforces controlled conversion between Floquet harmonics $\omega_{n}=\omega_{0}+n\omega_{m}$ while suppressing spurious sidebands in the subluminal modulation regime. This traveling-wave modulation makes the frequency conversion process directional because it simultaneously supplies an energy quantum ($\omega_{m}$) and a momentum bias ($\beta_{m}$). As a result, harmonic generation becomes a phase-selective mode-coupling problem, which is why space–time modulation can realize clean translation with controllable suppression of unwanted channels. By selecting the incidence angle such that the n=−1 harmonic became a spacetime surface wave at the slab boundary, energy was transferred from a free-space plane wave at $\omega_{0}$ to a guided surface mode at $\omega_{IF}=\omega_{0}-\omega_{m}$ in reception and reversed in transmission. The incidence rule,

(51) $$\theta_{i}=\sin^{-1}\;\left(1-\frac{\omega_{m}}{\omega_{0}}\right),$$

launches the desired harmonic tangentially along the interface. Figure 15 illustrates this surface-wave excitation condition and its geometric origin [86].

**Figure 16:**
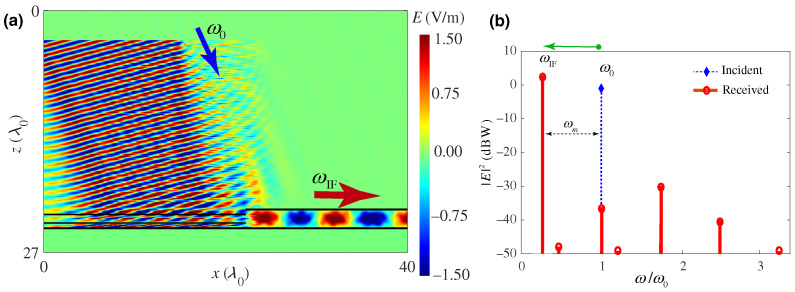
(**a**) FDTD illustration of the medium, and (**b**) frequency domain response [86].

At the operating point, the n=0 harmonic remained propagating while the n=−1 harmonic became evanescent along the normal direction, guaranteeing clean conversion to a mode bound to the surface. Full-wave simulations confirmed strong suppression of undesired harmonics and large frequency shifts. In the downlink configuration, the slab thickness

(52) $$d=\frac{2\pi }{\left|\varepsilon_{m}\;k_{0}\;k_{-1}\right|}\left|\sqrt{\gamma_{z,-1}\;\gamma_{z,0}}\right|$$

maximized the surface-wave transfer, and the corresponding power gain

(53) $$G_{d}=\frac{{\left|E_{-1}\left(z=d\right)\right|}^{2}}{{\left|E_{i}\right|}^{2}}=\frac{\omega_{-1}^{2}\;\beta_{m}}{\omega_{0}^{2}\;\alpha_{z,-1}}$$

quantified how modulation parameters controlled amplification. Figure 16 reports the validation at the system level [86], including time-domain evolution and spectral purity, in close agreement with the space–time dispersion analysis. The work framed space–time metasurfaces as compact antenna-mixer amplifier front ends capable of directional radiation, pure frequency translation, and one-way gain within a single ultrathin platform.

An ultrafast experimental realization of time refraction near an epsilon near-zero (ENZ) point is reported in [87], where sub-picosecond index modulation produces large, broadband frequency shifts in a homogeneous thin film. When the refractive index inside a medium changes while a wave is propagating within it, the frequency shifts while the wave vector remains conserved. The conservation law at the temporal boundary takes the form $n_{1}f_{1}=n_{2}f_{2}$, so that

(54) $$\Delta f=f_{2}-f_{1}=-\frac{\Delta n}{n_{1}+\Delta n}\;f_{1}.$$

The authors exploited the large index sensitivity near the ENZ point of a 620 nm indium tin oxide film and used an ultrafast pump to induce unity-order index excursions on a sub-picosecond time scale. A probe traversing the film experienced either a redshift or blueshift depending on whether it encountered aon rising or falling index during propagation. Spectrograms, which are dependent with the delay, revealed controlled bidirectional translation with shifts up to 14.9 THz near the ENZ wavelength.

A temperature model of carrier dynamics captured the rapid rise and slower decay of the index change driven by the pump, explaining the experimentally observed bias toward stronger redshifts. Because the wave vector is conserved, the process required no phase matching and fundamentally different from four-wave mixing. The achieved bandwidth was set by the time scale and magnitude of the index change rather than by the interaction length, which was only a subwavelength film. The study established ENZ platforms as powerful media for broadband, ultrafast frequency translation through purely temporal mechanisms.

Overall, the aforementioned studies yielded three complementary linear frequency-translation mechanisms that are enabled by temporal modulation. Adiabatic cavity tuning enables distortion-free wavelength conversion through eigenfrequency tracking. Space–time metasurfaces enable directional up-conversion and down-conversion with simultaneous radiation and gain through Floquet surface-wave engineering. Epsilon near-zero temporal boundaries enable ultrafast, broadband time refraction with subwavelength interaction lengths. Collectively, they establish time-variant media as a versatile and scalable foundation for compact, broadband, phase-matching, and frequency translation across the electromagnetic spectrum.

### 4.4. Impedance Matching

**Figure 17:**
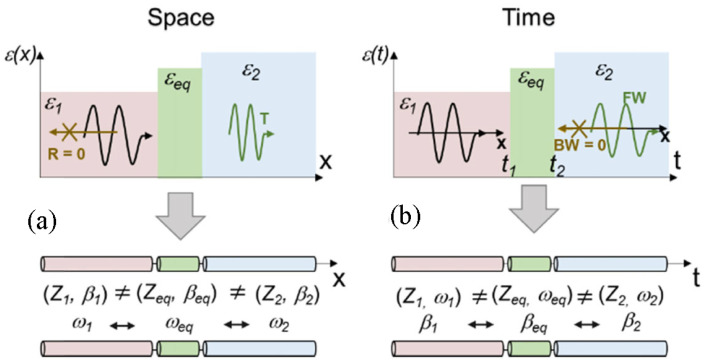
(**a**) Spatial quarter-wave impedance transformer, and (**b**) temporal quarter-wave impedance transformer [88].

Temporal impedance matching extends the concept of reflection cancellation from static media to dynamically reconfigured materials, where impedance transformations are achieved through controlled time variation rather than through inserted spatial layers. A representative temporal analog of classical impedance transformers is described in [88], which introduces a time-domain counterpart of the quarter-wave impedance transformer. Instead of inserting a spatial matching layer between two media, the authors program the permittivity in time so that a narrowband modulated pulse experiences two time steps, from $\varepsilon_{1}$ to an intermediate $\varepsilon_{eq}$ for a designed duration, then to $\varepsilon_{2}$. Using space–time duality, they derive the temporal quarter-wave matching conditions and show that a properly chosen intermediate state cancels the backward wave generated at a time discontinuity while simultaneously producing the expected frequency conversion by time refraction. The key relations explicitly appear as

(55) $$\varepsilon_{eq}=\sqrt{\varepsilon_{1}\varepsilon_{2}},\;\;\Delta t=nT_{eq}/4,$$

with *n* being odd, and where $T_{eq}$ is the optical period in the intermediate state. These relations show that a suitable intermediate step suppresses the backward wave generated at a temporal discontinuity while still enforcing frequency conversion through time refraction. Figure 17 sets the concept side by side with its spatial counterpart and includes the equivalent transmission line sketches, making the mechanism clear at a glance [88].

**Figure 18:**
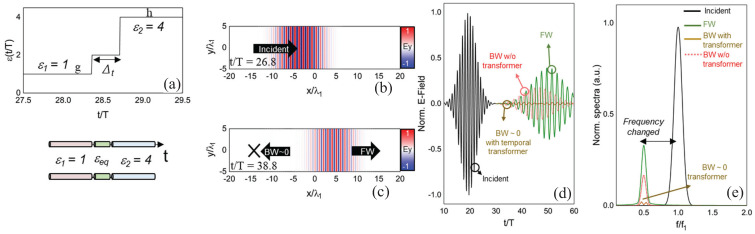
(**a**) Temporal quarter-wave impedance transformer, (**b**) numerical results of electric field distribution before the temporal boundary, (**c**) numerical results of electric field distribution after the temporal boundary, (**d**) evaluation of electric field in time, and (**e**) spectral content of electric field [88].

The study then extends the concept to unbounded media. It demonstrates spatiotemporal matching near a fixed spatial interface by applying the temporal coating only in a finite region upstream of the junction. With the timing set by the relations above, the reflected field within the signal band is strongly suppressed. Thereafter, it adapts the method to waveguide matching between different cross-sections by programming the time profile of the filling dielectric so that the effective permittivities of the guided TE_10_ modes coincide after the change, which yields high transmission with minimal reflection. Figure 18 documents these outcomes with field snapshots and spectra [88], highlighting near-zero backward components in the temporal coating case and the associated frequency conversion. Overall, these results cast impedance matching for pulses as a time-programmed operation in which changing a single material parameter twice in time achieves reflection cancellation and frequency conversion without any physical matching layer.

An alternative synthesis scheme for temporal impedance transformers was later proposed in [89] by extending the classical multilayer network theory into the time domain. Starting from the transfer-matrix description of a spatial multilayer at normal incidence, the authors construct its exact temporal counterpart as a multistep refractive-index profile in time that acts on a pulse in an otherwise unbounded medium. A closed mapping links spatial insertion loss to the backward wave generated by a temporal multistep, which forms the foundation for porting standard multilayer design rules into time. The central space–time duality identity is

(56) $$PL-1=v_{e}^{3}{\left|R\right|}^{2},$$

which ties the multilayer insertion loss PL to the temporal transformer backward-wave magnitude |R|, with $v_{e}=n_{e}/n_{i}$ as the ratio of exit and incident indices. The paper develops a constructive synthesis for two canonical families and validates them with full-wave simulations in unbounded media and also near fixed spatial interfaces, where the temporal steps are applied in a finite upstream region yet still yield nearly reflectionless frequency conversion.

**Figure 19:**
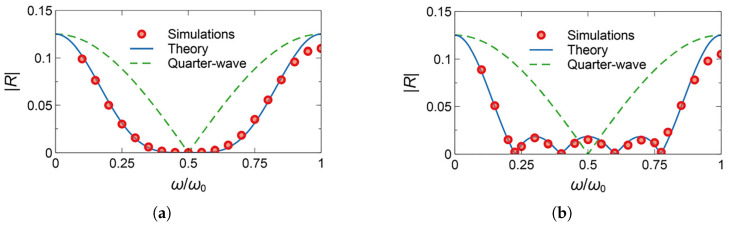
(**a**) Binomial design: simulated and theoretical results compared with the quarter-wave transformer; (**b**) Chebyshev design: simulated and theoretical results compared with the quarter-wave transformer [89].

The backward-wave target is then specified in closed form for two standard design goals. The maximally flat, or binomial, transformer uses the target law

(57) $$\left|R\left(\phi \right)\right|=\frac{|1-v_{e}|}{2v_{e}^{2}}{\left|\cos\phi \right|}^{M},$$

which suppresses the first M+1 even derivatives of |R(ϕ)| at the design point to produce a smooth passband. The equi-ripple, or Chebyshev, transformer uses the target laws

(58) $$\begin{array}{c} \left|R\left(\phi \right)\right|=\frac{|1-v_{e}|}{2v_{e}^{2}}|\frac{T_{M}\left(\sec\phi_{max}\cos\phi \right)}{T_{M}\left(\sec\phi_{max}\right)}|,\\ T_{M}\left(\sec\phi_{max}\right)=\frac{1}{R_{max}}\left(\frac{|1-v_{e}|}{2v_{e}^{2}}\right), \end{array}$$

which distribute a prescribed ripple uniformly across the band to minimize the worst-case reflection. These analytic targets determine the multistep indices and durations through the temporal transfer matrices and yield profiles that achieve deep backward-wave suppression while performing the intended frequency conversion. Specifically, Figure 19a shows the binomial transformer reaching a smooth and strongly suppressed |R| across the designed band, with close agreement between theory and simulation, while Figure 19b presents the Chebyshev transformer, which meets the specified ripple level with the expected bandwidth ripple trade-off [89].

Altogether, these works establish temporal impedance matching as a direct time-domain analog of classical spatial transformers. By exploiting space–time duality, reflection cancellation and frequency conversion are achieved through programmed material steps rather than physical layers. The transition from step quarter-wave designs to multistep binomial and Chebyshev synthesis elevates temporal matching from a conceptual construct to a systematic design methodology, enabling broadband, reflectionless pulse delivery in dynamically reconfigurable platforms.

### 4.5. Parametric Amplification

Parametric amplification in time-varying media arises from the deliberate engineering of temporal or spatiotemporal band gaps in which electromagnetic eigenfrequencies acquire an imaginary part, enabling exponential growth fueled directly by the modulation. Lee et al. in [90] introduced spatiotemporal crystals, photonic crystals with discrete spatial periodicity whose permittivity is also modulated in time. The core idea is that temporal modulation mixes positive and negative frequency branches of the unmodulated dispersion, opening momentum band gaps (MBGs), where solutions acquire complex eigenfrequencies. The imaginary part sets the parametric gain or loss, so waves launched near those MBGs can grow exponentially. The authors build this methodology in two steps. First, they compute band structures using a spacetime plane-wave expansion and verify them with full-wave simulations, showing where MBGs open and how their position and width depend on the modulation frequency and amplitude. For the lowest photonic band, MBGs typically appear at odd multiples of one half of the modulation frequency. With higher bands and realistic dispersion, additional MBGs emerge when the coupled modes have opposite frequency signs and different mode indices. Second, they truncate the crystal and show that finite structures behave as parametric oscillators when the modulation depth exceeds a transition threshold that balances gain and radiation loss. Two key features organize the dynamics above the threshold. One is the tipping time at which parametric oscillation overtakes ordinary frequency mixing. The other is a growth rate that is insensitive to input detuning and increases with modulation depth. Both quantities are mapped against the number of unit cells, revealing that longer crystals reduce the threshold and strengthen growth.

A compact modulation model captures both symmetric and directional behaviors. When all unit cells are modulated in phase, the medium exhibits symmetric MBGs and standing-wave patterns at their edges. When a spatial phase is added, the modulation becomes a traveling wave and MBGs become direction-dependent, leading to asymmetric radiation of the parametrically generated tones. The authors write the permittivity of the *n*-th slab as

(59) $$\varepsilon_{n}\left(t\right)=\varepsilon_{c}+\Delta \epsilon \cos\left(nK\Lambda -2\pi f_{m}t\right),$$

so the parameter *K* controls the spatial phase advance per cell and hence the directionality. Physically, the spatial phase term nKΛ controls whether the modulation is standing-wave or traveling-wave across the lattice. In-phase modulation produces symmetric parametric band gaps, whereas a phase-advanced drive biases the coupling and makes the gain, as well as the radiation of generated tones, direction-dependent. In the time domain, the transmitted field above threshold contains exponentially growing components at MBG frequencies in the form

(60) $$E_{\mathrm{osc}}\left(t\right)=\sum_{q}a_{q}\;\exp\;\left(2\pi g_{q}t\right)\;\exp\;\left[-\;j\;2\pi \left(\frac{2q-1}{2}\right)f_{m}t\right]+c.c.,$$

which makes explicit both the growth rate $g_{q}$ and the discrete set of oscillation frequencies tied to the MBGs. The results reveal how spatiotemporal band engineering allows one to place, widen, and bias MBGs, and how finite crystals use that programmed gain landscape to transition from frequency mixing to self-sustained parametric oscillation with tunable directionality.

A complementary quantum and classical perspective on this phenomenon is discussed in [91], by developing a clear scheme of how photonic time crystals, dielectrics whose permittivity varies periodically in time, reshape spontaneous and driven emissions. Periodic time modulation mixes positive and negative frequency branches and opens momentum band gaps in which the Floquet quasi-frequency acquires an imaginary part. Radiation coupled to these gap modes grows exponentially, drawing energy from the modulation rather than from the source itself. For a point dipole placed inside a time crystal, the emissions initially spread over all *k*, satisfying the unmodulated dispersion, and then rapidly migrate toward the gap and narrow in line width, ultimately concentrating near the midgap. This behavior motivates a time-crystal laser concept in which a finite structure channels the exponentially growing gap modes into coherent output without population inversion in the conventional sense. The study also contrasts this physics with spatial photonic crystals, where band gaps suppress emissions because the density of states vanishes. In time crystals, causality replaces energy conservation and enables amplification.

Methodologically, the analysis begins with Maxwell’s equations for a homogeneous, time-periodic medium written in *k*-space as

(61) $$\left[\frac{\partial }{\partial t}\left(\varepsilon \left(t\right)\frac{\partial }{\partial t}\right)+c^{2}k^{2}\right]H_{k}=0,$$

and proceeds to a Floquet expansion that yields two eigenmodes for each *k* and a Green-function response to an embedded current. The Green function shows that a brief excitation excites the growing solution inside the momentum gap, which explains why even a weak source ultimately produces exponential emissions powered by the modulation. Then, Ref. [91] quantizes the field with a time-dependent Hamiltonian, H, and adds a two-level emitter. Within the bands, one obtains a spontaneous emission rate

(62) $$\gamma \;=\;\frac{V}{\hbar^{2}\pi }\;\sum_{m}{\left|V_{fi}^{m}\right|}^{2}\;k_{m}^{2}\;{\left(\frac{\partial \omega_{f}}{\partial k}\right)}^{-1}{|}_{k=k_{m}}\;,$$

where $V_{fi}^{m}$ is the coupling between Floquet states separated by the *m*-th harmonic and $k_{m}$ satisfies $\omega_{f}\left(k_{m}\right)=\omega_{0}+m\Omega$. This shows that γ vanishes at the band edge as the density of states collapses. The classical and quantum treatments jointly explain two robust features above the threshold, namely a tipping time after which parametric growth overtakes ordinary frequency mixing and a growth rate that is largely insensitive to input detuning while increasing with modulation depth. The work concludes with practical cavity configurations that transform these amplifying gap modes into stable, monochromatic emissions.

Parametric amplification in time and spatiotemporal crystals emerges as a direct consequence of momentum band-gap engineering in nonstationary media. By shaping the temporal modulation profile and crystal length, one can deterministically control the onset threshold, growth rate, and radiation direction of the amplified modes. From a band theory perspective, this method unifies time-crystal oscillators and modulators and identifies spatiotemporally driven structures as a new class of gain media powered entirely by temporal modulation rather than population inversion.

### 4.6. Temporal Cloaking

**Figure 20:**
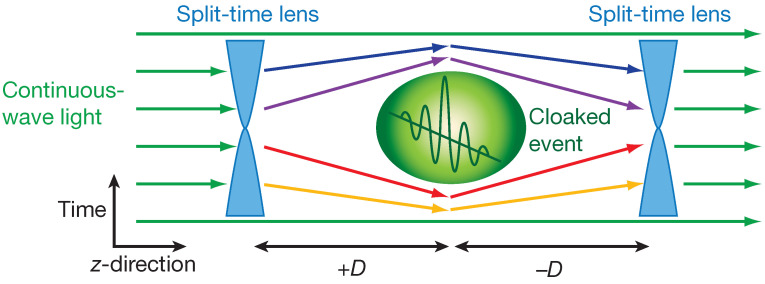
Schematic illustration of the temporal cloak using a pair of split-time lenses [95].

Temporal cloaking exploits time-variant dispersion and refractive-index modulation to create controlled intervals during which events are concealed from observation, extending the concept of invisibility from space into the time domain [92,93,94]. A first experimental realization of temporal cloaking is reported in [95], where finite-duration events are hidden inside a dynamically opened and closed temporal gap generated using time lenses and chromatic dispersion. Leveraging the space–time duality between diffraction and dispersive broadening, a split-time lens imposes opposite quadratic phase profiles on the leading and trailing portions of a continuous probe wave. When the probe then propagates through a segment with normal group–velocity dispersion, the front portion accelerates while the rear portion decelerates, thereby opening a clear temporal gap. A second dispersive segment of opposite sign, followed by a second split-time lens, recombines the waveform to its original form, restoring the probe as though no interruption had occurred. Indicatively, Figure 20 illustrates the sequence of time-lens modulation, dispersive separation, temporal gap formation, and final recombination [95]. The implementation is carried out entirely in fibers, using four-wave mixing to generate the time lenses, dispersion-compensating fibers to open the temporal gap, and standard single-mode fibers to close it, while wavelength-resolved diagnostics track the cloaking process in real-time.

Cloaking performance is verified by hiding a short, periodically occurring nonlinear interaction and showing that the event is clearly detectable when the cloak is off but is suppressed below the noise floor when the cloak is active. Increasing the pump power that drives the time lenses widens the temporal gap and deepens suppression until excess noise ultimately limits performance. Moreover, Ref. [95] identifies practical constraints, including the role of third-order dispersion in limiting achievable gap width and the onset of stimulated Brillouin scattering at high pump powers. Despite these limits, the experiment demonstrates that dispersion engineering combined with time lenses can robustly conceal finite-duration events in flight and then perfectly restore the probe, establishing a benchmark for temporal invisibility based exclusively on linear wave dynamics.

A complementary and fundamentally different cloaking paradigm is introduced in [96], which addresses a core limitation of passive cloaks, namely their inability to conceal moving objects due to Doppler frequency shifts. The proposed Doppler cloak employs a spatiotemporally modulated metamaterial coating that actively shifts the frequency of the scattered field so that the net Doppler shift that Is observed externally vanishes. The cloak permittivity is driven as a traveling wave,

(63) $$\varepsilon_{r}\left(z,t\right)=\varepsilon_{r0}+\delta \varepsilon \cos\left(\omega_{m}t+k_{m}z\right),$$

that enables nonreciprocal frequency conversion with phase matching conditions $\omega_{1}-\omega_{0}=\pm \omega_{m}$ and $k_{1}-k_{0}=\mp k_{m}$. Coupled-mode analysis predicts complete conversion over the coherence length $L_{c}=\pi /\left(2k_{C}\right)$, with $k_{C}=\left(\kappa k_{0}\right)/\left(2\sqrt{2}\right)$ and $\kappa =\delta \varepsilon /\varepsilon_{r0}$. Selecting the cloak thickness $d=L_{c}$ yields full transformation in the phase-matched direction. Because the modulation carries momentum bias, a wave retracing its path after reflection is not reconverted back to the original frequency, preserving the engineered frequency shift. One of the two converted tones performs Doppler cancellation, while the other can be suppressed through absorption or dispersion engineering.

**Figure 21:**
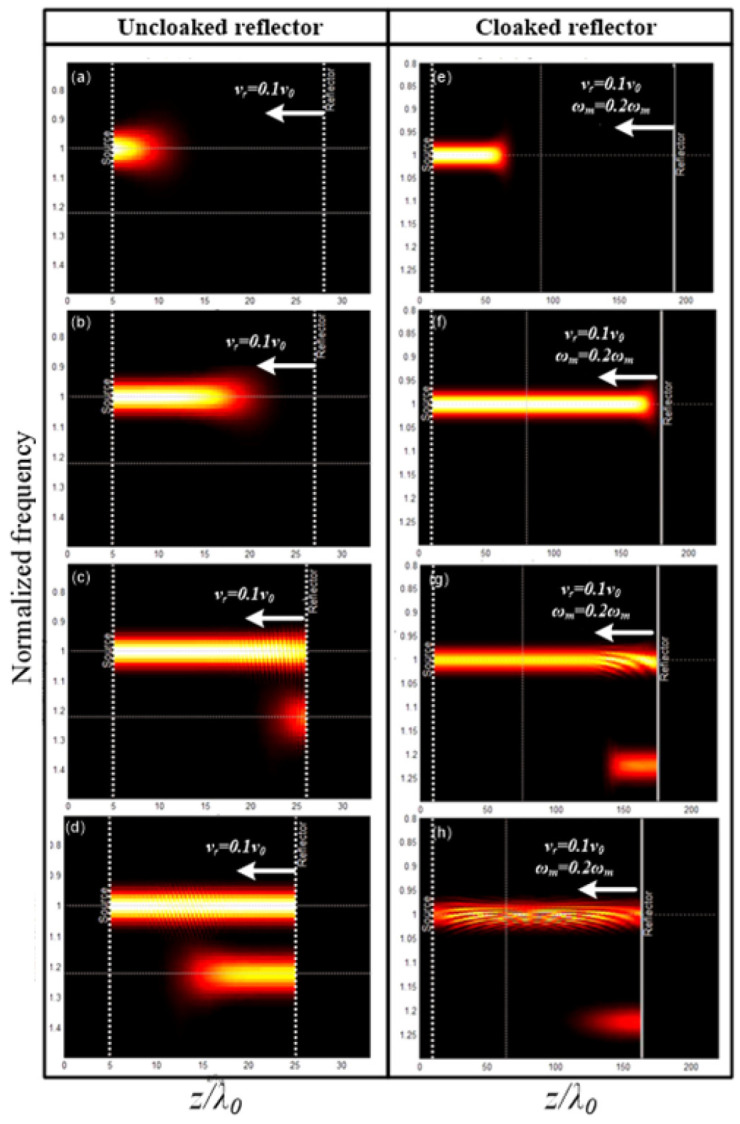
(**a**–**d**) FDTD simulations for a moving reflector without Doppler cloak and (**e**–**h**) FDTD simulations for a moving reflector with Doppler cloak [96].

To cancel the motion in the observer’s frame, the modulation frequency is chosen to offset the relativistic Doppler shift of the moving target, leading to the design rule

(64) $$\omega_{m}=2\beta \gamma \omega_{0},$$

where β=v/c and $\gamma =1/\sqrt{1-\beta^{2}}$. Under this condition, the frequency of the reflected field collapses back to $\omega_{0}$, and the composite mirror cloak system appears stationary to an external observer. Full-wave FDTD simulations verify the effect, as shown in Figure 21 [96], where a mirror moving at β=0.1 reflects at $1.22\;\omega_{0}$ without the cloak but returns exactly to $\omega_{0}$ when the cloak is active.

These studies reveal that temporal cloaking can be achieved through fundamentally different mechanisms, namely time gaps created by tailored dispersion and active spatiotemporal frequency management. While time-lens cloaks conceal events by redistributing the temporal flow of energy without altering the frequency content, Doppler cloaks actively counteract spectral shifts through nonreciprocal frequency translation. These complementary strategies establish temporal cloaking as a broader class of wave-control phenomena enabled by dynamic media, with implications ranging from optical communications to radar and the sensing of moving targets.

### 4.7. Beam Control (Steering, Combining, and Routing)

Time modulation provides a powerful means to control not only the frequency and amplitude of electromagnetic waves, but also their spatial flow of energy. By exploiting temporal boundaries and spatiotemporal modulation, it becomes possible to steer, route, and even combine beams in ways that are fundamentally inaccessible to linear time-invariant systems. A representative example is presented in [31], which introduces the concept of temporal aiming, a beam-steering mechanism achieved through an abrupt temporal transition from an isotropic to an anisotropic background medium. The central physical principle is that a temporal boundary preserves the wave vector k while rotating the Poynting vector S, thereby decoupling the direction of phase propagation from the direction of energy flow. For a *p*-polarized plane wave incident at angle $\theta_{1}$, an isotropic-to-anisotropic jump to $\varepsilon_{r}=\mathrm{diag}\left\{\varepsilon_{2x},\varepsilon_{2z}\right\}$ generates forward and backward temporal waves whose amplitudes are determined by the temporal boundary conditions, whereas the energy direction, $\theta_{s}$, obeys the simple steering law

(65) $$\theta_{s}\;=\;\arctan\;\left[\tan\left(\theta_{1}\right)\left(\frac{\varepsilon_{2z}}{\varepsilon_{2x}}\right)\right].$$

**Figure 22:**
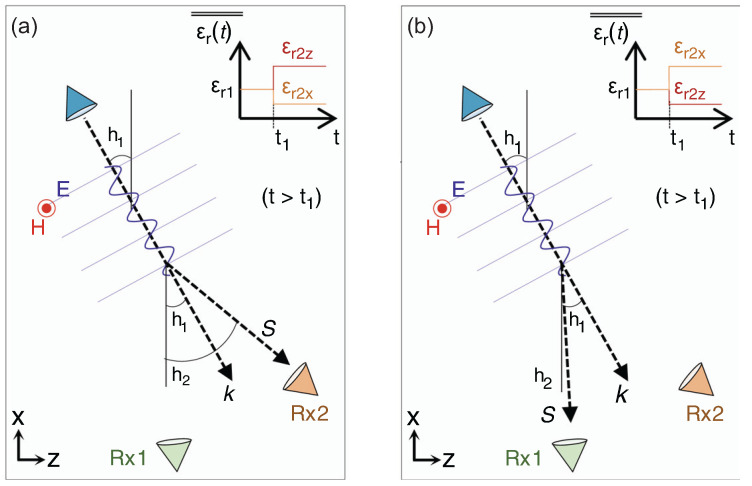
(**a**) Temporal aiming with the angle of S reaching Rx2, and (**b**) temporal aiming with the angle of S reaching Rx1 [31].

Thus, by selecting the anisotropy ratio $\varepsilon_{2z}/\varepsilon_{2x}$, energy can be deterministically redirected to any desired angle without moving the source, scanning an aperture, or imposing spatial phase gradients. As illustrated in Figure 22 [31], a sudden temporal transition from isotropic to anisotropic response preserves the wave vector while rotating the energy flow to a designed direction, enabling real-time temporal beam aiming and multireceiver routing.

Closed-form expressions for the temporal transmission and reflection coefficients are derived and validated for both plane waves and Gaussian beams. Beyond single-beam steering, the study demonstrates the routing of narrowband wave packets to multiple receivers by programming time windows during which the medium is anisotropic and then restoring the isotropic state after alignment with the desired direction. Time-domain simulations, conducted in [31], confirm that k remains invariant while S rotates according to the analytic law, with controllable forward and backward outputs. The methodology naturally generalizes from plane waves to wide and tightly focused Gaussian beams through decomposition in the angular spectrum. Conceptually, temporal aiming operates purely in the time domain and therefore complements conventional spatial beam steering, suggesting applications in real-time beam redirection, packet switching, and on-chip signal routing.

**Figure 23:**
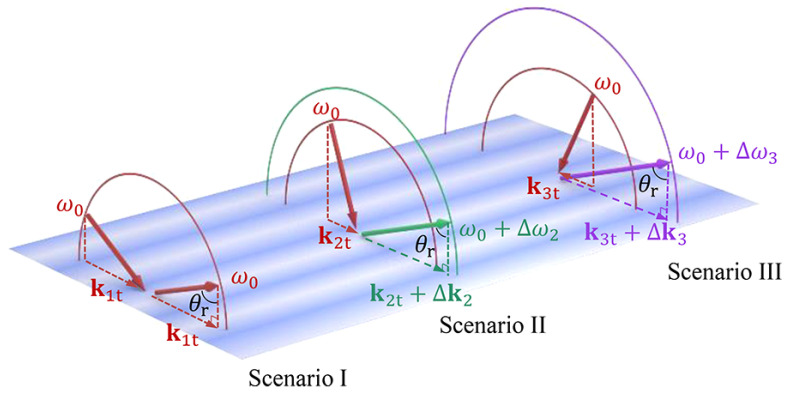
The metasurface reflects the incident waves coming from three different directions into the same direction ($\theta_{r}$) [97].

A complementary paradigm based on spatiotemporal metasurfaces enables not only routing but also a power combination that is agnostic to the input phase. This fundamental limitation of passive linear time-invariant systems is overcome in [97], where it is shown that complete phase-insensitive combining can be achieved through joint space–time modulation. The specific approach employs a reflective metasurface whose spatial modulation equalizes the propagation directions of multiple incident plane waves, while temporal modulation introduces different frequency shifts so that the reflected waves become mutually incoherent and their powers add without phase control. The operating principle is depicted in Figure 23 [97], where several plane waves with the same initial frequency arriving from different angles are mapped into a single output direction, each with a distinct frequency offset. In particular, the metasurface is modeled as a grounded impedance sheet with a spatiotemporally modulated capacitance

(66) $$C\left(z,t\right)=\sum_{m}c_{m}e^{j(m\beta_{M}z-m\omega_{M}t)},$$

and analyzed using Floquet mode matching. Treating each Floquet harmonic as a port, a rigorous multiport formulation clarifies why perfect phase-independent combining is impossible in linear time-invariant systems and how time modulation circumvents this barrier. Photon-flux conservation, expressed through the Manley–Rowe relations, provides a precise combining efficiency metric.

Design examples demonstrate combining efficiencies approaching unity for two input beams redirected into a single-output channel, with negligible parasitic reflections and with evanescent modes excited locally at the surface to enforce boundary conditions without carrying net power. The metasurface remains at a finite bandwidth, which is sufficient for broadband pulses provided the spectral content lies within the modulation band. Increasing the modulation depth broadens the operational bandwidth at the expense of quality factor trade-offs. The concept naturally generalizes to multiple input ports by enriching the spatiotemporal modulation profile and is implementable from microwaves using loaded varactors to optics using electro-optic or Kerr-based index modulation.

These investigations established beam control in time-varying media as a unified spatiotemporal signal-processing paradigm. Temporal aiming enables deterministic, real-time steering and routing without spatial phase engineering, while spatiotemporal metasurfaces enable phase-insensitive beam combining by deliberately decorrelating spatial channels in frequency. Both approaches demonstrate how time modulation extends classical beam-forming and combining concepts into a four-dimensional design space that merges spatial and spectral degrees of freedom under programmable temporal control.

### 4.8. Diffusive Spatiotemporal Metamaterials

Beyond wave propagation, spatiotemporal modulation also enables unprecedented control over diffusive transport processes. By jointly programming material parameters in space and time, it becomes possible to bias, trap, and route diffusive quantities such as charge, heat, or chemical species without any physical flow. A representative formulation is described in [98], which establishes a general methodology for controlling diffusion through the joint space–time modulation of a medium’s conductivity and capacity. Starting from a one-dimensional Fick transport equation with spatiotemporally varying parameters, this work performs a homogenization analysis in the fast-modulation limit and obtain an effective advection diffusion equation governing the time-averaged quantity $\tilde{q}\left(x,t\right)$,

(67) $$\partial_{t}\tilde{q}=\tilde{D}\;\partial_{xx}\tilde{q}-v\;\partial_{x}\tilde{q},$$

where the effective diffusion and drift coefficients are

(68) $$\tilde{D}=\sigma_{0}g_{0}+\frac{1}{2}\sigma_{m}g_{m}\cos\phi ,\;\;v=\frac{1}{2}k\;\sigma_{m}g_{m}\sin\phi .$$

Here, σ and *g* denote the conductivity and storage (capacity) modulations, respectively, *k* is the modulation wavenumber, and ϕ is the relative phase between the two modulations. Phase ϕ directly sets both the magnitude and the sign of the emergent drift velocity *v*, thereby biasing diffusion to accumulate or evacuate conserved quantities toward selected regions. Importantly, this transport bias is effective rather than convective and arises even though the underlying medium remains physically stationary. The validity of the homogenized description requires the modulation period to remain short compared with the intrinsic diffusion time, a condition that is explicitly analyzed and verified.

The theoretical predictions are demonstrated experimentally using a reconfigurable electrical lattice composed of a chain of variable capacitors and mechanically controlled switches that implement the prescribed space–time modulation. By tuning the phase offset ϕ, the system exhibits pronounced asymmetric diffusion. For one phase choice, charge accumulates at a boundary and produces a large, measurable voltage enhancement. For the opposite phase, the source becomes effectively isolated and the downstream region remains largely unperturbed. The study further introduces engineered domain boundaries where the sign of *v* changes, giving rise to diffusion traps that generate localized accumulation and anti-traps that enforce depletion. By slightly detuning the modulation frequency or wavelength, these trapping regions can also be made mobile, creating a drifting bias profile that is often described in terms of a beat velocity of the modulation envelope. Numerical extensions to two dimensions reveal the formation of corner traps and programmable accumulation interfaces. Overall, this work establishes spatiotemporal diffusive metamaterials as a new platform for biasing, trapping, and routing diffusive quantities through pure parameter modulation, with direct implications for electronic, thermal, and electrochemical transport.

Collectively, the studies reviewed in this section demonstrate that temporal and spatiotemporal modulation elevates wave control far beyond the limits of linear time-invariant systems. Across platforms and frequency ranges, time variation enables functionalities that are either fundamentally unattainable or severely constrained in static media, including broadband magnet-free nonreciprocity, isolation through unidirectional frequency transitions, absorption beyond classical bounds via temporal switching, programmable frequency translation, temporal impedance matching, parametric amplification driven by momentum band gaps, temporal cloaking, real-time beam steering and combining, and biased diffusion without physical flow. A central unifying theme is that energy exchange is no longer governed solely by material loss and external sources but is actively mediated by the modulation itself, which reshapes dispersion, conservation laws, and mode coupling pathways. These applications establish temporal metamaterials as a versatile platform for reconfigurable, broadband, and nonreciprocal wave engineering across electromagnetics, photonics, acoustics, and diffusion-dominated systems.

## 5. Numerical and Semi-Analytical Methods

The rapid development of time-varying and space–time-modulated media has been accompanied by the need for robust numerical and semi-analytical tools capable of capturing nonstationary dispersion, frequency conversion, and causal scattering [99,100]. Conventional frequency domain-solvers are often inadequate in this setting, since temporal modulation intrinsically couples multiple harmonics and breaks time-invariance. Consequently, specialized time-domain and Floquet-based methods have been developed to address both volumetric time-varying materials and zero thickness, space–time-modulated metasurfaces. This section surveys representative advances ranging from generalized FDTD formulations for bulk media, to GSTC-based solvers for modulated sheets, and to fast semi-analytical harmonic-domain techniques that rigorously retain temporal dispersion.

A foundational extension of the typical FDTD method to spatiotemporally varying media was presented in [101]. Specifically, Maxwell’s equations are formulated in cylindrical coordinates for materials whose permittivity and conductivity depend on both space and time through the constitutive laws D=ε(r,t)E and J=σ(r,t)E. A centered in space and centered in time discretization was employed, with linear interpolation used to remove minor staggering inconsistencies at cell interfaces. The method was applied to pulsed scattering from a perfectly conducting cylinder placed inside a cylindrical waveguide, with the incident TM_01_ mode constructed as a Fourier superposition consistent with $E_{z}\left(z,r,t\right)=J_{0}\left(pr\right)F\left(z\pm ct\right)$. Boundary fields were enforced through linear extrapolation at computational edges and perfect conductor conditions on the metallic surface. Basically, the two-dimensional Courant condition $\Delta t/\Delta s<1/\sqrt{2}$ ensures numerical stability as (Δt,Δs)→0. The solver was validated in three representative scenarios, namely propagation in free space, propagation in a homogeneous yet time-varying lossy medium with σ(t)=0.16ct mho/m, and propagation through a moving ionization front-modeled by $\sigma \left(z,t\right)=\sigma_{0}\left(t\right)f\left(z-ct\right)$. In all cases, the algorithm accurately captured pulse deformation, current accumulation on conductors, induced attenuation and dispersion, and energy extraction by time-varying losses. The work provided one of the earliest practical finite-difference time-domain toolkits for scattering formulated as an initial value problem in media with both spatially and temporally varying material parameters.

**Figure 24:**
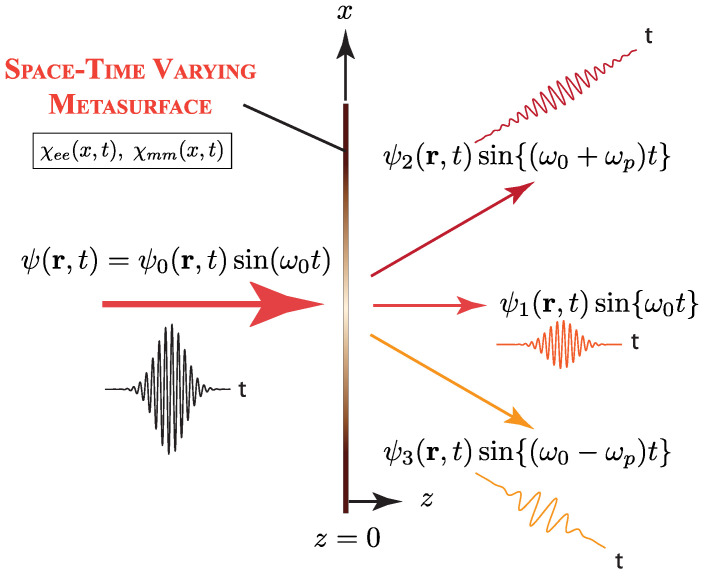
Illustration of spacetime Huygens’ metasurface ($\omega_{p}$ is the pump frequency) [102].

On the other hand, Ref. [102] develops a rigorous time-domain solver for zero-thickness Huygens metasurfaces with electric and magnetic responses modulated in time or in both space and time. The metasurface, shown in Figure 24, is modeled through generalized sheet transition conditions (GSTCs) coupled with Lorentzian surface susceptibilities extracted from full-wave unit cell simulations. By mapping the practical permittivity modulation of dielectric resonators onto time-varying Lorentz parameters, the modulation is recast as a controlled variation in the resonance frequencies in $\chi_{ee}$ and $\chi_{mm}$, yielding a compact and fully causal surface model. On this basis, Ref. [102] formulates a state space representation that advances the reflected and transmitted fields together with auxiliary polarization variables and is explicitly integrated in time on a standard Yee grid. The solver accurately predicts temporal harmonic generation under pure time modulation and the angular separation of harmonics when a spatial phase is added, in agreement with Floquet theory and Fourier-domain analysis. The core numerical engine is governed by

(69) $$C\;\frac{dV}{dt}+G\left(t\right)\;V=E\left(t\right),$$

where V collects the field and polarization states, C arises from the GSTCs, and G(t) encodes the time-varying Lorentz parameters. The formulation accommodates multi-resonant dispersive responses and scales efficiently across frequency ranges.

**Figure 25:**
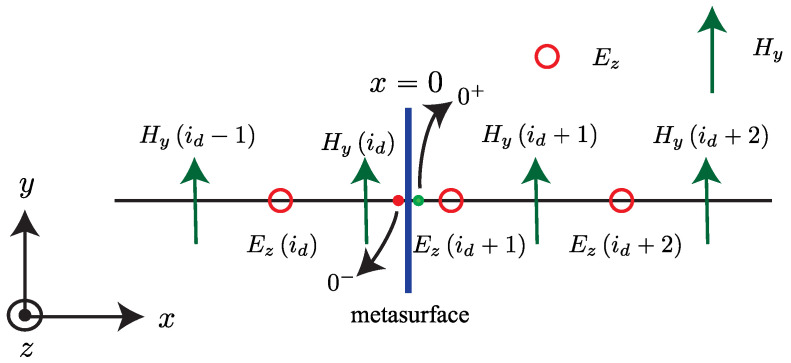
Illustration of a 0-D metasurface. The filled red and green circles represent electric and magnetic virtual nodes just before and just after the metasurface [103].

A more general GSTC-FDTD formulation capable of handling space-, time-, and field-dependent metasurfaces was presented in [103]. The key concept is to insert the metasurface as a “virtual node” between standard Yee grid points and enforce the GSTCs at that node, as illustrated in Figure 25. This preserves the standard FDTD structure away from the sheet while capturing electric and magnetic discontinuities with full bianisotropic generality.

Starting from the time-domain GSTCs

(70) $$\Delta H_{y}=\varepsilon_{0}\;\frac{d}{dt}\;\left[\chi_{ee}^{zz}E_{z,\mathrm{av}}\right],\;\;\Delta E_{z}=\mu_{0}\;\frac{d}{dt}\;\left[\chi_{mm}^{yy}H_{y,\mathrm{av}}\right],$$

explicit update equations are derived for the virtual fields adjacent to the sheet. When higher-order time derivatives appear in the GSTCs, auxiliary differential equations are introduced, whereas first-order cases remain fully explicit. The method was validated through five benchmarks, including time-modulated harmonic generation, graphene sheets, dispersive partially absorbing metasurfaces, and reflectionless space–time sheets. In all cases, the numerical results agreed closely with the analytical or literature solutions while avoiding volumetric meshing of thin layers.

Moreover, a fast semi-analytical alternative retaining full temporal dispersion was derived in [104]. The metasurface is modeled through GSTCs combined with Lorentz-oscillator surface susceptibilities whose parameters are modulated in space and time. Expanding all quantities into Bloch–Floquet harmonics yields a finite linear matrix system that directly returns the amplitudes of all scattered harmonics. The angular direction of each harmonic obeys the kinematic relation

(71) $$\sin\theta_{m,n}=\frac{\sin\theta_{0}+m\;\beta_{p}/k_{0}}{1+n\;\omega_{p}/\omega_{0}},$$

which jointly captures spatial diffraction and temporal frequency conversion. The formulation rigorously includes surface dispersion through Lorentz response, and reduces to dispersion-free models in appropriate limits.

**Figure 26:**
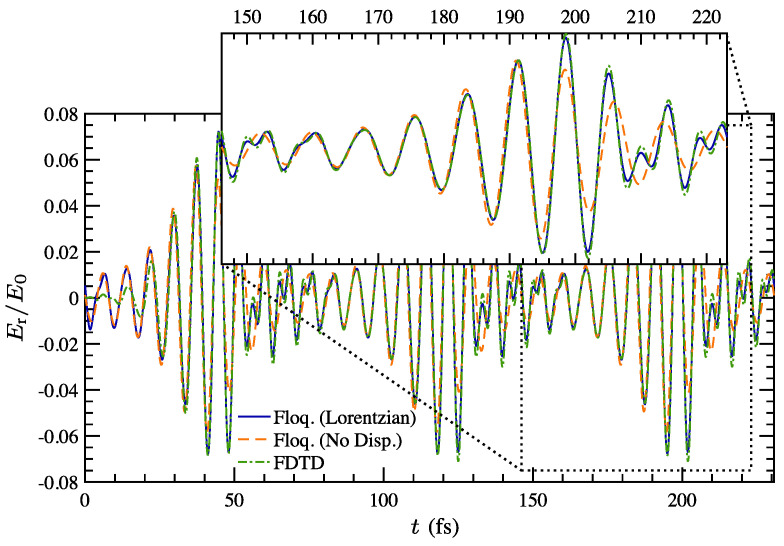
Reflected field at $z=0^{-}$ from a uniform space–time-modulated surface with an incident wave that is far from the resonant frequencies of the surface susceptibilities [104].

**Figure 27:**
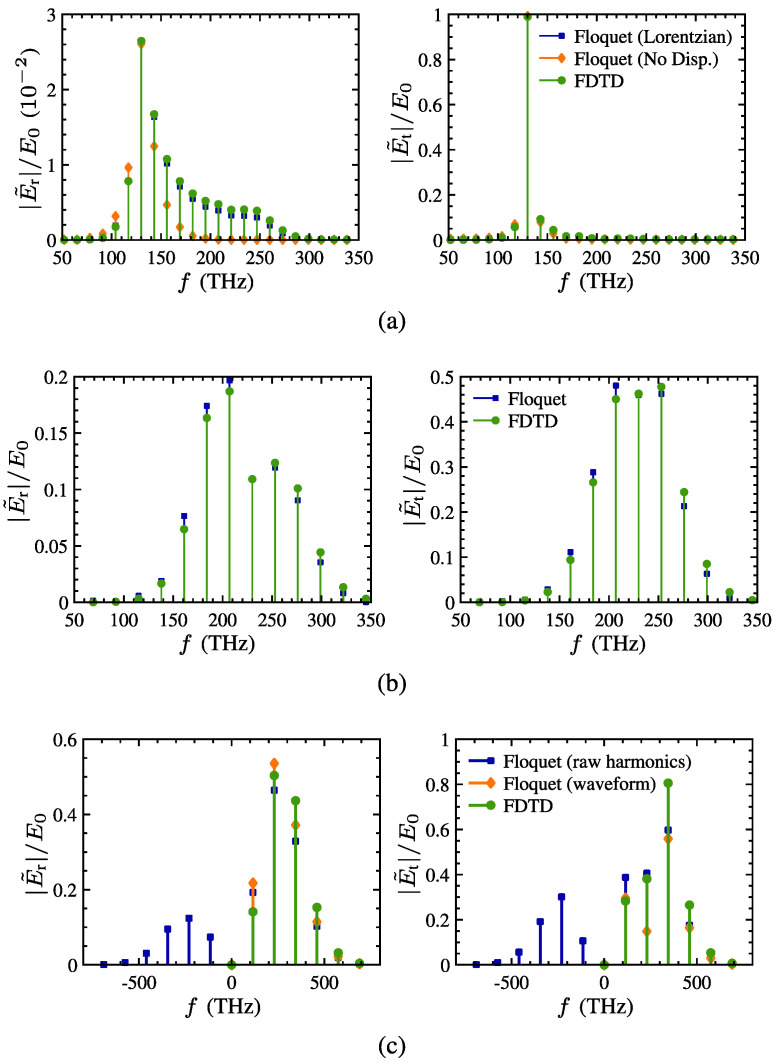
Three cases of a uniform time-modulated metasurface with $\zeta_{\left\{ee,mm\right\}}^{\left\{yy,xx\right\}}\left(t\right)=\zeta_{0,\{ee,mm\}}^{\left\{yy,xx\right\}}\left(t\right)\left[1+\Delta_{e,m}\cos\left(\omega_{p}t\right)\right]$. Modulation is (**a**) 0.2, (**b**) 0.3, and (**c**) 0.5 [104].

Validation was performed for pure spatial modulation, pure temporal modulation, and combined space–time modulation. For purely spatial modulation, harmonic spectra matched frequency-domain and full-wave solvers. For pure temporal modulation, Floquet predictions incorporating Lorentz dispersion agreed with the finite-difference time-domain method and revealed the large errors produced by dispersion-free models near resonance, as shown in Figure 26 [104]. For fully space–time-modulated sheets, generalized space–time *S*-parameters quantified reciprocity and confirmed that standing-wave modulation is reciprocal, whereas traveling-wave modulation breaks Lorentz reciprocity and enables asymmetric frequency conversion and beam steering, as further illustrated in Figure 27 [104]. The method is computationally light, accurate for ultrathin dispersive sheets, and extensible to arbitrary wavepacket excitation via plane-wave and Gaussian decompositions.

In summary, the preceding methods lead to a comprehensive computational tool for analyzing space–time-modulated media across vastly different physical scales and modeling abstractions. Volumetric FDTD formulations enable the direct time-domain simulation of nonstationary bulk materials with full waveform fidelity, while GSTC-based solvers provide numerically efficient access to zero-thickness metasurfaces with electric, magnetic, and bianisotropic responses. Complementing these brute-force approaches, semi-analytical Floquet and Lorentz methods offer the fast yet rigorous prediction of harmonic spectra, reciprocity breaking, and beam steering under space–time modulation, while correctly accounting for surface dispersion. These tools provide the numerical backbone of modern space–time metamaterial research and directly support both theory- and application-oriented design.

## 6. Experimental Demonstrations and Prototypes

This section surveys representative experimental realizations that translate the theoretical concepts of temporal and spatiotemporal modulation into working hardware platforms, with emphasis on microwave, RF, and integrated photonic implementations. These prototypes demonstrate that key functionalities, such as frequency conversion, nonreciprocity, duplexing, radiation control, and impedance matching, enabled by time-varying media, are practically achievable using standard circuit, metasurface, and electro-optic technologies. The reviewed experiments span guided-wave structures, transmission-line mixers, and metasurfaces operating in unbounded space, and they validate the promise of time-varying media as compact, magnet-free, and reconfigurable functional devices. In addition, selected experimental platforms that achieve related functionalities through material reconfiguration, structural interference, or disorder are discussed to provide context on robustness, scalability, and fabrication considerations relevant to their practical deployment.

A representative experimental platform is provided by Taravati and Caloz [105], who introduced a two-port leaky-wave transceiver that simultaneously performs mixing, duplexing, and radiation by applying a traveling spacetime modulation to a microstrip transmission line. A harmonic modulation propagating along the +z direction imparts synthetic momentum and frequency to guided waves, producing oblique space–time transitions that are inherently nonreciprocal, as forward transitions exist while backward ones are suppressed by the dispersion. The modulation is modeled as

(72) $$\varepsilon \left(z,t\right)=\varepsilon_{e}\;\left[\;1+\delta_{m}\cos\;\left(\omega_{m}t-\beta_{m}z\right)\right],$$

which yields the Floquet expansion

(73) $$E\left(z,t\right)=\sum_{n=-\infty }^{\infty }E_{n}\;e^{j\omega_{n}t}\;e^{-(\alpha_{n}+j\beta_{n})z},\;\omega_{n}=\omega_{0}+n\omega_{m},\;\beta_{n}=\beta_{0}+n\beta_{m}.$$

In the uplink state, a guided signal at $\omega_{0}$ is converted to $\omega_{1}=\omega_{0}+\omega_{m}$ and transitions into a fast leaky harmonic that radiates at the angle

(74) $$\theta_{1}=\sin^{-1}\;\left(\frac{c\;\left(\beta_{0}+\beta_{m}\right)}{\omega_{0}+\omega_{m}}\right).$$

**Figure 28:**
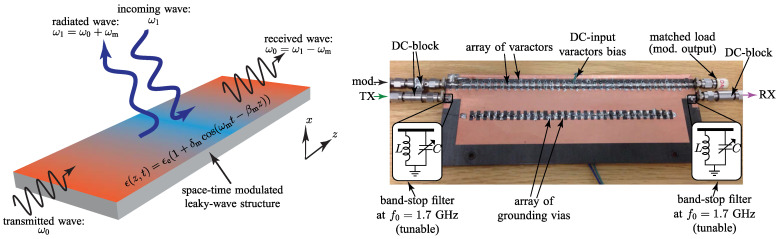
Illustration of a periodically space–time-modulated leaky-wave structure (**left**) and realization of the fabricated prototype (**right**) [105].

The downlink reverses the process for an incoming beam at $\omega_{1}$, which is down-converted back to $\omega_{0}$ and routed to the receiver port. Figure 28 (left) details the architecture and leaky transitions induced and guided by the traveling modulation [105].

A practical microstrip realization implements the spatiotemporal permittivity using a dense array of varactors driven by a guided even mode, while radiation occurs on a higher-order odd leaky mode and suppresses unwanted even-mode leakage. The prototype, operated at $f_{0}=1.7$ GHz with $f_{m}=0.18$–0.30 GHz, demonstrates uplink radiation at $f_{1}=f_{0}+f_{m}$ with a measured main beam near $\theta_{1}\approx 4^{\circ }$, downlink reception with down-conversion to $f_{0}$, and isolation of about 31.5 dB between TX and RX ports. Sweeping $f_{m}$ steers the radiated beam in agreement with the theoretical dispersion relation. Analytical dispersion models, approximate closed-form solutions, full-wave simulations, and measurements agree closely across the operating band. Figure 28 (right) shows a photograph of the fabricated prototype, highlighting the varactor array, bias network, and leaky aperture.

An alternative experimental approach to clean frequency translation is presented in [106], which proves that spatial aperiodicity, when superimposed on temporal periodic modulation, yields pure frequency conversion while suppressing undesired spurious harmonics. The principal concept is to engineer a transmission line whose effective ε(z) and μ(z) vary aperiodically in space, while a uniform pump imposes a sinusoidal modulation in time. The resulting aperiodic photonic band gaps block the growth of all harmonics except the target $\omega_{1}=\omega_{0}+\omega_{p}$. Inside the modulated section, the fields admit a time Bloch–Floquet expansion,

(75) $$E\left(z,t\right)=\sum_{n\in Z}E_{n}\left(z\right)\;e^{-i(\omega_{0}+n\omega_{p})t},\;H\left(z,t\right)=\sum_{n\in Z}H_{n}\left(z\right)\;e^{-i(\omega_{0}+n\omega_{p})t},$$

and the harmonic envelopes evolve according to the coupled first-order matrix equations derived from Maxwell’s equations with spatiotemporally varying constitutive parameters. This formulation reveals how the aperiodic gaps clamp all undesired channels n≠1 while allowing for robust energy transfer from n=0 to n=1.

**Figure 29:**
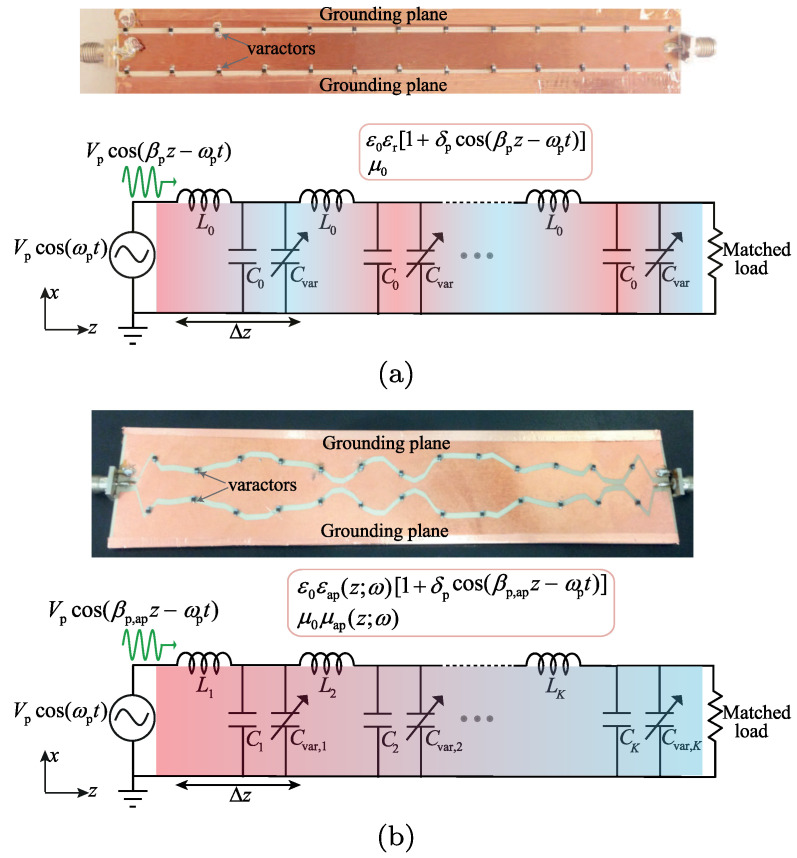
(**a**) Equivalent circuit model of the fabricated periodic space–time-modulated mixer using an array of varactors, and (**b**) equivalent circuit model of the fabricated aperiodic space–time-modulated mixer using an array of varactors [106].

For synthesis, the line is discretized into deep-subwavelength sections whose target passbands and stopbands are implemented using an ABCD matrix cascade. The designed $\varepsilon_{ap}\left(z\right)$ and $\mu_{ap}\left(z\right)$ are realized using a coplanar waveguide backed by a conducting layer and loaded with distributed varactors driven by a traveling pump. Dispersion diagrams confirm tailored stopbands at all undesired $\omega_{0}+n\omega_{p}$ and a clean passband at $\omega_{1}$. The experimental comparison between periodic and aperiodic mixers is decisive. The periodic line generates a forest of spurious tones, whereas the aperiodic line suppresses them and yields a single clean output at $\omega_{0}+\omega_{p}$ with linear input–output scaling and electrical tunability. For illustration, Figure 29 juxtaposes the two hardware implementations [106].

A distinct free-space implementation of temporal frequency translation is reported by Wu and Grbic [107], who realized a transparent metasurface with matched impedance that performs serrodyne frequency conversion at *X*-band by imposing a near-linear sawtooth temporal phase. The authors first analyze an ideal time-modulated refractive index n(t) whose permittivity and permeability satisfy $\mu_{r}\left(t\right)/\varepsilon_{r}\left(t\right)=1$, thereby enforcing instantaneous impedance matching. Expanding fields and material parameters into temporal harmonics leads to the eigenproblem

(76) $$c^{2}\;\kappa^{2}A=QA,$$

where κ is the supported wavenumber and A collects the harmonic amplitudes. The entries of Q are set by the Fourier coefficients of $n^{2}\left(t\right)$ and the modulation frequency $\omega_{s}$. This formulation clarifies how a sawtooth modulation progressively transfers energy from $\omega_{0}$ to $\omega_{0}+\omega_{s}$ as the wave propagates, yielding serrodyne translation with strong suppression of image tones in the low-$\omega_{s}$ regime.

The experimental realization employs two transparent, tunable phase-shifting metasurfaces cascaded and driven by optimized sawtooth bias waveforms. Each metasurface implements a co-tuned electric and magnetic phase shifter using varactors, ensuring impedance matching throughout the modulation cycle. In the small-variation regime, the output is described by

(77) $$E_{out}\left(t\right)=A\left(t\right)e^{j[\omega_{0}t+\phi \left(t\right)]},$$

and when A(t) is nearly constant and $\phi \left(t\right)\approx \omega_{s}t$, the ideal translated spectrum becomes

(78) $$E_{\mathrm{out}}\left(\omega \right)\;=\;2\pi E_{0}\;\delta \;\left(\omega -\omega_{0}-\omega_{s}\right).$$

**Figure 30:**
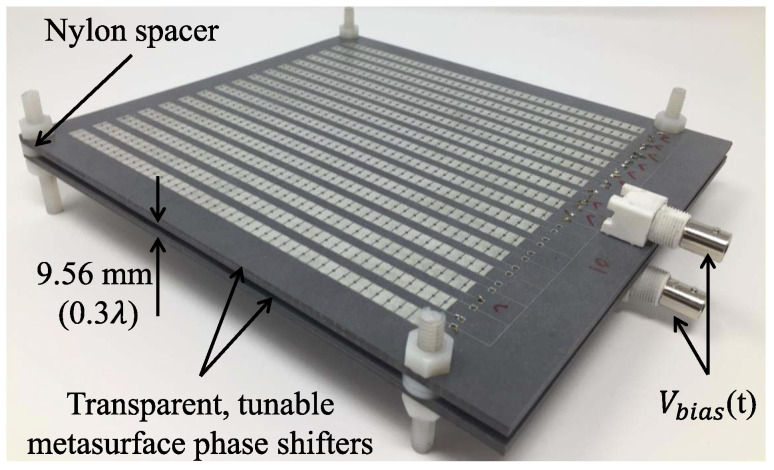
Illustration of the fabricated serrodyne frequency translator [107].

The prototype operates at $f_{0}\approx 10$ GHz with $f_{s}\approx 1$ MHz and incorporates 5712 varactors across two metasurfaces. Gaussian beam measurements in free space show a clean translated tone at $f_{0}+f_{s}$ with ∼5.3 dB conversion loss, ∼12 dB sideband suppression, and a usable bandwidth of about 0.8 GHz. Figure 30 presents the fabricated translator and biasing network [107], rendering transparent, time-modulated metasurfaces as compact, magnet-free frequency translators with strong suppression of spurious harmonics.

Recent experimental advances have also leveraged high-speed electro-optic material platforms to implement spatiotemporally modulated nonreciprocal photonic devices. Specifically, traveling-wave electro-optic modulation on thin-film lithium niobate has enabled broadband, magnet-free optical isolation with low insertion loss and large isolation ratios, demonstrating the practicality of time-modulated nonreciprocity at optical frequencies [108]. In parallel, chip-integrated meta-structured waveguides developed through forward and inverse design methodologies have provided compact and scalable architectures for implementing complex modulation and mode-engineering strategies [109]. These approaches highlight the growing maturity of integrated platforms capable of supporting time-varying and spacetime-engineered photonic functionalities.

In addition to time-modulated and metastructured approaches, recent progress in the heterogeneous integration of magneto-optical materials has enabled low-loss, on-chip nonreciprocal photonic devices without requiring subwavelength micro-structuring. Wafer-bonded magneto-optical platforms based on garnet materials have demonstrated robust isolation and circulation in silicon photonics, and have proven particularly attractive for photonic in-memory computing and laser-integrated systems [110]. These material–intrinsic approaches offer a comparatively simple implementation route for certain nonreciprocal functionalities, albeit with trade-offs related to magnetic biasing, integration complexity, and limited dynamic reconfigurability. As such, magneto-optical platforms should be viewed as complementary to space–time-modulated and programmable meta-structures, addressing different application regimes and system-level requirements.

Beyond RF/microwave prototypes based on electronically driven spatiotemporal modulation, recent work has also demonstrated experimentally mature material-state reconfigurable metasurfaces at optical/IR wavelengths [111]. For instance, Zhou et al. reported a dynamically tunable metal–semiconductor–metal metasurface incorporating the phase-change material Sb_2_Se_3_, enabling switching between multispectral camouflage and radiative-cooling operating states. In the camouflage state, the structure suppresses LiDAR backscatter at 1064 nm while reducing MWIR/LWIR thermal signatures, whereas the alternate state supports enhanced passive cooling. Although this adaptivity relies on discrete phase transitions rather than continuous temporal modulation, it provides an instructive prototype of multifunctional, materials-integrated dynamic metasurfaces relevant to practical fabrication constraints and system deployment.

Recent experimental progress has also demonstrated moiré-induced dispersion engineering and flat-band formation in acoustic metasurfaces through geometric twisting rather than explicit temporal modulation. Fan et al. [112] reported an acoustic twisted heterobilayer metasurface composed of two dissimilar lattice geometries, where interlayer rotation enables a controllable topological transition between hyperbolic and elliptical dispersion regimes. At a specific acoustic “magic angle”, strong interlayer hybridization gives rise to an exceptionally flat band, supporting highly collimated, low-diffraction sound propagation. In particular, both simulations and experiments confirmed that the resulting wave transport remains remarkably robust against line defects and structural disorder, highlighting the practical viability of moiré-based metasurface platforms for defect-tolerant wave manipulation.

Robust resonant phenomena have also been demonstrated in photonic platforms exploiting higher-order topological protection. Peng et al. [113] proposed a cavity-waveguide coupled system based on a second-order topological photonic crystal with elliptical air holes, where a topological corner-state cavity is coherently coupled to a topological edge-state waveguide. This configuration supports an ultrasharp asymmetric Fano resonance with an exceptionally high quality factor exceeding $10^{8}$, enabled by strong field localization and reduced radiation losses. Importantly, numerical investigations showed that both the resonance lineshape and the quality factor remain largely unaffected by structural defects and disorder, underscoring the intrinsic robustness of topological cavity-waveguide systems. Although the reported robustness originates from static topological protection rather than temporal modulation, such results provide a valuable benchmark for defect-tolerant resonant devices and highlight complementary strategies to achieve robustness in metamaterial-inspired platforms.

An alternative route to achieving sharp and tunable spectral responses in compact photonic systems relies on engineered structural interference rather than explicit temporal modulation. In this context, an indirectly coupled double-nanobeam-cavity photonic molecule integrated with a waveguide Fabry–Pérot resonator has been shown to enable precise control over dual Fano resonance line shapes [114]. Continuous tuning between dual Lorentzian and strongly asymmetric dual Fano responses is achieved by adjusting the geometric phase accumulated in the waveguide section, resulting in pronounced improvements in extinction ratio, slope rate, and group delay. Implemented on a silicon-on-insulator platform, this approach combines an ultracompact footprint with fully passive operation, illustrating a fabrication-compatible strategy for realizing high-performance and integrable photonic devices based on interference-driven resonances.

Soft-matter platforms provide a complementary pathway toward dynamically reconfigurable and highly integrable electromagnetic functionality by leveraging the controlled modulation of material-order parameters. Within this framework, a nematic liquid–crystal system exhibiting an intermediate polar mesophase (denoted as the Nx phase) has been demonstrated to support periodically modulated unipolar and bipolar polarization orders [115]. Flexoelectric coupling combined with photoalignment techniques enables large-area, defect-free in-plane polar domain engineering with a high degree of design flexibility. As a representative application, a single–micrometer-thick liquid–crystal device was shown to generate both linear and nonlinear perfect vector beams, achieving a comparable performance to cascaded conventional optical components while retaining a compact and integrable form factor. Although the reconfigurability in this case arises from the modulation of material phase and polarization order rather than explicit time-periodic parameter switching, the results underscore the practical maturity of soft-matter-based dynamic metamaterial platforms for miniaturized and multifunctional wave control.

Dynamic metasurface concepts have recently been pushed into the nonlinear optical domain through the use of soft ferroelectric nematic materials. Specifically, photopatterned, ion-doped ferroelectric nematic liquid crystals have been employed to realize reconfigurable nonlinear Pancharatnam–Berry diffractive optics [116]. Spatial control of the polar nematic director field enables programmable nonlinear geometric-phase accumulation during second-harmonic generation, allowing for electrically tunable Raman–Nath diffraction and beam steering across multiple polarization channels. The resulting nonlinear diffraction patterns and intensity profiles can be continuously adjusted using exceptionally low in-plane electric fields, while preserving large-area uniformity and defect-free polarization textures. Although the underlying tunability is governed by electrically driven polarization-order modulation rather than rapid space–time parameter variation, this approach demonstrates a practical and fabrication-compatible route toward dynamically programmable nonlinear metasurfaces based on soft-matter platforms.

From a fabrication perspective, recent breakthroughs have significantly expanded the practical feasibility of complex nanophotonic and metamaterial architectures. Chen et al. [117] reported a wet-chemical etching-assisted, aberration-enhanced, single-pulsed femtosecond laser nanolithography technique, enabling the fabrication of large-area holey nanostructures with feature sizes down to 25 nm, aspect ratios exceeding 10^4^:1, and lateral areas of 10 mm^2^, with demonstrated scalability toward the cm^2^ regime. The method provides exceptional uniformity along the longitudinal direction, high duty cycles, and compatibility with a broad range of crystalline, glassy, and semiconductor materials. Such advances directly address long-standing fabrication bottlenecks and provide a critical technological foundation for realizing large-area, high-performance nanophotonic crystals, chiral metasurfaces, and future dynamically reconfigurable metamaterial platforms.

Recent experimental studies indicate that advanced metamaterial functionalities need not rely exclusively on periodic or highly ordered architectures, and that controlled structural disorder can instead be exploited as a design resource. Using an irregular growth strategy composed of a limited library of unit cells with graded stiffness, static mechanical cloaking and camouflage have been experimentally demonstrated in aperiodic architected materials [118]. These structures exhibit effective invisibility of internal voids as well as mutual camouflage between different defect configurations across a broad range of boundary conditions, with performance confirmed through numerical modeling and 3D-printed realizations. Notably, the methodology requires only a small set of building blocks and maintains functionality in the presence of loading variability and fabrication tolerances. While realized in a mechanical context, these disorder-enabled design principles point toward scalable and resilient metamaterial strategies and provide useful insight for electromagnetic and time-varying metastructures, where strict periodicity and precise parameter control may be difficult to sustain.

A particularly noteworthy experimental demonstration of asymmetric wave transport in a fully linear and passive system was recently reported by Hu et al. [119], who realized a three-port elastic-wave circulator without relying on temporal modulation, nonlinearity, or external biasing fields. The proposed device exploits tailored wave-vector evolution and controlled mode transition in gradient elastic waveguides to enable the exclusive clockwise routing of flexural waves, while suppressing counter-propagating transmission through wave trapping. Experimental measurements on steel-plate prototypes confirmed broadband asymmetric transport, with high contrast ratios and strong robustness against structural defects and fabrication imperfections. Importantly, although the system remains reciprocal in the strict Lorentz sense, this work demonstrates that pronounced circulation-like functionality can be achieved in passive architectures through spatial symmetry breaking and wave-vector engineering, providing a valuable experimental benchmark and a complementary design paradigm alongside time-varying and space–time-modulated metamaterial approaches.

Evidently, the experimental demonstrations reviewed in this section illustrate both the practical viability and the growing diversity of approaches for realizing advanced wave functionalities inspired by space–time modulation concepts. While electronically driven temporal and spatiotemporal modulation provides a direct and physically transparent route to nonreciprocity, frequency translation, duplexing, and dynamic beam control across microwave and optical platforms, complementary strategies based on material-state reconfiguration, structural interference, topological protection, moiré engineering, controlled disorder, and magneto-optical integration highlight alternative pathways toward robustness, scalability, and fabrication compatibility. Importantly, many of these platforms emphasize resilience to imperfections, reduced reliance on precise periodicity, and compatibility with large-area or chip-scale fabrication, addressing key constraints that arise in real-world implementations. Collectively, these experimental advances underscore that time-varying metamaterials should be viewed as part of a broader and increasingly established experimental ecosystem for programmable, magnet-free, and reconfigurable wave control across electromagnetic, acoustic, and mechanical domains.

## 7. Quantum and Ultrafast

Beyond classical wave manipulation, time-varying media also play a central role in quantum and ultrafast photonics, where modulation rates approach or exceed intrinsic optical and electronic time scales [120,121,122,123,124,125]. In this regime, temporal and spatiotemporal perturbations reshape band structures, induce controlled interband transitions, generate synthetic gauge fields, and even motivate new phases of matter related to time-translation symmetry. This section reviews representative contributions that link dynamic electromagnetic media to quantum dynamics, ultrafast material responses, and the emergence of time crystalline behavior.

A fundamental formulation from quantum optics of spatiotemporally driven interband transitions in photonic crystals is given in [126]. By casting Maxwell’s equations into a Schrödinger form, the unperturbed Bloch modes of the crystal play the role of eigenstates, while a time- and space-dependent permittivity perturbation supplies the coupling. The modal amplitudes obey the time-dependent perturbation equation

(79) $$i\frac{da_{m}}{dt}=\sum_{n}V_{mn}\left(t\right)a_{n}e^{i\left(\omega_{m}-\omega_{n}\right)t},$$

with matrix elements $V_{mn}\propto \int d^{3}r\;\left[\delta \varepsilon \left(r,t\right)/\varepsilon^{2}\right]\;D_{m}^{*}\left(r\right)\cdot D_{n}\left(r\right)$. Because the coupling operator is non-Hermitian under the electromagnetic inner product, the dynamics is not strictly norm-conserving, in contrast to closed quantum two-level systems, yet resonant two state and multistate exchanges are still captured accurately. Phase matching is generalized by the crystal lattice so that a Bloch perturbation with wave vector k couples states $k_{m}$ and $k_{n}$ whenever $k_{m}+k_{n}-k=G$ for some reciprocal lattice vector G, with the symmetry of the Bloch fields governing whether the overlap integral vanishes.

This theory is validated via FDTD simulations in a two-dimensional square lattice photonic crystal of high-index rods. A resonant, phase-matched perturbation drives an $X_{1}\to M_{2}$ transition, and the computed modal populations follow the reduced two-state solution quantitatively, including the predicted scaling of the transition time with perturbation strength. The final state lies at a band edge, producing a stationary standing-wave field. A second example demonstrates three-state dynamics $M_{1}↔X_{3}↔M_{5}$, with excellent agreement between full simulations and a three-mode model. These examples illustrate indirect interband conversion, stationary-mode generation without counter-propagating inputs, and progressive “band climbing” under continued driving. The work highlights how photonic crystals enable programmable temporal pathways and frequency conversion sequences that have no direct analog in homogeneous media and differ sharply from electronic interband transitions constrained by Fermi filling and relaxation.

Another perspective on time-translation symmetry is developed in [127], which examines whether a closed quantum system can spontaneously break continuous time-translation symmetry analogously with how ordinary crystals break spatial translation symmetry. After reviewing intuitions based on the Heisenberg equation for energy eigenstates, the work clarifies how motion in the ground state can arise without contradiction and formulates a concrete many-body model exhibiting time-translation symmetry breaking (TTSB) in the thermodynamic limit. As a preliminary example, a charged particle on a ring threaded by fractional flux supports a persistent current in its ground state yet does not break time symmetry, which helps isolate the essential ingredients required for genuine TTSB.

The central construction considers many weakly attracting ring particles described at the mean-field level by a nonlinear Schrödinger equation on the circle with flux α,

(80) $$i\;\partial_{t}\psi \left(\phi ,t\right)=\frac{1}{2}{\left(-i\;\partial_{\phi }-\alpha \right)}^{2}\;\psi \left(\phi ,t\right)-\lambda \;{\left|\psi \left(\phi ,t\right)\right|}^{2}\;\psi \left(\phi ,t\right)\;.$$

Above a critical interaction strength, the ground state solution forms a localized density “lump” that moves uniformly around the ring, thereby breaking continuous time-translation symmetry while remaining the lowest-energy state within a sector of broken symmetry. The analysis shows that TTSB appears only in the infinite-system limit, where symmetry-related states become orthogonal and local measurements select one sector. The work also addresses physical concerns such as radiation, locality, and stability, and outlines model variations that preserve the essential symmetry breaking physics. In addition, the concept of “imaginary time-crystals” is introduced, where dominant finite temperature path integral configurations become periodic in imaginary time and thermodynamic quantities inherit a periodicity in inverse temperature. These ideas establish a logically consistent mechanism for spontaneous time-translation symmetry breaking in quantum systems and clarify the role of symmetry sectors, observability, and the thermodynamic limit.

Furthermore, Fang et al., in [128], demonstrate a distinct manifestation of temporal modulation in the quantum ultrafast regime, where the phase of a harmonic index modulation acts as a synthetic gauge potential for light, enabling a photonic Aharonov–Bohm effect and magnet-free on-chip isolation. Starting from two guided modes of opposite parity in a slab waveguide, the authors derive a coupled-mode system in which the modulation phase ϕ enters as a controllable Peierls phase,

(81) $$i\;\frac{d}{dz}\left(\begin{array}{c} a_{1}\\ a_{2} \end{array}\right)=\left(\begin{array}{cc} 0 & C\;e^{-i\phi }\\ C\;e^{i\phi } & 0 \end{array}\right)\left(\begin{array}{c} a_{1}\\ a_{2} \end{array}\right).$$

In this technique, changing ϕ realizes an effective gauge transformation on the two-mode photonic manifold, establishing a direct optical analog of the electronic Aharonov–Bohm effect in which photons acquire phase from temporal modulation rather than from a magnetic vector potential.

**Figure 31:**
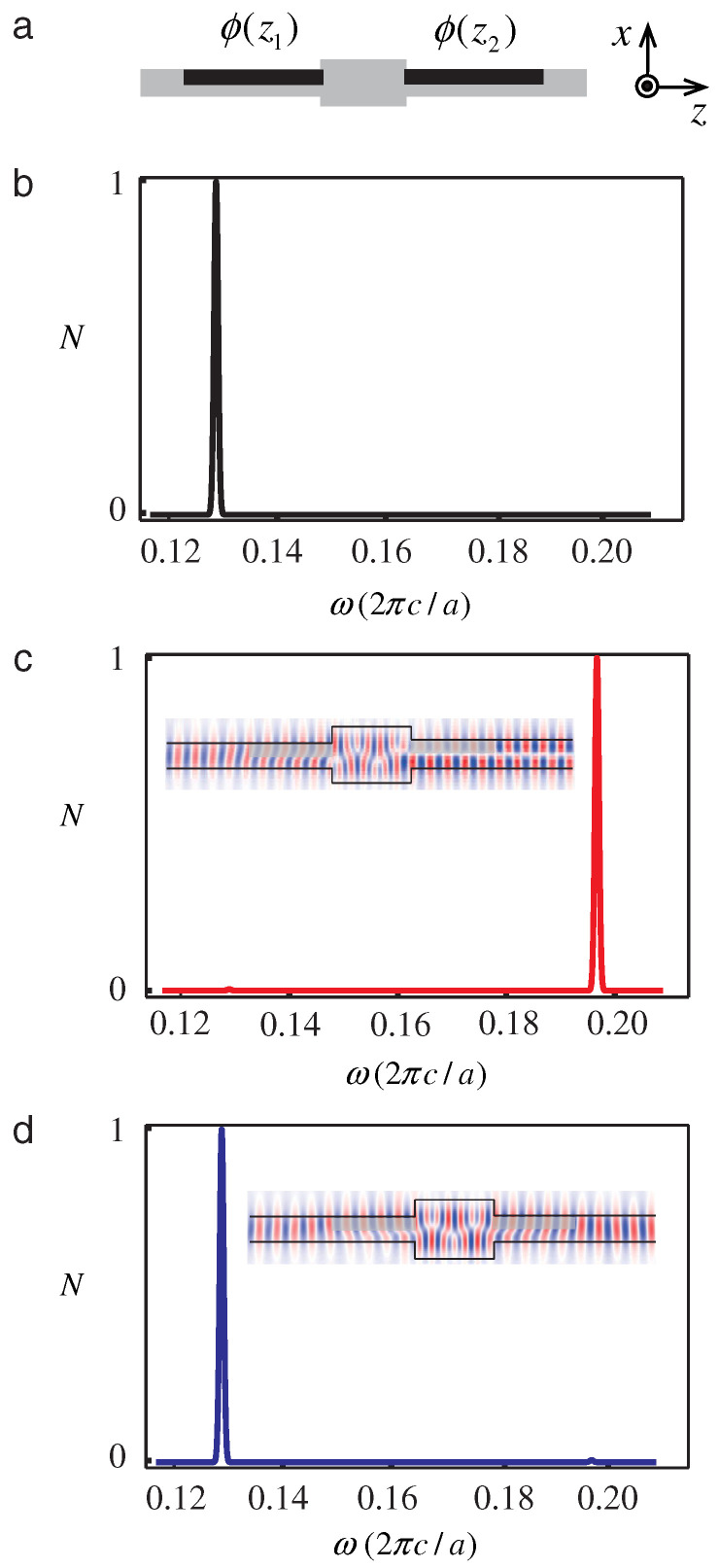
(**a**) Schematic of the waveguide isolator, (**b**) incident photon flux, (**c**) transmitted photon flux when the pulse is incident from the left, and (**d**) transmitted photon flux when the pulse is incident from the right [128].

The interferometer is implemented using two short, spatially uniform modulation sections separated by an unmodulated waveguide. The phase difference $\phi \left(z_{2}\right)-\phi \left(z_{1}\right)$ functions as an effective enclosed gauge flux and generates direction-dependent interference between the stay and convert pathways of the even and odd modes. When $\phi \left(z_{2}\right)-\phi \left(z_{1}\right)=\pi /2$, together with an appropriate converter length and a π/2 propagation phase offset in the intermediate region, the forward-transfer matrix enables complete mode conversion, whereas the backward direction is suppressed, thus breaking Lorentz reciprocity without magnetic bias. Figure 31 shows the silicon waveguide with localized modulators and the measured directional mode conversion, with experimental contrast exceeding 25 dB [128]. Beyond the specific isolator, the gauge phase formulation shows that modulation phase, rather than amplitude, controls nonreciprocal behavior and enables synthetic gauge fields that emulate charged particle phenomena in purely photonic platforms.

Collectively, these studies indicate that time-varying photonic structures extend naturally into the quantum and ultrafast regimes, enabling controlled interband transitions, synthetic gauge fields, and even emergent temporal order. They establish a growing bridge between space–time electromagnetics, quantum dynamics, and condensed-matter concepts, and they point toward future platforms where ultrafast material control and temporal band engineering can be harnessed for quantum information processing, nonreciprocal quantum devices, and photonic systems based in time-crystals.

## 8. Acoustic and Mechanical Time-Varying Media

While space–time modulation was initially developed in electromagnetics, closely related concepts have rapidly emerged in acoustics and mechanics, where temporal control of material parameters can be achieved with high contrast and over wide bandwidths [8,129,130,131,132,133,134]. In these platforms, time-varying stiffness, mass, or wave speed introduces temporal boundaries that reshape dispersion, induce nonreciprocity, and enable time-reversal and frequency-selective transport using purely mechanical means. The works reviewed in this section demonstrate how temporal modulation in hydrodynamic, elastic, and structural media leads to time mirrors, one-way mode conversion, and frequency-selective elastic filtering.

**Figure 32:**
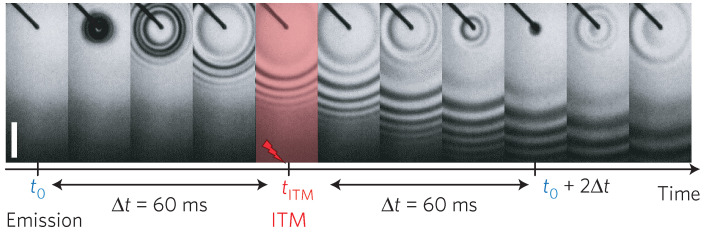
Sequence of images from an ITM experiment with a point source, showing the diverging wave and the wave with with time-reversed wave evolution, which diverges again after focusing back to the source position [135].

An instructive demonstration of temporal wave control is provided by Bacot et al. [135], who realized instantaneous time mirrors (ITMs) in surface water waves dominated by gravity and capillary effects. According to this approach, a sudden, spatially uniform change in the medium’s propagation speed generates a time-reversed wave that refocuses at the original source without requiring any transducer array or record playback process. Experimentally, a vertical jolt of the water bath abruptly modifies the effective wave speed, triggering a backward-propagating wave packet that compensates dispersion and retraces the forward propagation in reverse. The phenomenon is interpreted through a universal framework in which a time disruption decouples the field from its time derivative and acts as a Cauchy source distributed over all the space at the instant of disruption. In that picture, the scalar wave equation acquires an impulsive source term

(82) $$s\left(r,t\right)=\Delta \phi \left(r,t\right)-\frac{1}{c_{0}^{2}}\frac{\partial^{2}\phi }{\partial t^{2}}\left(r,t\right)$$

which explains the broadband, phase-coherent generation of the time-reversed component and its linear scaling with the jolt amplitude. Figure 32 visualizes this process [135], showing a wave that initially diverges outward, is interrupted by the ITM trigger, and then forms a backward converging packet that sharply refocuses at the source before diverging again.

Beyond the phenomenology, the study clarifies conservation and causality at temporal boundaries. Momentum is conserved across the temporal jump, whereas energy is not, so a single temporal interface produces time-refraction and time-reflection components with frequency shifts. Two successive discontinuities act as a time slab whose interference gives rise to the observed refocused packet. The refocused field is proportional to the time derivative of the original field, consistent with the measured π/2 phase relation at the disruption instant and with the bandwidth limits set by the jolt’s rise time. These results connect temporal imaging to classical Huygens–Fresnel reasoning through monopolar Cauchy sources on a time boundary and position ITMs as a broadly applicable route to wave control across acoustics, electromagnetics, elastodynamics, and hydrodynamics.

Nonreciprocity in elastic lattices, induced by traveling temporal modulation, is analyzed in [136]. The authors consider a double-mass lattice, where an inner resonating mass is coupled to an outer mass and its spring stiffness is weakly modulated as a traveling pump

(83) $$\tilde{k}\left(x,t\right)=k+2\delta k\cos\left(q_{m}x-\omega_{m}t\right).$$

**Figure 33:**
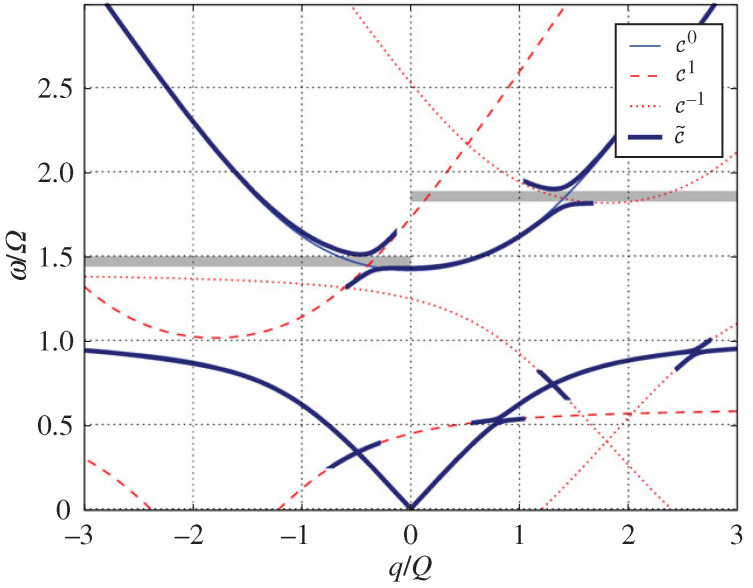
Dispersion curve of the modulated metamaterial [136].

Coupled-mode asymptotics applied to the two-branch dispersion reveal that the pump couples Bloch states satisfying the space–time phase matching conditions $q^{\prime }-q=\pm q_{m}$ and $\omega^{\prime }-\omega =\pm \omega_{m}$. Two interaction topologies emerge. In the locking regime, spacetime modulation opens one-way band gaps that retroconvert waves at shifted frequencies, while in the veering regime, it produces unidirectional mode conversion and amplification over a finite interaction length set by δk, $\omega_{m}$, and $q_{m}$. In forward incidence, an excitation near a coupling point undergoes frequency translation with gain, whereas in reverse incidence, the same excitation largely bypasses the modulation region because the matching conditions fail, thereby breaking reciprocity. Figure 33 visualizes this asymmetry in the calculated dispersion, where pinned coupling points delimit the directional conversion and blocking regions [136].

Beyond the weak-pump limit, the authors explore strong, short-wavelength modulation through homogenization and derive an effective-mass operator that becomes negative over broad low-frequency bands, enabling merged band gaps with no static counterpart. Time-domain simulations of extended lattices corroborate these predictions. Forward excitations exhibit selective frequency translation with gain, whereas backward excitations undergo either reflection or near-transparent transmission with minimal conversion, consistent with the locking veering phase map. This work furnishes a compact mechanical recipe for nonreciprocity based on traveling stiffness waves and points to practical realizations using piezoelectrically programmable resonators.

**Figure 34:**
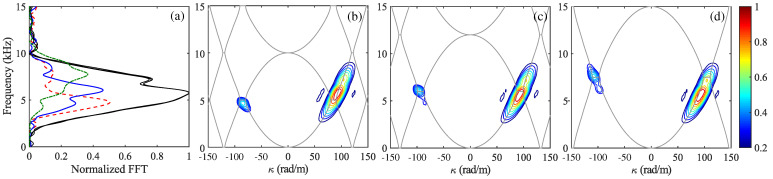
(**a**) Single point FTs show that a broadband input (black solid line) is converted into narrow-band outputs centered at $f_{m}/2$ ($f_{m}=10$ kHz (red curve); $f_{m}=12$ kHz (blue curve); $f_{m}=15$ kHz (green curve)) and (**b**–**d**) normalized two-dimensional FT magnitude [137].

A complementary manifestation of temporal modulation in elastic media is introduced in [137], which demonstrates that periodically modulating the bending stiffness of an elastic waveguide in time produces frequency-periodic dispersion in which branches intersect and merge around half the modulation frequency. For a beam with

(84) $$D\left(t\right)=D_{0}\left[1+\alpha_{m}\cos\left(\omega_{m}t\right)\right],$$

a Floquet expansion of the transverse motion leads to a reduced two-branch model showing that, at the merge points, the real part of the frequency locks to $\omega_{m}/2$ while the imaginary part opens over a finite wavenumber interval, creating a κ-gap with vanishing group velocity. Physically, within this gap, a plane wave excitation cannot propagate through the modulated region, so energy is selectively reflected with a sharply defined frequency at $\omega_{m}/2$. This mechanism is exploited to design a single-port temporal filter formed by a uniform to modulated interface that converts a broadband incident waveform into a narrowband reflected signal. Figure 34 shows the collapse of the reflected spectrum at $\omega_{m}/2$, together with two-dimensional (κ,ω) maps confirming the flat-band reflection mechanism [137]. Finite-difference simulations reproduce the frequency locking and backward concentration of energy along the gap. Experimentally, Ref. [137] implements square-wave stiffness modulation on an aluminum beam using arrays of piezoelectric patches shunted by switchable negative-capacitance circuits. Laser vibrometer measurements confirm narrow-band reflection centered at $\omega_{m}/2$ across several modulation frequencies, with tunable center frequency and reduced output bandwidth relative to the input.

Overall, these studies position acoustic and mechanical platforms as powerful testbeds for space–time media, in which temporal boundaries, traveling modulations, and periodic stiffness control enable time reversal, nonreciprocal transport, and frequency-selective wave manipulation within compact, experimentally verified architectures. They demonstrate that many of the most striking phenomena of temporal electromagnetics have direct and experimentally accessible counterparts in hydrodynamic and elastic systems.

## 9. Artificial Intelligence and Time-Varying Metamaterials

The programmable, high-dimensional mapping naturally induced by space–time modulation has recently enabled a direct interface between temporal metamaterials and artificial intelligence (AI). Rather than using AI solely as a design accelerator, several works now exploit time-varying media themselves as physical learning machines or, conversely, deploy machine learning to synthesize space–time structures with functionalities beyond manual optimization. The studies reviewed here illustrate both directions, namely time-modulated media as analog computing engines and the AI-driven inverse design of highly nonreciprocal space–time platforms.

**Figure 35:**
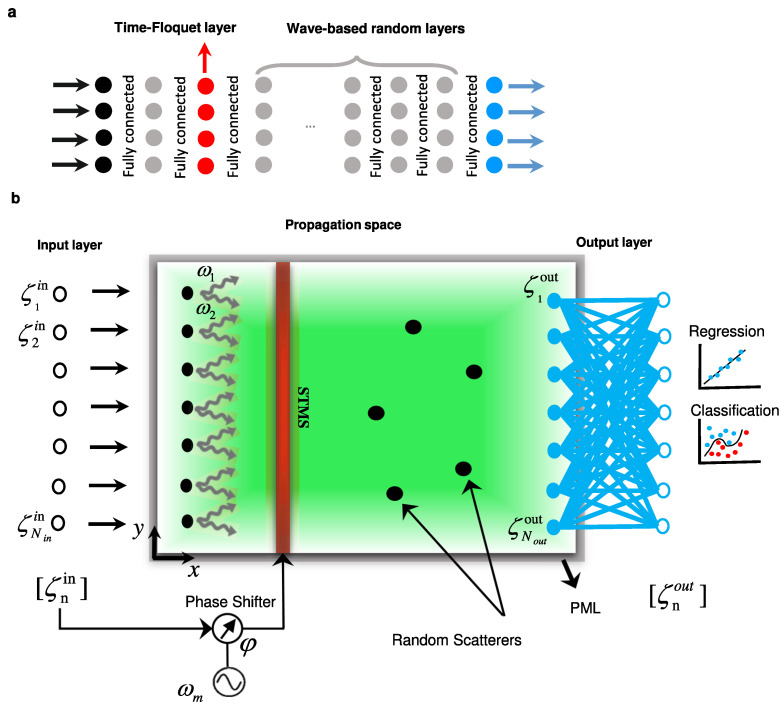
(**a**) Schematic of a neural network, and (**b**) implementation with electromagnetic waves [138].

A first demonstration of time-modulated media as a physical learning substrate is provided by Momeni and Fleury [138], who introduced a wave-based processor formed by a thin time-modulated dielectric slab embedded in a weakly scattering region, as shown in Figure 35. The slab is driven so as to mix two input carriers, $\omega_{1}$ and $\omega_{2}$, at a modulation frequency $\omega_{m}=\left|\omega_{1}-\omega_{2}\right|/2$. A modulation phase delay ϕ imprints an order-dependent phase shift on each Floquet harmonic,

(85) $$R_{\phi }\;\left(\omega_{k}+n\omega_{m}\right)=e^{\;in\phi }\;R_{0}\;\left(\omega_{k}+n\omega_{m}\right),\;T_{\phi }\;\left(\omega_{k}+n\omega_{m}\right)=e^{\;in\phi }\;T_{0}\;\left(\omega_{k}+n\omega_{m}\right),$$

so that the superposition of responses at $\omega_{1}$ and $\omega_{2}$ produces nonlinear interference in the harmonic amplitudes, even though the medium itself remains strictly linear. At the central output frequency $\omega =(\omega_{1}+\omega_{2})/2$, the resulting field amplitude becomes a nonlinear function of the modulation phase once ϕ is made input-dependent. This mechanism, termed nonlinear Floquet entanglement, realizes an effective optical kernel that lifts data into a high-dimensional feature space without relying on material Kerr nonlinearities.

Using this physics, the platform implements both extreme learning machines and a reservoir computer governed by

(86) $$T_{\phi }^{\prime }\left(t+\tau \right)=F\;\left(W_{\mathrm{in}}\;i\left(t\right)+W_{\mathrm{res}}\;v_{h}\;T_{\phi }^{\prime }\left(t\right)\right),$$

where learning occurs only at the final linear readout. Competitive performance is demonstrated on nonlinear regression, the abalone benchmark, parallel image classification, and chaotic time-series forecasting. From a kernel viewpoint, Floquet mixing generates a polynomial feature basis whose richness increases with the number of resolved harmonics. The work establishes time-modulated photonic media as compact, high-speed, and low-power analog processors in which the temporal phase directly programs computation and learning.

**Figure 36:**
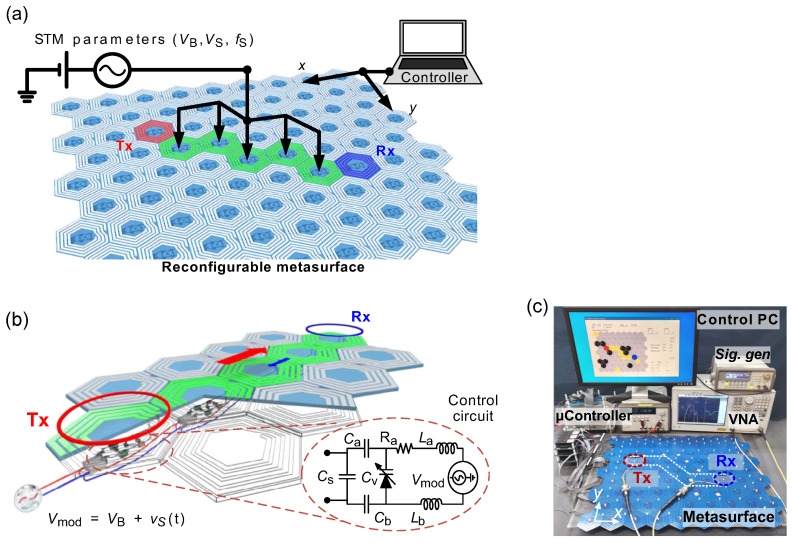
(**a**) Schematic of the metasurface consisting of reconfigurable subwavelength meta-cells, (**b**) detailed control circuit, and (**c**) fabricated space–time metasurface [139].

A complementary use of AI as a design engine for temporal metamaterials is presented in [139], which develops a full machine learning workflow that learns directly from measurements of a space–time-modulated metasurface and then synthesizes new designs with a nonreciprocity exceeding that of the training set. The pipeline, summarized in Figure 36 [139], separates forward modeling from inverse synthesis. A convolutional residual neural network (ResNet) predictor is first trained to map the metasurface geometry and modulation controls (bias, modulation voltage, and modulation frequency) to the measured bidirectional transmission spectra $|S_{21}\left(f\right)|$ and $|S_{12}\left(f\right)|$. Trained on thousands of measured samples, the predictor achieves sub-$10^{-4}$ mean-square error over nearly the entire dataset, making it suitable as a physically informed surrogate model.

Inverse design is then performed using a guided Wasserstein generative adversarial network (WGAN) whose generator is steered not only by the critic but also by the trained predictor through an auxiliary loss term. The degree of nonreciprocity is quantified by

(87) $$D_{\mathrm{TR}}\left(f\right)\;=\;\frac{\left|\;|S_{21}\left(f\right)|-|S_{12}\left(f\right)|\;\right|}{|S_{21,\max}|}\;,$$

and the generator is explicitly biased toward designs that maximize $\max_{f}D_{\mathrm{TR}}\left(f\right)$ via the composite loss

(88) $$L_{\mathrm{GN}}\;=\;-\;L_{f}\;-\;\alpha_{P}\;L_{\mathrm{PR}},\;\;L_{\mathrm{PR}}\;=\;\frac{1}{N}\sum_{i=1}^{N}\max\;\left({\hat{D}}_{\mathrm{TR}}^{\left(i\right)}\right)\;.$$

With this guided strategy, the generated designs exhibit a dramatic shift in nonreciprocity statistics, with the mean asymmetry rising from 0.21 in the measured set to 0.83 in the synthesized population, and many candidates exceeding the maximum observed in the original data. Hardware validation is performed on 9×9 varactor-loaded magneto-inductive metasurfaces with automated VNA-based measurement. The work demonstrates that learned surrogates and adversarial synthesis can efficiently navigate vast space–time design spaces and discover strongly nonreciprocal responses that would be inaccessible to brute-force simulation or manual tuning.

Recent theoretical advances have also focused on improving the modeling and optimization of time-periodic and spatiotemporally modulated systems themselves, particularly in regimes where non-Hermitian effects, radiation leakage, and gain–loss engineering play a dominant role. Reduced-order formulations based on resonant and quasinormal-mode expansions have been extended to Floquet media, enabling the efficient evaluation of scattering responses and parametric interactions in temporally modulated slabs and metastructures [140]. Such approaches provide both physical insight and computational efficiency, making them especially well-suited for inverse design workflows where modulation waveforms, stability constraints, and performance objectives must be optimized simultaneously. These developments help bridge the gap between full-wave time-domain simulations and scalable design methodologies for non-Hermitian and time-Floquet metamaterials.

Additionally, Rossi et al., in [141], reformulated multi-frequency space–time coding (STC) synthesis by targeting harmonic equivalent currents instead of far-field patterns. Under slow, piecewise-constant temporal coding, the scattered field decomposes into discrete harmonic radiation components whose weights $\alpha_{nm}^{\left(v\right)}$ are analytically linked to the applied time sequence through the Fourier series of the rectangular sub-pulses. For an *L*-length code with phase states $\phi_{nm}^{\left(l\right)}$, the harmonic coefficients read

(89) $$a_{nm}^{\left(\nu \right)}=\sum_{l=1}^{L}\frac{1}{L}\;\mathrm{sinc}\;\left(\frac{\pi \nu }{L}\right)\;\exp\;\left[-\;j\;\frac{\pi \nu (2l-1)}{L}\right]\;e^{\;j\;\phi_{nm}^{\left(l\right)}}\;.$$

**Figure 37:**
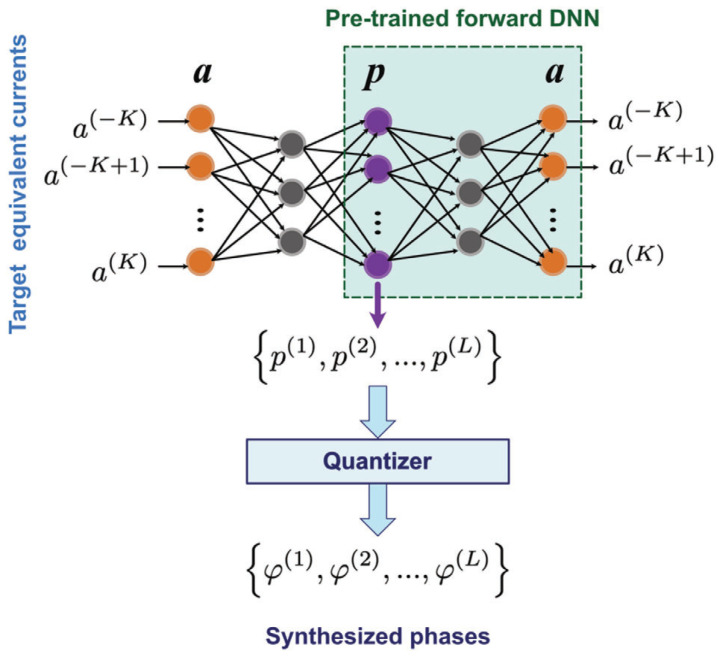
Illustration of the tandem DNN architecture [141].

Each harmonic pattern is thus represented by an array factor weighted by $\left\{a_{nm}^{\left(\nu \right)}\right\}$. A forward neural network learns the mapping from the temporal code to the harmonic coefficients, while an inverse network is trained so that the cascade reproduces prescribed coefficients under a global power-allocation constraint,

(90) $$P_{r}\leq \sum_{\nu =-K}^{K}∥a^{\left(\nu \right)}{∥}^{2}\leq 1,$$

which concentrates most radiated power within the selected harmonics. Figure 37 summarizes the forward and inverse learning pipeline that maps desired multi-harmonic current distributions to realizable time codes. By working at the level of harmonic currents, the method decouples multi-frequency synthesis into independent per-harmonic array designs and enables joint magnitude and phase control beyond prior STC schemes. Numerical examples show the accurate recovery of complex current distributions with low out-of-band spill.

**Figure 38:**
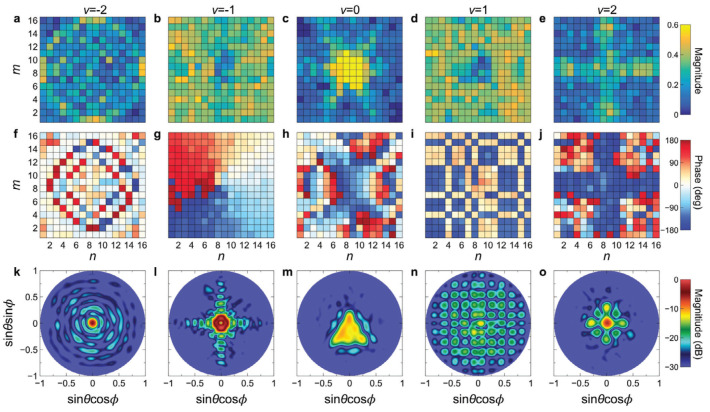
(**a**–**e**) Magnitude distributions for equivalent current at harmonic orders ν=−2,−1,0,1,2, (**f**–**j**) corresponding phase distributions, and (**k**–**o**) corresponding scattering patterns, with intensity normalized with respect to the maximum value at the harmonic order ν=−1 [141].

A 2-bit *X*-band prototype using varactor-loaded meta-atoms validates the method across five harmonics (ν=−2,−1,0,1,2). After compensating measured unit cell phase errors, the synthesized patterns closely match the targets, with higher-order spill remaining about 10 dB below the designed harmonics. Figure 38 reports the experimental validation [141], showing the target versus synthesized equivalent currents and the corresponding measured radiation patterns.

Beyond inference-only paradigms, recent work has clarified how physical neural networks implemented in wave-based and dynamical systems can be efficiently trained in the presence of fabrication imperfections, drift, and measurement noise. Modern training strategies increasingly emphasize hardware-aware and robustness-oriented optimization, moving beyond fixed-geometry reservoirs toward end-to-end learning of physical substrates [142,143]. These developments are particularly relevant for time-varying and spatiotemporally modulated metamaterials, where modulation depth, phase, and temporal waveforms naturally define a high-dimensional trainable parameter space. In this context, temporal boundaries and Floquet coupling between harmonics can act as structured learning mechanisms, enabling task-specific filtering, memory, and nonlinear mixing within physically constrained systems. As a result, space–time-modulated media are emerging not only as programmable devces, but also as adaptive, trainable computational platforms for analog signal processing and neuromorphic photonics.

The specific studies position AI as both a computational accelerator and a functional enabler for temporal metamaterials. Time-modulated media emerge not only as reconfigurable physical kernels for analog learning and reservoir computing, but also as fertile design spaces in which data-driven inverse synthesis can uncover strongly nonreciprocal and multifunctional responses beyond intuitive or brute-force methods. This convergence of space–time physics and machine learning opens a path toward self-optimizing, adaptive temporal platforms operating directly at the wave level.

## 10. Conclusions

This survey has provided a coherent and systematic mapping of time-varying and spatiotemporally modulated metamaterials across electromagnetic, acoustic, mechanical, and computational domains. By organizing a rapidly expanding and fragmented literature into unified theoretical categories, physical mechanisms, and application classes, we have shown that temporal modulation constitutes a genuine new axis of wave control rather than a mere extension of static metamaterial design. These phenomena include temporal boundaries, photonic time crystals, spatiotemporal nonreciprocity, temporal impedance matching, diffusive biasing, parametric amplification, and temporal cloaking. They originate from a common symmetry-breaking principle inherent to time-dependent material response. This unified viewpoint reveals deep analogies between systems that are traditionally treated as separate, including optics, acoustics, mechanics, and circuit networks, and it clarifies how time variation reshapes fundamental constraints imposed by reciprocity, passivity, and time-invariance. For clarity, Table 1 provides a compact summary of the main functional classes, underlying mechanisms, modulation strategies, representative platforms, and key references discussed throughout this survey.

Beyond consolidating the established results, this work highlights how temporal metamaterials are transitioning from proof-of-concept demonstrations toward robust, programmable, and integrable platforms. Advances in magnet-free isolation, broadband frequency conversion, temporal filtering, wave freezing, absorption beyond classical time-invariant bounds, and diffusive transport control already suggest practical impact in communications, signal processing, sensing, and energy systems. Experimental realizations based on transmission lines, metasurfaces, integrated photonic platforms, elastic beams, and programmable electrical lattices demonstrate that these concepts are not restricted to idealized theoretical settings. At the same time, the integration of machine learning with space–time-modulated media points to a new paradigm in which temporal structures function not only as physical devices but also as adaptive, data-driven computational substrates for analog signal processing and learning.

Several critical challenges remain open. These include the realization of large, ultrafast modulation depths at the nanoscale with acceptable pump efficiency; the management of loss, noise, and stability in strongly non-Hermitian temporal systems; and the mitigation of parasitic harmonics in broadband modulation schemes. Precise dispersion engineering remains essential for maintaining controllable phase matching over wide bandwidths, while scalable electronic and photonic control hardware is required for real-time programmability. Equally important is the establishment of fair benchmarking metrics that enable consistent comparison across disparate platforms and operating regimes. Addressing these issues will require coordinated progress across materials science, electronics, photonics, mechanics, numerical modeling, and control theory.

Beyond fundamental performance metrics, the transition of time-varying metamaterials from laboratory demonstrations to scalable technologies hinges on their compatibility with established fabrication and integration ecosystems. In this context, electro-optic and circuit-based platforms that leverage standard silicon photonics, CMOS-compatible processes, and wafer-scale integration currently offer the most direct “lab-to-fab” pathways, owing to their controllable modulation circuitry, mature packaging solutions, and reproducible performance. Conversely, platforms relying on exotic materials, extreme modulation speeds, or tightly coupled spatiotemporal biasing face higher barriers related to yield, thermal management, and control complexity.

From an application perspective, deployment in 6G communications will require high-power handling, low-loss nonreciprocity, and co-integration with RF front ends. LIDAR systems impose stringent constraints on optical loss, phase noise, and modulation bandwidth, while optical and neuromorphic computing demand long-term stability, device uniformity, and scalable control architectures. Addressing these requirements will require continued convergence between materials science, circuit co-design, and system-level optimization.

Looking forward, temporal metamaterials are poised to play a central role in next-generation wave-based technologies. Their ability to dynamically reshape the spectral, spatial, and even causal structure of wave propagation suggests opportunities well beyond conventional static metasurfaces, including real-time reconfigurable photonics, programmable analog computing, adaptive sensing, and hybrid classical information processing architectures. The extension of temporal concepts into the quantum regime and into coupled physical environments further broadens the horizon of possible applications. We expect that sustained cross-fertilization between fundamental theory, advanced fabrication, and emerging data-driven and AI-assisted design tools will continue to accelerate the maturation of the field and unlock functionalities that remain fundamentally inaccessible in time-invariant media.

## Figures and Tables

**Table 1:** Summary of representative functionalities enabled by temporal and spatiotemporal modulation, highlighting core mechanisms, implementation platforms, and level of experimental realization.

Category	Core Mechanism	Modulation Type	Platform	Functionality	Key References	Level of Experimental Realization
Foundations and theory	Temporal boundaries, time refraction and reflection, Floquet harmonics	Temporal	Electromagnetic waves	Frequency conversion, temporal scattering, energy exchange	[40,72,73]	Theory/numerical
Temporal crystals and band theory	Periodic temporal modulation, Floquet band formation	Temporal periodic	Photonic media	Momentum gaps, topological bands, parametric amplification	[23,43,91]	Theory/numerical/ early experiments
Nonreciprocity and isolation	Traveling-wave spatiotemporal modulation and momentum bias	Spatiotemporal	Transmission lines, resonators, metasurfaces	Magnet-free isolation, circulation, asymmetric mode conversion	[77,82,86]	Proof-of-concept experiments
Absorption and impedance matching	Temporal switching, KK/anti-KK duality, co-modulation of loss and dispersion	Temporal	Thin absorbers, transmission lines	Broadband absorption, reflection suppression	[83,84]	Proof-of-concept experiments
Frequency conversion and parametric processes	Indirect interband transitions, temporal phase accumulation	Temporal/ spatiotemporal	Cavities, ENZ materials, metasurfaces	Clean frequency translation, amplification, oscillation	[85,90]	Validated prototype
Beam control and routing	Temporal aiming, spatiotemporal momentum engineering	Temporal/ spatiotemporal	Anisotropic media, metasurfaces	Beam steering, routing, phase-insensitive combining	[31,97]	Proof-of-concept experiments
Diffusive spatiotemporal media	Phase-biased homogenization of diffusion parameters	Spatiotemporal	Electrical lattices, diffusive networks	Directed diffusion, trapping, bias without flow	[98]	Proof-of-concept experiments
Experimental prototypes	Varactor modulation, spatiotemporal impedance engineering	Spatiotemporal	RF circuits, metasurfaces	Duplexing, radiation, nonreciprocity	[105,106]	Validated prototype
Quantum, ultrafast, and AI-enabled platforms	Synthetic gauge phases, ultrafast modulation, physical learning kernels	Temporal/ spatiotemporal	Photonic crystals, metasurfaces	Quantum transitions, isolation, analog learning	[126,128,138]	Theory/numerical/ early experiments

## Data Availability

No new data were created or analyzed in this study.
